# Discovery of the first-in-class potent and isoform-selective human carbonic anhydrase III inhibitors

**DOI:** 10.1080/14756366.2023.2202360

**Published:** 2023-04-24

**Authors:** Simone Giovannuzzi, Alessandro Bonardi, Paola Gratteri, Alessio Nocentini, Claudiu T. Supuran

**Affiliations:** a NEUROFARBA Department, Pharmaceutical and Nutraceutical Section, University of Florence, Firenze, Italy; b NEUROFARBA Department, Laboratory of Molecular Modeling, Cheminformatics & QSAR, University of Florence, Firenze, Italy

**Keywords:** Drug design, metalloenzyme, inhibition, click chemistry, hCA III, molecular dynamics

## Abstract

Considering the unrecognised physio-pathological role of human carbonic anhydrase III (hCA III), a structure-based drug design was set up to identify the first-in-class potent and selective inhibitors of this neglected isoform. hCA III targeting was planned considering a unique feature of its active site among the other hCA isoforms, *i.e.* the Leu198/Phe198 substitution which interferes with the binding of aromatic/heterocyclic sulfonamides and other inhibitors. Thus, new aliphatic primary sulfonamides possessing long and flexible (CH_2_)_n_SO_2_NH_2_ moieties were designed to coordinate the zinc(II) ion, bypassing the bulky Phe198 residue. They incorporate 1,2,3-triazole linkers which connect the tail moieties to the sulfonamide head, enhancing thus the contacts at the active site entrance. Some of these compounds act as nanomolar and selective inhibitors of hCA III over other isoforms. Docking/molecular dynamics simulations were used to investigate ligand/target interactions for these sulfonamides which might improve our understanding of the physio-pathological roles of hCA III.

## GRAPHICAL ABSTRACT

**Figure:**
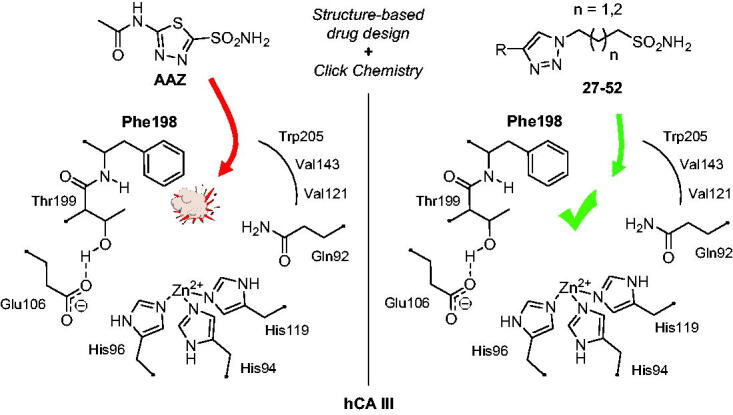

## Introduction

**Figure 1.**
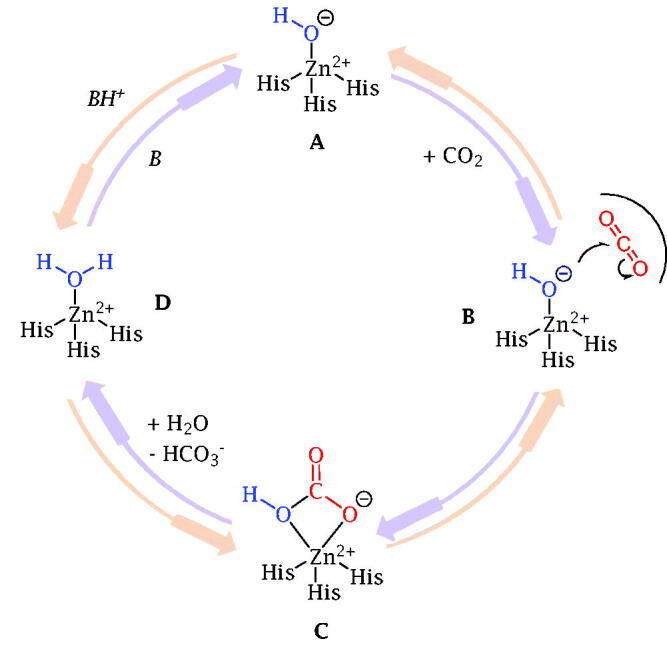
Schematic representation of the hCA catalytic mechanism.

Carbonic anhydrases (CAs), also known as carbonate dehydratases, are ubiquitous metalloenzymes present in prokaryotes and eukaryotes and encoded by eight evolutionarily unrelated gene families: α-CAs, in vertebrates, bacteria, algae and cytoplasm of green plants; β-CAs, predominantly in bacteria, algae and chloroplasts of monocotyledons and dicotyledons; γ-CAs, mainly in archaea and some bacteria; δ-CAs, in some marine diatoms; ζ-CAs, mainly in diatoms; η-CAs, in the protozoan *Plasmodium falciparum***;** θ-CAs, in diatoms; ι-CAs, recently discovered in bacteria and marine diatoms^1–15^. The class α includes all mammalian enzymes among which 15 isozymes identified in human (h) which differ for organ/tissue distribution and catalytic activity: 12 are catalytically active (hCAs I-IV, VA, VB, VI, VII, IX, XII e XIV), whilst 3 isoforms (hCAs VIII, X, XI), called CA-related proteins (CARPs), are devoid of catalytic activity^16–17^. hCAs catalyse in human a simple physiological reaction, that is the reversible hydration of carbon dioxide (CO_2_) to produce bicarbonate and proton which occurs by a two steps catalytic mechanism (Figure 1): (i) the nucleophilic attack of the zinc-bound hydroxide ion (A) to a CO_2_ molecule present in the enzymatic active site (B) to generate a HCO_3_^-^ ion (C), which is displaced by another H_2_O molecule (D); (ii) regeneration of catalytically active metal hydroxide species by a proton transfer reaction from the zinc-bound water to an exogenous proton acceptor or an active site residue^1–2^.

**Figure 2.**
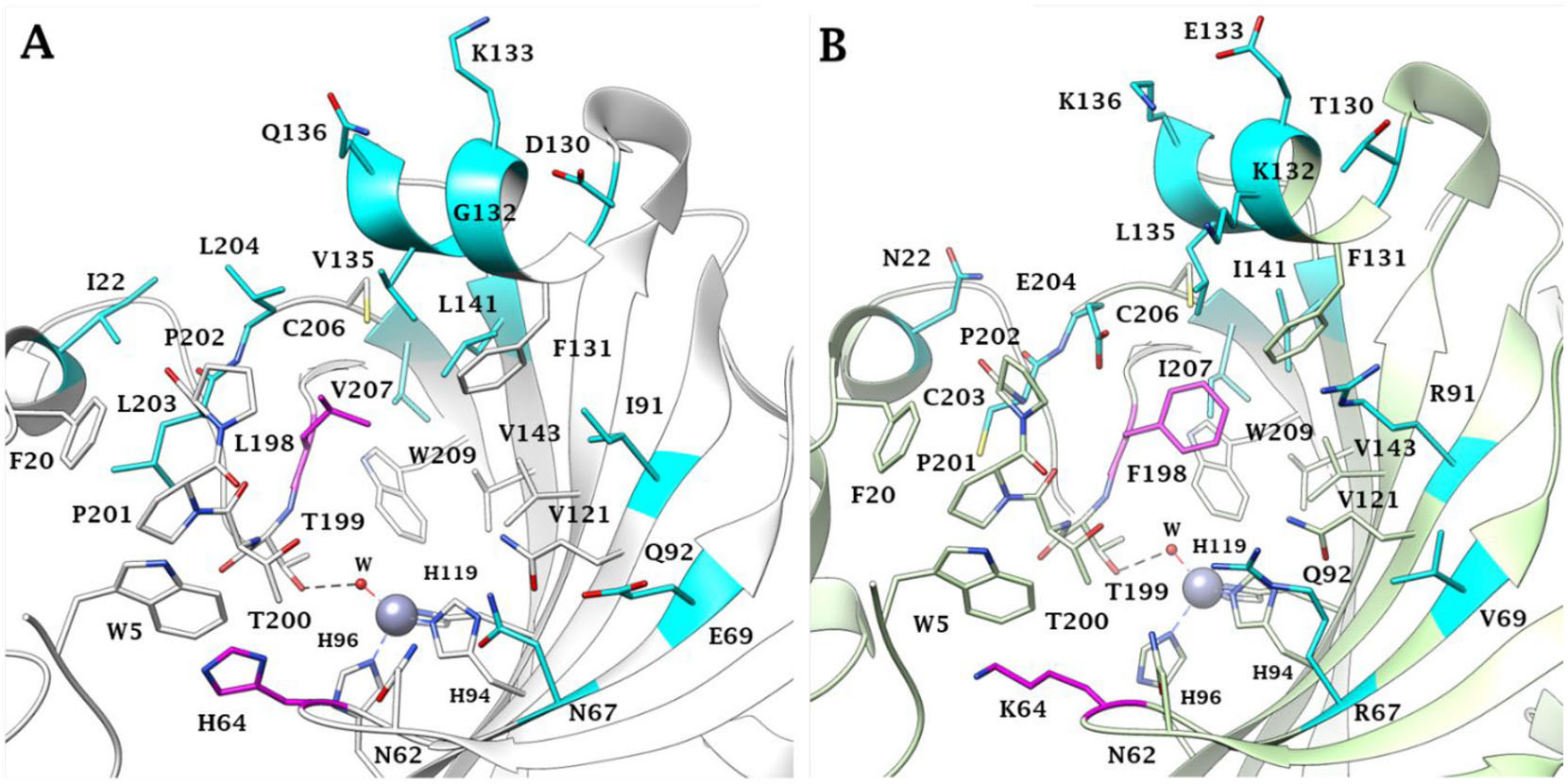
Active site view of A) hCA II (PDB: 3K34) and B) hCA III (PDB: 1Z93). The relevant His64/Lys64 and Leu198/Phe198 substitutions are labelled in magenta. All other different residues in the active sites are instead coloured cyan.

Notably, hCAs also differ for their response to enzyme modulators, among which zinc-binding compounds of the primary sulfonamide type are generally considered pan-inhibitors, lacking a relevant isoform selectivity (at least for the first- and second-generation inhibitors). In this context, hCA III stands out as unique among the catalytically active isozymes, because of its extremely low susceptibility to classic sulfonamide inhibitors, such as the reference drug acetazolamide (AAZ). Moreover, although hCA III has a high homology in the aminoacid sequences (62%) with the physiologically most relevant isoform, hCA II^18^, it possesses only the 0.3% of the CO_2_ hydration activity exhibited by hCA II^15^. These divergences have been correlated to the presence of several amino acid mutations in the hCA III active site with respect to most other hCAs (Figure 2), among which 2 are crucial: Lys64 and Phe198, in place of His64, the proton shuttle residue, and Leu198, respectively (Figure 2). It was shown that histidine is much more efficient than lysine in the proton shuttle mechanism, the rate-limiting step in the catalysis of CO_2_ hydration^19–20^. As a result, it is reasonable that the His to Lys swap produces a dramatic loss of catalytic efficiency compared to other hCA isoforms such as hCA II. On the other side, Leu198 contributes to form the active site hydrophobic pocket essential for binding CO_2_ in the neighbourhood of the nucleophilic zinc-bound water. As demonstrated by kinetic and structural evidence^21–23^, the phenyl ring of Phe198 produces a steric constriction within the active site negatively affecting interactions with the zinc-bound nucleophile, which further results in a decrease of the catalytic activity of hCA III. As well, interactions of classical CAIs, such as sulfonamides, is relevantly hindered in hCA III by the Leu198/Phe198 substitution.

**Figure 3.**
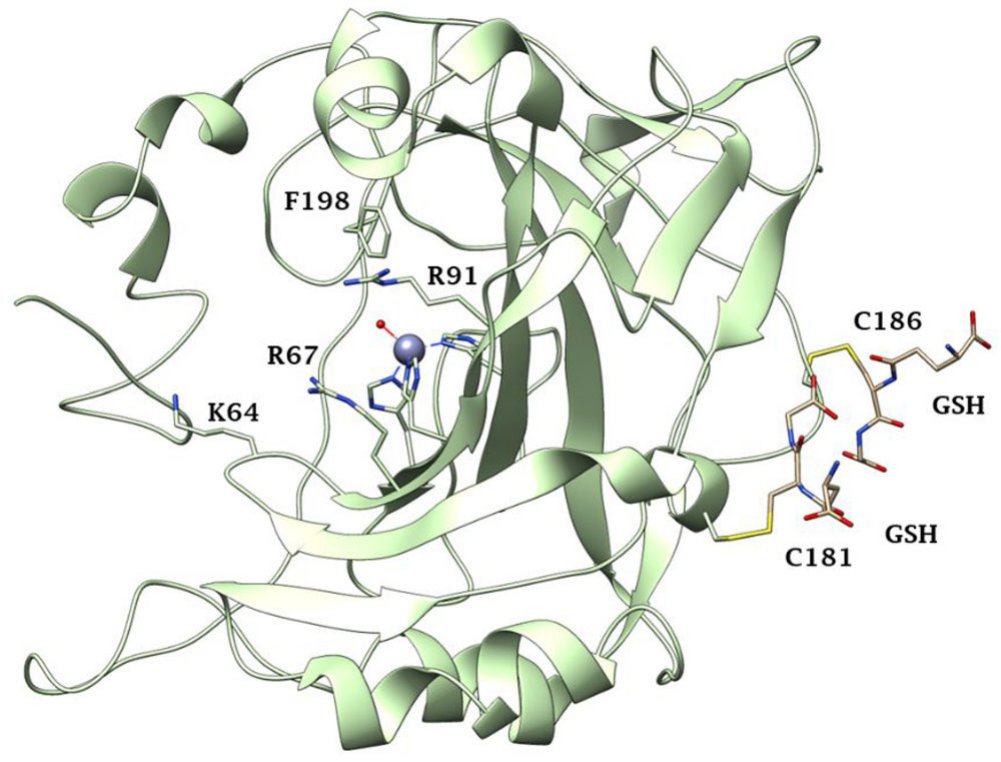
Ribbon view of CA III from rat liver with the S-glutathionylation sites (GSH) displayed on its surface (PDB 1FLJ).

hCA III is highly expressed in skeletal muscles, adipose tissue, liver and brain. Several suggestions have been made about the physiological role of hCA III, but, up to now, its function results uncertain^24–26^. The lower activity and the muscle localisation suggest that hCA III may be working in an oxidising environment which led to its post-translational modifications, allowing it to operate as a cellular antioxidant^27–28^. Indeed, hCA III incorporates two reactive surface cysteines, C183 and C188, which are not present in other cytosolic isoforms, such as hCA I and II (Figure 3). They were shown to be S-glutathiolated both *in vitro* and *in vivo*^29–30^. The S-glutathionylation activity does not impair the catalytic activity of hCA III^31^. Recently, the potential involvement of hCA III in several cancer types has been discovered, among which oral squamous cell cancer, which is the result of epithelial-mesenchymal transition (EMT), a process converting epithelial cells into mesenchymal cells^32–33^. EMT-related molecules are connected to invasion and metastasis in oral cancer, as reported by several studies^34–36^. Dai et al., in 2007^37^, demonstrated that hCA III promotes the cell invasion capability in hepatocellular carcinoma cells through the FAK signalling pathway. Moreover, hCA III might increase the expression of the mesenchymal markers and several transcription factors to stimulate cell invasion and migration abilities. A few years later, a new study demonstrated the potential of hCA III to promote invasion abilities through active FAK signalling pathways^37^^,^^38^. Thereafter, hCA III has been evaluated as a biomarker in various diseases like neuromuscular disease, sarcopenia, hepatitis B and C infections and liver carcinoma^37^^,^^39–40^.

In order to investigate the role of this enzyme in several pathologic or physiologic processes, it is crucial to have effective and isoform-selective CA III inhibitors, which do not exist at the moment. The present work proposes a simple yet innovative drug design strategy that led to the first-in-class hCA III potent and selective inhibitors, based on the specific features of this isoform outlined above: the presence of the bulky Phe198 in the middle of the active site cavity.

## Results and discussion

### Drug design and chemistry

**Figure 4.**
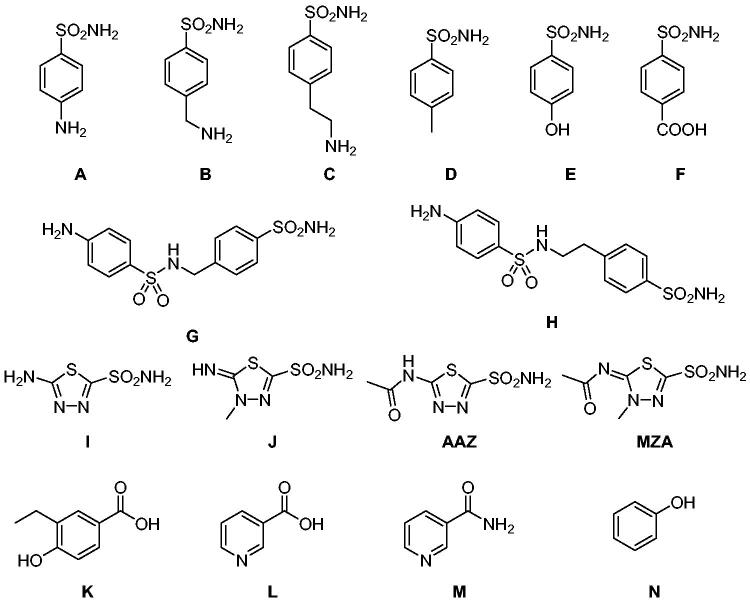
Compounds tested as hCA III inhibitors by Nishimori et al. in 2007 and Alzewiri et al in 2015^43^^,^^45^.

The most investigated CAI chemotypes, that are primary sulfonamides (-SO_2_NH_2_), sulfamides (-NHSO_2_NH_2_), and sulfamates (-OSO_2_NH_2_), are not effective inhibitors of hCA III^41–43^. In 2007 Nishimori et al. investigated the inhibitory potency of a library of sulfonamides and one sulfamate against the newly expressed hCA III. The compound set included both benzenesulfonamide (**A-H**) and thiadiazole derivatives, such as **AAZ** and methazolamide (**MZA**), and their deacylated derivatives **I** and **J** (Figure 4). All these molecules inhibited hCA III in the low to high micromolar range^43^. For instance, the reference drug **AAZ** showed an inhibition constant (K_I_) against hCA III of 236 µM.

Alzweiri et al. in 2015 obtained interesting hCA III inhibitory profiles with a series of benzoic acid and nicotinic acid derivatives by using an HPLC assay^44–45^. For instance, vanillic acid (**K**) showed a rather potent hCA III inhibitory activity than AAZ, with a K_I_ of 87.0 µM, which is anyhow the behaviour of a weak inhibitor^46^. However, the binding modes of these inhibitors to hCA III were not investigated by crystallographic or other structural studies.

**Figure 5.**
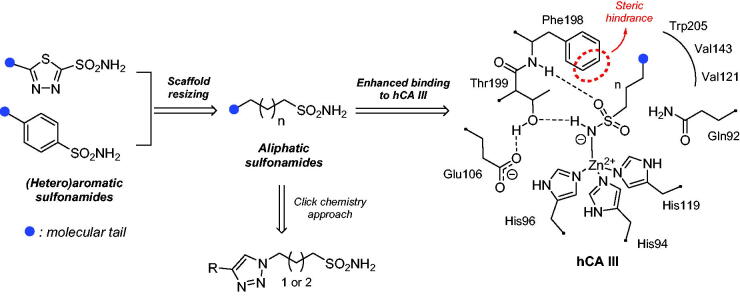
Drug design strategy proposed here to attain potent and selective hCA III inhibitors.

To better understand the physiological and pathological role of hCA III, more effective and selective inhibitors of this isoform are needed. Upon a structure-based *in silico* analysis of the active sites of hCA III and hCA II (as a representative of all other hCA isoforms, Figure 2), it was clear that the Leu198/Phe198 substitution represents a significant hindrance for classical aromatic/heteroaromatic primary sulfonamides, the most potent and studied CAIs, to properly reach and coordinate the zinc(II) ion. Herein, a resizing of the scaffold bearing the metal binding function (-SO_2_NH_2_) was designed (Figure 5), swapping from a (hetero)aromatic ring to a 3- or 4-carbon atoms aliphatic chain, which in turn supports the inhibitor tail to increase the ligand/target interactions. A 1,2,3-triazole linker was also incorporated, in order to include a variety of molecular pendants onto the main alkyl primary sulfonamide scaffold, among which carboxylates or substituted phenyl groups connected directly or by ether/ureido spacers.

**Scheme 1.**
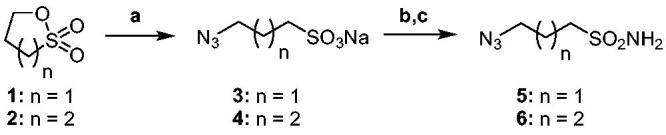
Synthesis of ω-azidealiphatic sulfonamides **5** and **6**. Reagents and conditions: a) NaN_3_, H_2_O/acetone, rt, 4h. b) SOCl_2_, 60 °C, o.n.; c) NH_4_OH, dry THF, 0 °C to rt, 3h.

**Scheme 2.**
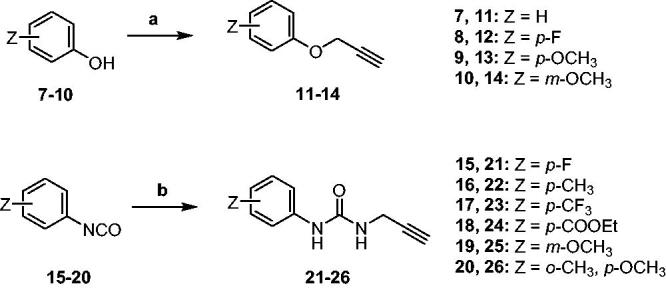
Synthetic approach for the alkyne intermediates **11–14** and **21–26**. Reagents and conditions: a) propargyl bromide, K_2_CO_3_, dry DMF, 80 °C, 6h; b) propargyl amine, DIPEA, dry ACN, rt, 3h.

**Scheme 3.**
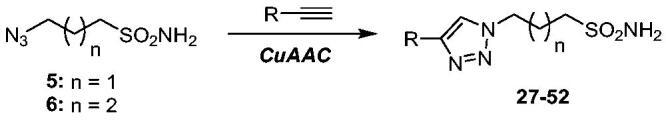
Synthesis of 1,2,3-triazole derivatives **27–52**. CuAAC conditions: Cu(CH_3_COO)_2_/sodium ascorbate, MeOH/THF, 40 °C, o.n.

The Cu(I)-catalysed azide-alkyne cycloaddition (CuAAC or Click Chemistry) was adopted as a synthetic tool to generate the 1,2,3-triazole linker which connects the main scaffold and the molecular tails. As such, aliphatic sulfonamides bearing an azide moiety were obtained upon 3-propanesultone (1) or 4-propanesultone (2) ring opening with sodium azide to yield sulfonates **3** and **4** that were converted to the corresponding primary sulfonamides **5** and **6** by chlorination with thionyl chloride and subsequent treatment with an ammonia 28% aqueous solution in THF (Scheme 1). The alkyne reagents used in the CuAAC reaction with azides **5** and **6** were commercially available or freshly prepared by the synthetic pathways reported in Scheme 2. In detail, phenols **7–10** were reacted by a nucleophilic substitution with propargyl bromide in presence of potassium carbonate in dry DMF to achieve alkyne intermediates **11–14**. Instead, phenyl isocyanates **15–20** were treated with propargyl amine in dry acetonitrile to afford urea derivatives **21–26**. The CuAAC reactions to give the 1,2,3-triazole compounds **27–52** with high yields and purity were conducted using Cu(CH_3_COO)_2_ and sodium ascorbate to generate the Cu(I) catalyst in a MeOH/THF solvent mixture (Scheme 3).

### Carbonic anhydrase inhibition

The CA inhibition profiles of compounds **27–52** were thus evaluated against eight physiologically relevant isoforms, hCA I, II, III, IV, VA, VB, VII and XII by a Stopped-Flow CO_2_ hydrase assay ^47^, using **AAZ** as reference inhibitor (Table 1). The following structure-activity relationship (SAR) can be gathered from the inhibition data reported in Table 1.

**Table 1.** Inhibition data of human CA isoforms I, II, III, IV, VA, VB, VII and XII by a Stopped-Flow CO_2_ hydrase assay^47^, using **AAZ** as reference inhibitor. 
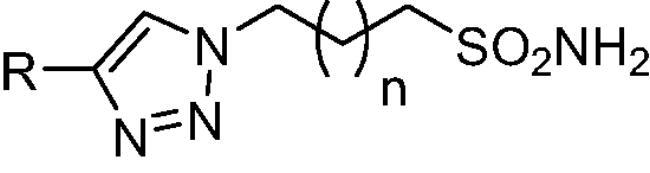

cmp	R	n	**K_I_ (nM)** ^a^							
			hCA							
			I	II	III	IV	VA	VB	VII	XII
**27**	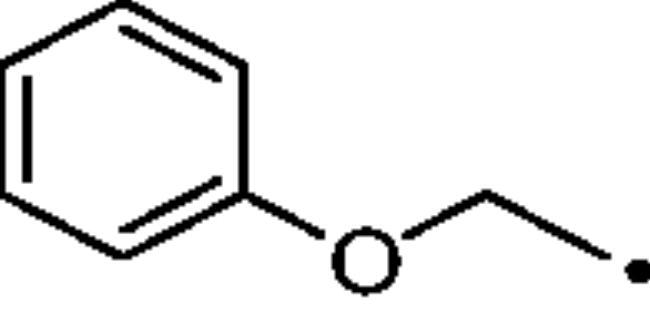	3	759.9	2720.2	653.9	4721.1	3870.1	2885.8	2695.6	2693.3
**28**	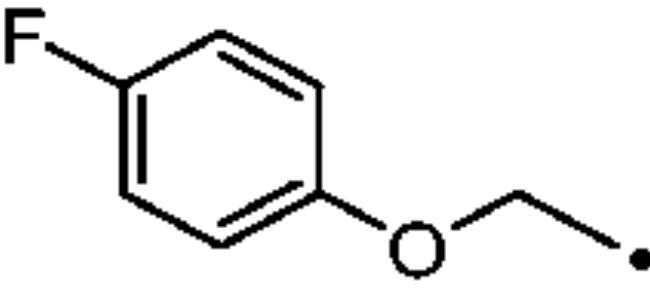	3	4890.2	2026.2	727.9	>10000	1033.6	2466.4	4365.8	2477.1
**29**	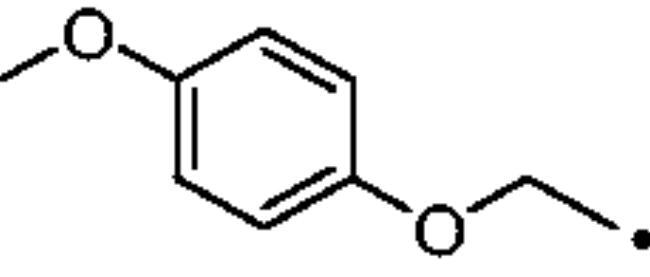	3	1160.9	2446.3	553.6	5436.8	4810.6	3027.8	2710.7	1954.5
**30**	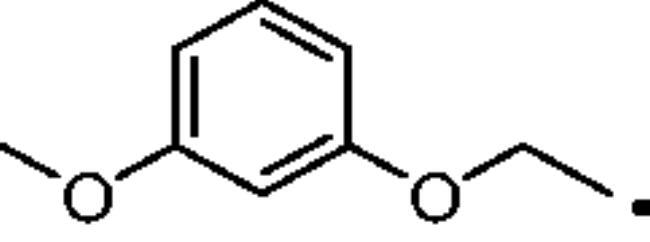	3	1914.4	4102.0	345.0	>10000	2881.3	1445.0	4684.5	1096.0
**31**	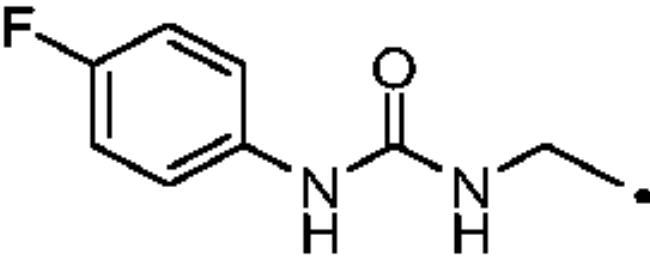	3	>10000	2898.3	825.7	5926.3	5702.2	5866.1	4573.5	1504.3
**32**	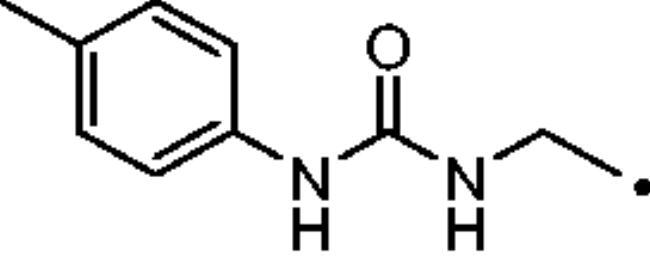	3	>10000	2968.7	772.1	6155.5	8696.4	1610.9	4396.2	5033.6
**33**	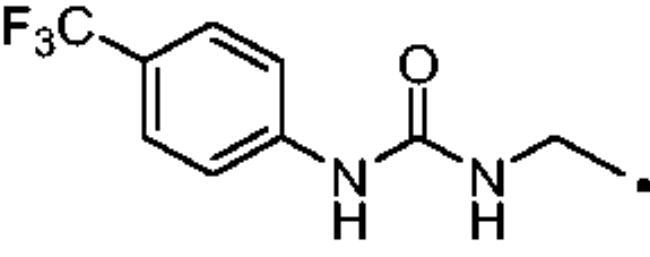	3	>10000	4162.5	988.6	875.2	7043.2	2093.2	5411.1	2631.6
**34**	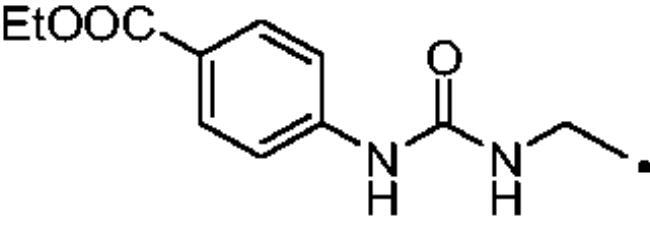	3	>10000	2503.4	546.8	4457.1	2654.4	3738.2	2309.0	4896.3
**35**	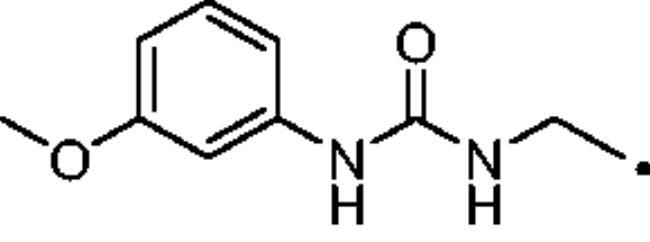	3	>10000	936.9	786.4	>10000	2042.5	3997.3	4475.2	1978.2
**36**	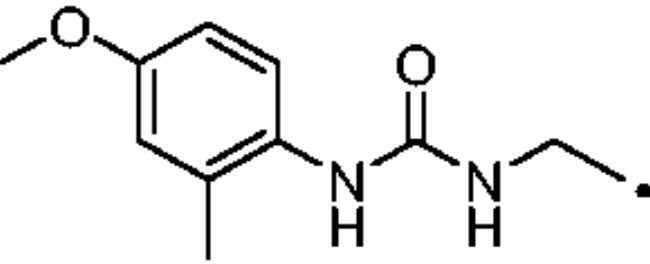	3	>10000	879.3	990.3	1592.8	2963.8	1296.5	1808.5	3269.9
**37**	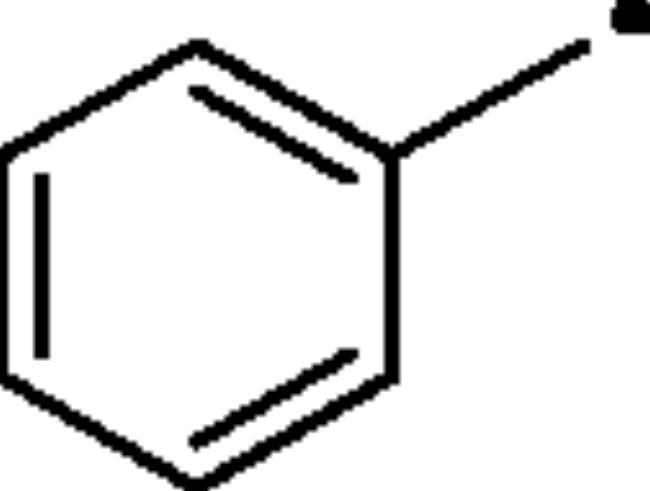	3	625.6	657.9	629.5	811.8	891.3	510.0	854.8	1360.9
**38**	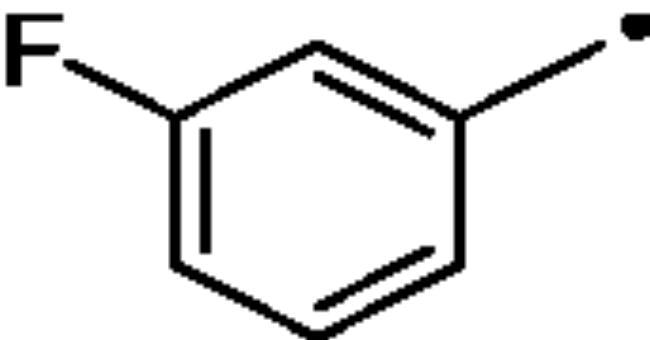	3	607.1	712.7	502.3	959.0	1654.8	1036.3	859.8	1059.4
**39**	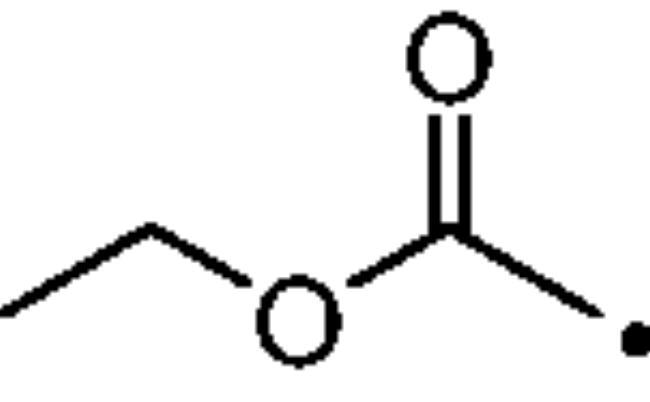	3	861.8	3145.0	162.6	2605.2	1570.7	1314.7	6497.3	1244.5
**40**	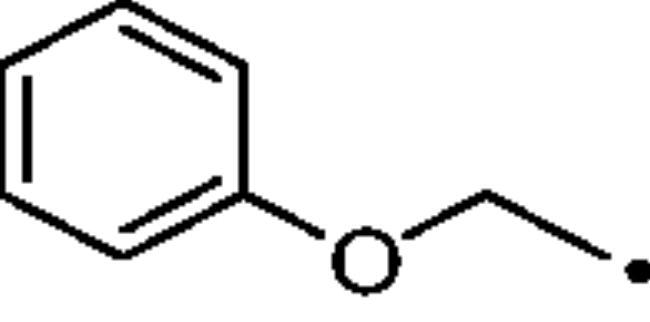	4	6539.4	7767.7	817.6	3607.6	7784.0	1528.0	7612.2	5175.6
**41**	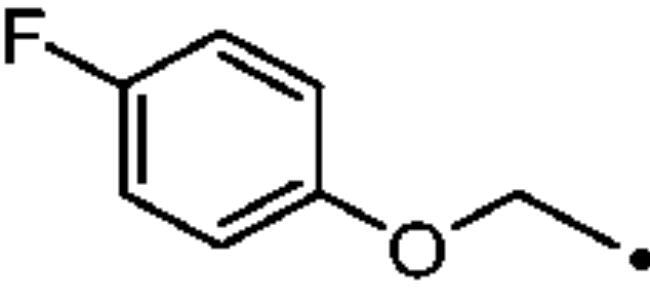	4	6429.8	8127.0	618.5	5507.6	4392.0	1242.4	5328.2	2102.9
**42**	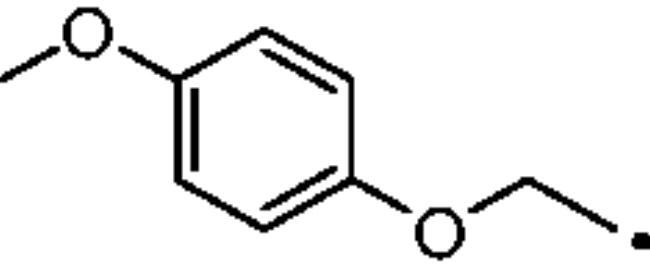	4	8541.9	3932.6	675.8	5608.4	6040.5	6673.4	5297.3	3067.5
**43**	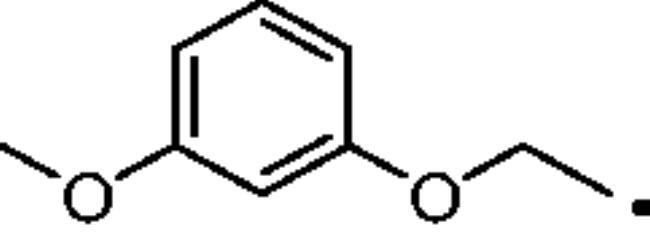	4	2315.4	5973.4	444.4	8270.3	2169.7	1629.7	3647.0	2724.3
**44**	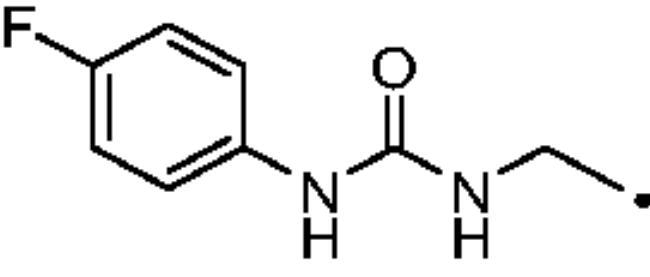	4	5380.2	7577.3	598.6	2919.4	1347.5	2550.0	3347.8	3713.9
**45**	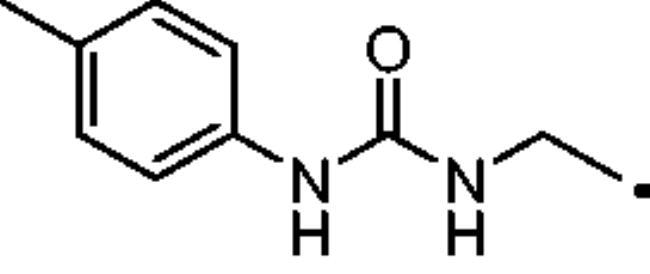	4	5296.2	4933.1	930.0	5736.4	3337.9	8114.1	5639.1	6731.1
**46**	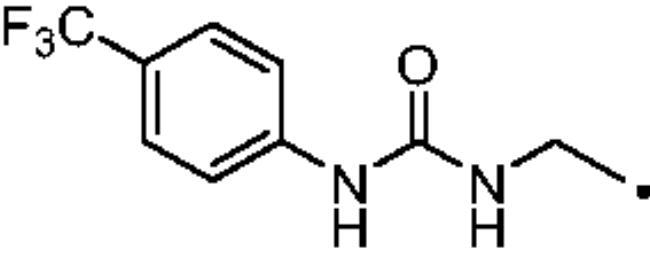	4	5387.9	878.7	552.7	3594.0	1804.0	5872.4	3608.7	2625.0
**47**	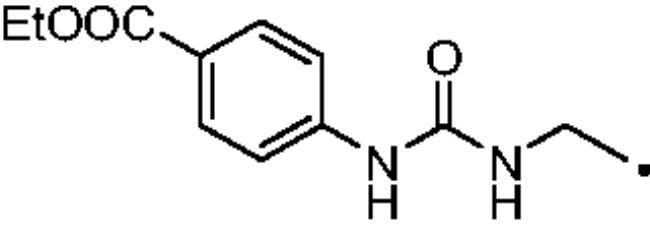	4	6524.1	566.0	493.4	2601.8	3820.3	1430.6	1947.3	2834.6
**48**	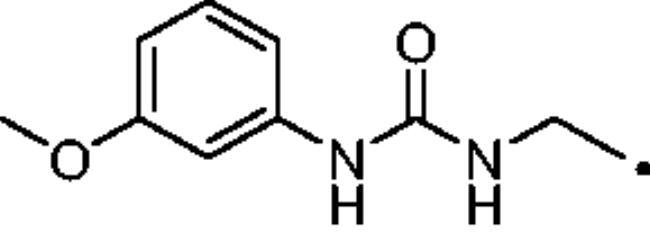	4	5518.6	4605.9	476.0	>10000	4159.1	1144.3	3896.5	4660.6
**49**	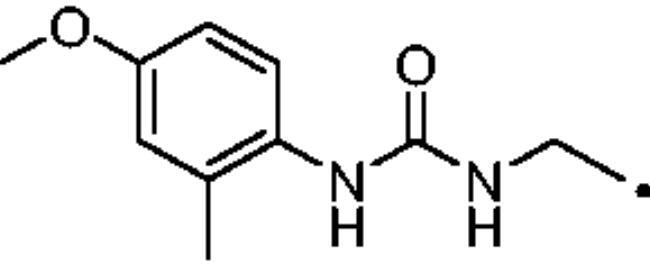	4	>10000	8157.5	795.4	5804.4	8774.2	9210.8	5146.4	5821.4
**50**	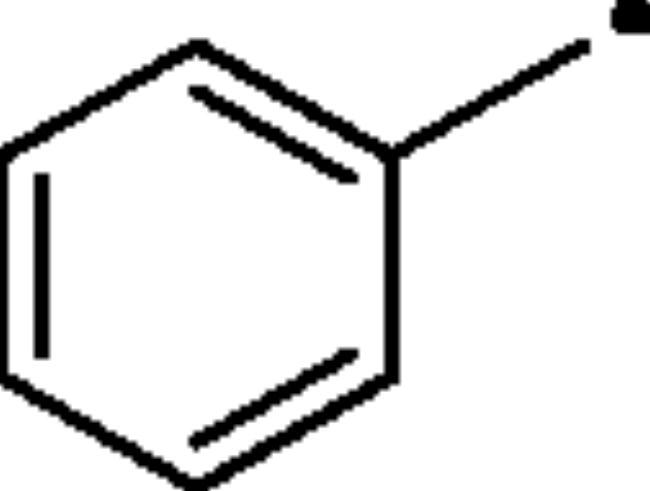	4	915.8	771.6	775.0	3984.6	911.5	814.0	809.3	1021.6
**51**	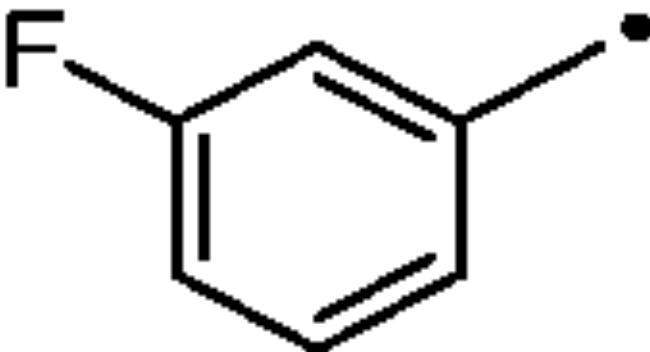	4	3274.5	803.0	601.5	9311.6	1484.4	1922.2	1657.9	3846.9
**52**	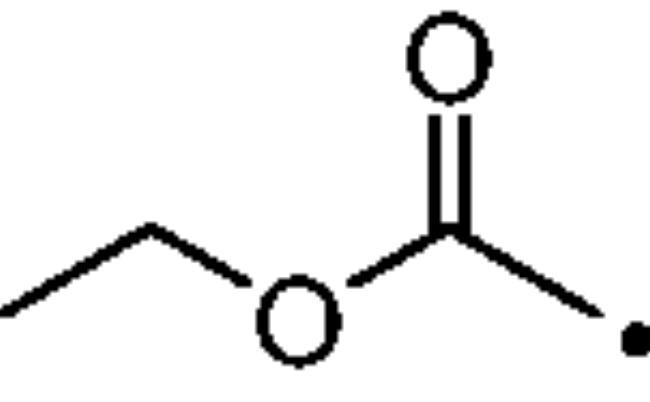	4	778.4	8132.3	310.4	>10000	1268.6	2989.3	3230.0	7811.9
**AAZ**	–	–	250.0	12.0	236000	74.0	63.0	54.0	2.5	5.7

^a^Mean from 3 different assays, by a stopped-flow technique (errors were in the range of ± 5–10% of the reported values).

**Table 2.** Selectivity Index (*SI*) of 1,2,3-triazoles with aliphatic sulfonamide between hCA I and hCA II against hCA III.

Cmpd	Selectivity Index (*SI*)	
	hCA I/hCA III	hCA II/hCA III
**27**	1.2	4.2
**28**	6.7	2.8
**29**	2.1	4.4
**30**	5.5	11.9
**31**	12.1	3.5
**32**	12.9	3.8
**33**	10.1	4.2
**34**	18.3	4.6
**35**	12.7	1.2
**36**	10.1	0.9
**37**	1.0	1.0
**38**	1.2	1.4
**39**	5.3	19.3
**40**	8.0	9.5
**41**	10.4	13.1
**42**	12.6	5.8
**43**	5.2	13.4
**44**	9.0	12.7
**45**	5.7	5.3
**46**	9.7	1.6
**47**	13.2	1.1
**48**	11.6	9.7
**49**	12.6	10.3
**50**	1.2	1.0
**51**	5.4	1.3
**52**	2.5	26.2
**AAZ**	1.06 × 10^−3^	5.08 × 10^−5^

- The cytosolic hCA I was oddly among the most inhibited isoforms, with K_I_s between 607.1 nM and 8541.9 nM. It shows different inhibition data based on the linker present in all products. In fact, the most potent inhibitors, in the range of high nanomolar, are compounds without a specific functional group (urea or ether) in the tail. The most potent are **37** (K_I_ = 625.6 nM) and **38** (K_I_ = 607.1 nM) in which the R substitution is directly connected to the triazole moiety, and **27** (K_I_ = 759.9 nM), the simplest ether derivate. Considering butanesulfonamide as an aliphatic chain, only number **52**, showing R = ethyl ester, has a K_I_ in the high nanomolar range, 778.4 nM. Generally, the lengthening of the aliphatic chain, from propanesulfonamide to butanesulfonamide, produces a CA I inhibition impairment. Indeed, products **27**–**30** (K_I_ = 759.9–4890.2 nM) are more effective inhibitors than compounds **40**–**43** (K_I_ = 2315.4–8541.9 nM). The presence of the urea moiety in the tail shows a reversal trend. Thus, compounds **44**–**49** (K_I_ = 5296.2–10000 nM) showed a better affinity than the corresponding derivatives **31**–**36** (K_I_ >10000 nM), which were the least effective CA I inhibitors. Specifically, **44**–**49** show a small inhibition improvement when the phenyl ring has only one substituent, such as in compound **45** with a methyl group, K_I_ of 5296.2 nM. On the other hand, a double substitution yields an inactive inhibitor, as **49** with a K_I_ >10000 nM. Finally, compounds with a less bulky R substituent show the most potent inhibition on hCA I as earlier described.
- On cytosolic hCA II these compounds showed K_I_ values between 566.0 nM and 8157.5 nM. As compared to hCA I, all the urea derivatives demonstrated a major affinity on hCA II, probably due to the larger active site of hCA II compared to hCA I. Products **31**–**36** showed the most effective improvement of potency, from K_I_ > 10000 nM on hCA I to K_I_ of 879.3–4162.5 nM against hCA II. Particularly, **35** (K_I_ = 936.9 nM) and **36** (K_I_ = 879.3 nM) have K_I_ values in the high nanomolar range. The corresponding butanesulfonamide derivates, **44**–**49**, maintain their inhibition observed on hCA I, except **46** and **47** showing a strong improvement, respectively 878.7 nM and 566.0 nM. Probably, the presence of bulky groups, which can improve the interactions with the active site, promotes the inhibition of hCA II over hCA I. Particularly, methoxy substituents decrease the inhibition potency as in **35**–**36** (K_I_ = 936.9 nM and 879.3 nM) and **48**–**49** (K_I_ = 4605.9 nM and 8157.5 nM). Considering compounds without specific functional groups (urea or ether) in the tail, such as **37**–**39** and **50**–**52**, there is little inhibition difference data based on the lengthening of the aliphatic chain. As mentioned, **37** and **50**, **38** and **51** show similar K_I_ values, 657.9–771.6 nM and 712.7–803.0 nM. On the other side, less-bulky compounds, such as **39** and **52,** demonstrated a remarkable affinity difference, of 3145.0 nM and 8132.3 nM, respectively. Generally, the presence of bulky substituents produced compounds with a medium inhibition potency on hCA II as demonstrated by **36** (K_I_ = 879.3 nM), **46** (K_I_ = 878.7 nM) and **47** (K_I_ = 566.0 nM).
- The cytosolic hCA III was the most inhibited isoform by the panel of tested CAs. All the aliphatic sulfonamides showed a nanomolar inhibition constant, ranging from 162.6 nM (low nanomolar range) to 990.3 nM (high nanomolar range). The length of the aliphatic chain seems to be important for products with ether as a linker. In fact, compounds **27**–**30** are usually better inhibitors compared to the corresponding **40**–**43.** Compound **41** (K_I_ = 618.5 nM) showed a higher affinity than its analogue **28** (K_I_ = 727.9 nM), incorporating a propanesulfonamide moiety as the aliphatic chain. Generally, the presence of substituents on the phenyl ring improved inhibition as observed for **27** (K_I_ = 653.9 nM), **29** (K_I_ = 553.6 nM), **40** (K_I_ = 817.6 nM) and **42** (K_I_ = 675.8 nM). The shift of the methoxy group from a *p*-substitution to a *m*-substitution produced an improvement of the potency against hCA III, as demonstrated by **29**–**30** and **42**–**43**, with K_I_ = 553.6–445 nM and K_I_ = 675.8–444.4 nM, respectively. Considering the derivatives with a ureido linker, **44**–**49** (K_I_ = 476.0–930.0 nM), they showed lower K_I_ values compared to their propanesulfonamide analogues **31**–**36** (K_I_ = 546.8–990.3 nM). Particularly, compounds **46**, **47** and **48** have K_I_ in the medium nanomolar range, 552.7 nM, 493.4 nM and 476.0 nM. In fact, **34** is the only ureidic compound having a propanesulfonamide chain which shows a K_I_ value in the medium nanomolar range, 546.8 nM. The presence of halogens on the phenyl ring produces an improvement of the inhibition properties, especially with the butanesulfonamide chain, as demonstrated by **44**, K_I_ = 598.6 nM, and **46**, K_I_ = 552.7 nM. The most effective substitution pattern seems to be the polar and bulky ethyl ester showing both aliphatic chains (**34**: K_I_ = 546.8 nM; **47**: K_I_ = 493.4 nM). As expected, the less-bulky derivates were the most effective inhibitors of hCA III, probably due to the steric hindrance in the active site of hCA III. As described earlier, the presence of a halogen improves the potency of **38** (K_I_ = 502.3 nM) and **51** (K_I_ = 601.5 nM) compared to the corresponding non-halo derivatives **37** (K_I_ = 629.5 nM) and **50** (K_I_ = 775.0 nM). Thanks to the bulky group absence, compounds **39** and **52** were the best inhibitors against hCA III in this series, with K_I_ in the medium-low nanomolar range, respectively of 162.6 nM and 310.4 nM.
- On the membrane-associated hCA IV, these inhibitors showed K_I_ values between 811.8 nM and >10000 nM. Products **28** and **30** did not inhibit this isoform (K_I_ > 10000 nM), but the corresponding derivatives with the butanesulfonamide chain, **41** and **43**, showed activity against hCA IV, with K_I_s of 5507.6 nM and 8270.3 nM, respectively. The presence of a methoxy group in position 4 of the phenyl ring, compared to position 3, leads to an enhancement of inhibition potency as demonstrated by **29**, K_I_ = 5436.8 nM, and **32**, K_I_ = 5608.4 nM. The absence of substituents on the aromatic ring produced the best products with the ether in the tail, **27**, K_I_ = 4721.1, and **40**, K_I_ = 3607.3 nM. Considering the ureidic portion in the tail and the propanesulfonamide aliphatic chain, compound **33** demonstrated a better inhibition, in the high nanomolar range, 875.2 nM, whereas the replacement of the CF_3_ substituent with F or CH_3_ group led to less effective inhibitors. Indeed, product **31** with a F atom has a K_I_ of 5936.3 and **32** with CH_3_ group has K_I_ = 6155.5 nM. The double substitution on the phenyl ring produced one of the most potent inhibitors on hCA IV, such as **36**, with a K_I_ of 1592.4 nM. The lengthening of the aliphatic sulfonamide chain generates compounds with K_I_s between 2601.8 nM and >10000 nM. Compound **48** with a methoxy group in *meta* position on the phenyl ring showed the worst K_I_ value, similar to its analogue **35** (K_I_ > 10000 nM). Sulfonamides **44** and **47**, with an F atom and an ethyl ester in the *para* position of the phenyl ring, respectively, showed K_I_ values in the low micromolar range, 2919.4 nM and 2601.8 nM, whereas a double substitution decreased the affinity against hCA IV (**49**: K_I_ = 5804.4 nM). After that, compounds **37** and **38** had K_I_ values in the high nanomolar range, of 811.8 nM and 959.0 nM, respectively. The lengthening of the aliphatic sulfonamide chain impaired the inhibition to the medium-high micromolar range (**50**: K_I_ = 3984.6 nM; **51**: K_I_ = 9311.6 nM).
- The mitochondrial hCA VA and VB are two of the less effectively inhibited isoforms of the panel of hCA tested here. All the compounds showed K_I_ in the micromolar range on hCA VA 1033.6–8774.2 nM and hCA VB 1036.3–9210.8 nM, except **37** (hCA VA: K_I_ = 891.3 nM; hCA VB: K_I_ = 510.0 NM) and **50** (hCA VA: K_I_ = 911.5 nM; hCA VB: K_I_ = 814.0 nM), that demonstrate a nanomolar affinity against both isoforms. The halogen incorporation on the terminal phenyl ring of these molecules, such as a F atom, impaired inhibitory power. In fact, **38** and **51** showed K_I_ values in the low micromolar range, respectively of 1654.8 and 1484.4 nM on hCA VA and 1036.3 nM and 1922.2 nM on hCA VB. Considering products **27**–**30** and **40**–**43**, the presence of a halogen on the phenyl ring led to an inhibition improvement on hCA VA and VB as demonstrated by **28** (hCA VA: K_I_ = 1033.6 nM; hCA VB: K_I_ = 2466.4 nM) and **41** (hCA VA: K_I_ = 4392.0 nM; hCA VB: K_I_ = 1242.4 nM), compared to **27** (hCA VA: K_I_ = 3870.1 nM; hCA VB: K_I_ = 2885.8 nM) and **40** (hCA VA: K_I_ = 7784.0 nM; hCA VB: K_I_ = 1528.0 nM). The lengthening of the sulfonamide aliphatic chain enhanced the affinity for hCA VB over VA, particularly for compound **40**, which had K_I_s of 7784.0 nM on hCA VA to 1528.0 nM on hCA VB. Products **29** (hCA VA: K_I_ = 4810.6 nM; hCA VB: K_I_ = 3027.8 nM) and **42** (hCA VA: K_I_ = 6040.5 nM; hCA VB: K_I_ = 6673.4 nM), incorporating a methoxy group in the *para* position, have big differences in K_I_ values. Considering the urea moiety in the tail, all the products showed K_I_ values in the range between low to high micromolar. Compounds **36** (hCA VA: K_I_ = 2963.8 nM; hCA VB: K_I_ = 1296.5 nM) and **44** (hCA VA: K_I_ = 1347.5 nM; hCA VB: K_I_ = 2550.0 nM) have an acceptable potency on both mitochondrial isoforms. Generally, an improvement of inhibition on hCA VB over hCA VA was observed for **31**–**36**, whereas for compounds **44**–**49,** the lengthening of the aliphatic chain impaired the affinity on hCA VB over hCA VA. Derivative **49** is the least effective inhibitor of the series, showing K_I_ values in the high micromolar range, of 8774.2 nM on hCA VA and 9210.8 nM on hCA VB.
- hCA VII was also less effectively inhibited among the investigated isoforms by aliphatic sulfonamides reported here, with K_I_ values in the range of high nanomolar to high micromolar. The most potent inhibitors **37**, **38** and **50** showed acceptable potency against this isoform, with K_I_s of 854.8 nM, 859.8 nM and 809.3 nM, respectively. The inhibition data related for **51** (K_I_ = 1657.9 nM) demonstrates unfavourable effects due to the presence of a halogen coupled to the butanesulfonamide chain, which induced a reduction of affinity against this isoform. Considering **27**–**30** and **40**–**43** with ether as a functional group in the tail, the presence of a methoxy group on the terminal aromatic ring increased the potency of the inhibitors, as observed for **29**, K_I_ = 2710.7 nM, and **43**, K_I_ of 36470 nM. Product **27**, without substituents on the phenyl ring, showed one of the best inhibition values on hCA VII, 2695.6 nM, whereas, the lengthening of the aliphatic chain, from three to four carbons, **40** (K_I_ = 7612.2 nM), or the insertion of a halogen on the phenyl ring, **28** (K_I_ = 4365.8 nM), impaired the inhibition. Considering ureidic compounds, rather effective inhibitors were **34** (K_I_ = 2309.0 nM) and **47** (K_I_ = 1947.3 nM). They incorporate an ethyl ester on the terminal phenyl ring which led to the most effective inhibitors. Considering compounds with the longer aliphatic sulfonamide chain, such as **44** and **46**, the presence of a halogen on the terminal aromatic ring improved inhibition capacity, respectively 3347.8 nM and 3608.7 nM.
- The membrane-associated hCA XII, similar to CA VII was also poorly inhibited, with K_I_ values from low to the high micromolar ranges, 1021.6 nM to 7811.9 nM. Considering the ether compounds, the presence of substituent on the phenyl ring improved inhibition, as demonstrated by **27** (K_I_ = 2693.3 nM), **28** (K_I_ = 2477.1 nM), **29** (K_I_ = 1954.5 nM), **30** (K_I_ = 1096.0 nM), **40** (K_I_ = 5175.6 nM), **41** (K_I_ = 2102.9 nM), **42** (K_I_ = 3067.5 nM) and **43** (K_I_ = 2724.3 nM). Moreover, the propanesulfonamide chain produced better inhibitors when it was coupled to the ether functional group. Considering molecules with urea moiety in the tail, the presence of halogens enhanced the affinity to hCA XII, as demonstrated by compounds **31** (K_I_ = 1504.3 nM) and **33** (K_I_ = 2631.6 nM), with a propanesulfonamide chain, and by **44** (K_I_ = 3713.9 nM) and **46** (K_I_ = 2625.0 nM), with a butanesulfonamide chain. These latter derivatives showed better inhibition than all the others belonging to the same type. The most effective compound against hCA XII was **50**, K_I_ of 1021.6 nM, with a phenyl ring as the R group. The analogue incorporating a F atom in *meta* position, **51**, showed a weaker inhibition, with a K_I_ of 3846.9 nM. Considering the corresponding derivatives with propanesulfonamide chain, the F atom (**38**) improved the potency against this isoform (K_I_ = 1059.4 nM), shows better K_I_ than **37**, K_I_ = 1360.9 nM. **52** was the less potent inhibitor (K_I_ in the high micromolar range, 7811.9 nM).

As for target/off-target CAs selectivity ratios of these aliphatic sulfonamide, most compounds exhibited good I/III and II/III selectivity ratios (*SI*), spaning in the ranges 0.99 − 18.29 and 0.89 − 26.20, respectively (Table 2). Despite the presence of bulky residues in the active sites of hCA I and hCA III and the aromatic sulfonamides difficulty to give a low nanomolar inhibition on both isozymes, aliphatic sulfonamide shows a good selectivity on hCA III compared to hCA I. Indeed, compounds **31**–**36**, **41**–**42**, **47**–**49** gave better *SI* values, reaching more than one order of magnitude selectivity (*SI* > 10). Particularly, **34** (*SI*: hCA I/hCA III, 18.29) has the greatest selectivity on hCA III over hCA I. In contrast, compound **37** shows a better inhibition on hCA I over hCA III with a *SI* value of 0.99. **27** (*SI*: hCA I/hCA III, 1.16), **38** (*SI*: hCA I/hCA III, 1.21) and **50** (*SI*: hCA I/CA III, 1.18) have a similar potency on hCA I and hCA III. As just described for hCA I/CA III, even for hCA II/hCA III only one compound (**36**) shows a better inhibition on hCA II than hCA III, (*SI*: hCA I/hCA III, 0.89). Besides that, **35** (*SI*: hCA I/hCA III, 1.19), **37** (*SI*: hCA I/hCA III, 1.05), **47** (*SI*: hCA I/hCA III, 1.15) and **50** (*SI*: hCA I/hCA III, 1.00) show no selectivity on hCA III over hCA II. Compounds **39**, **41, 43** and **52** exert higher selectivity on hCA III over hCA II, respectively 19.34, 13.14, 13.44 and 26.20. Moreover, **30** (*SI*: hCA I/hCA III, 11.89), **44** (*SI*: hCA I/hCA III, 12.66) and **49** (*SI*: hCA I/hCA III, 10.26) have a good affinity on hCA III over hCA II. All the *SI* values of the synthesised aliphatic sulfonamides result as better than the *SI* value of the standard and commercial hCAI **AAZ**.

### In silico study

The binding mode of the aliphatic sulfonamides **27–52** to hCA III, as the target, and hCA II, as the main off-target isoform, was studied *in silico* to find the relationship between their structural features and notable inhibition profiles.

It should be stressed again that, among the fifteen hCA isoforms, hCA III uniquely possesses a Phe198 in place of the highly conserved Leu198 (Figure 2), which hinders the binding of classical (hetero)aromatic sulfonamides. These derivatives bind the zinc(II) ion by the deprotonated primary sulfonamide moiety (SO_2_NH^-^) that replaces the metal-bound nucleophile according to a tetrahedral geometry. The sulfonamide moiety further engages two H-bonds with the side chain OH and backbone NH of T199, through the N-H and S = O groups, respectively. In addition, the aromatic ring plays a very important role in the stabilisation of the inhibitor binding mode: indeed, its particular orientation is due to a large network of vdW interactions with residues Q92, V121, F131, V143, L198, T200 and W209 (in hCA II, Figure S1, Supporting Information)^2^. The presence of F198 in hCA III is instead not compatible with the described binding mode and prevents an effective inhibition for aromatic sulfonamides against this isoform (Figure S1, Supporting Information).

**Figure 6.**
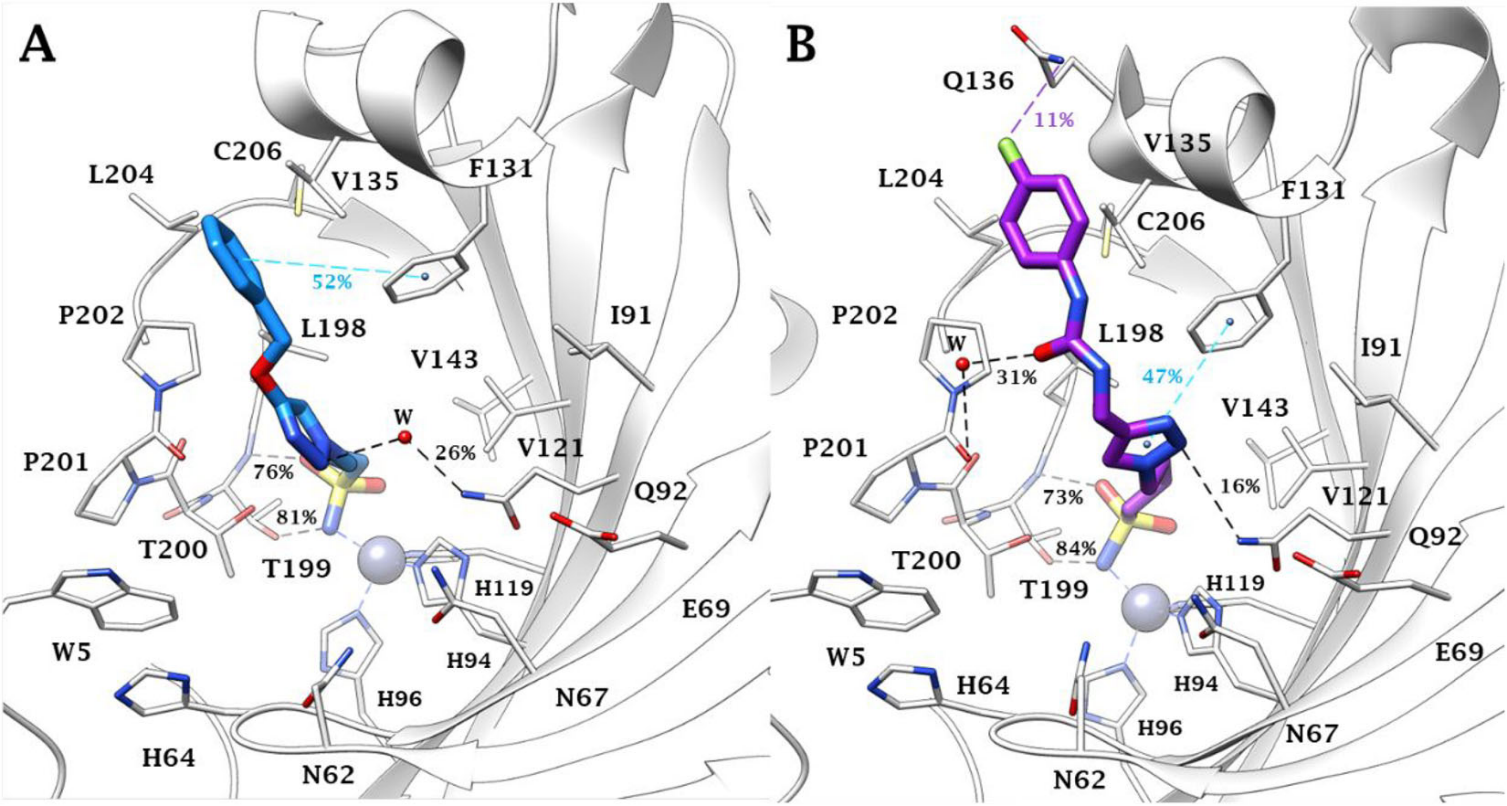
Binding mode of A) **27** (blue) and B) **44** (violet) into the hCA II active site: the main conformer throughout 100 ns of molecular dynamics is depicted. Water molecules are shown as red spheres, while H-bonds, weak H-bonds and π-π stacking interactions are represented as black, violet and cyan dashed lines, respectively. Bond occupancy over the MD simulation is indicated as percentage. The zinc coordination occupancy is 100%.

**Figure 7.**
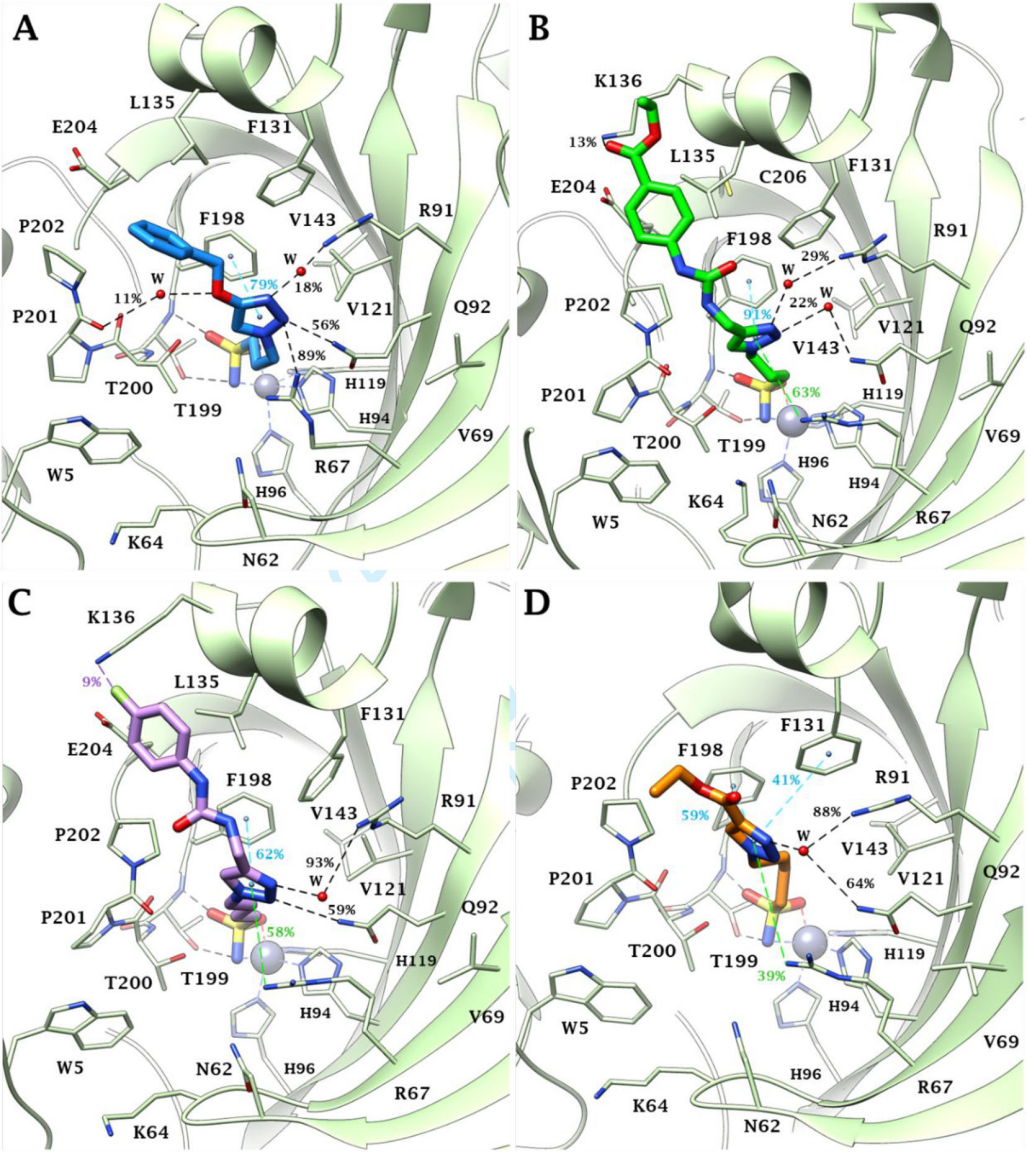
Binding mode of A) **27** (blue), B) **34** (green), C) **44** (violet) and B) **52** (orange) into hCA III active site: the main conformer throughout 100 ns of molecular dynamics is depicted. of Water molecules are shown as red spheres, while H-bonds, weak H-bonds, π-π stacking and π-cation interactions are represented as black, violet, cyan and green dashed lines, respectively. Bond occupancy over the MD simulation is indicated as percentage. The occupancy of the zinc coordination and H-bond to Thr199 is 100%.

Hence, the binding mode of aliphatic sulfonamides was here investigated for the first time by docking studies and implemented by molecular dynamics simulations (100 ns long) for compounds **27**, **34**, **44** and **52**, chosen as representative of all derivative types, to assess the stability of the binding poses. It was surprising that the ligand sulfonamide group binds the zinc ion in a tetracoordinate manner in hCA II (Figure 6), whilst it binds the metal ion according to a bipyramidal trigonal geometry in the hCA III active site (Figure 7). In both cases, the sulfonamide NH^-^ and the S = O (not involved in the zinc coordination in hCA III) groups are in H-bond distance with the side chain OH and backbone NH of T199, respectively.

**Figure 8.**
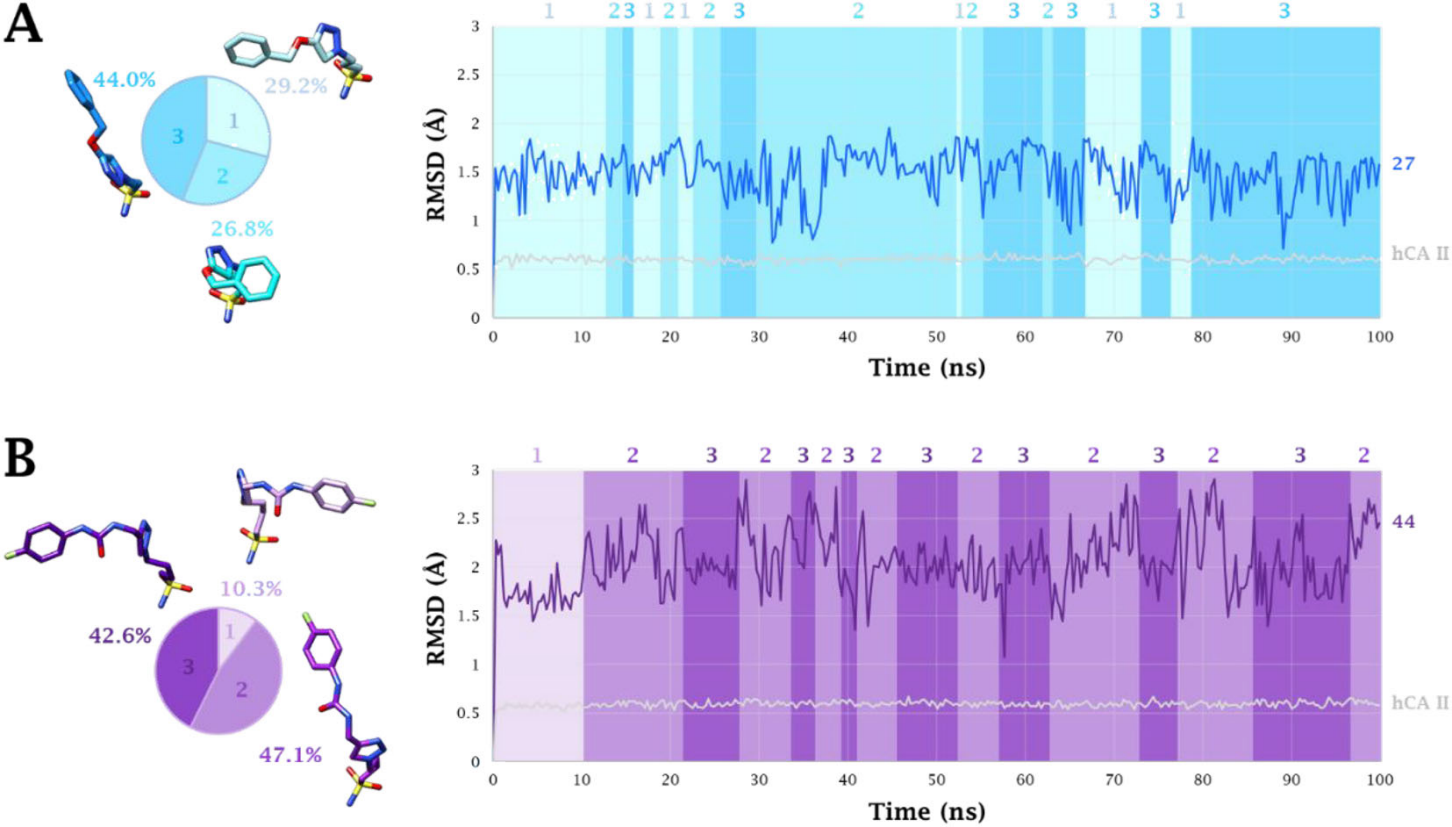
RMSD (Å) from 100 ns long MD simulations for A) **27**-hCA II and B) **44**-hCA II adducts. The representative conformers *per* cluster are depicted on the left together with the pie representation of their respective abundancies. The binding conformation assumed by ligands during MD simulation is indicated with the same colour code in the graph.

Conformers of both **27** and **44** cluster in three alternative binding modes during the MD simulations performed within the hCA II, with an RMSD value of about 1.49 Å and 2.04 Å, respectively (Figure 8). The second (compound **44**) and the third (compound **27**) clusters correspond to the ligand conformations that persist the most during dynamic simulations, (Figure 6). The inspection of the MD trajectories reveals that the aliphatic sulfonamides **27** and **44** are deeply bound to the zinc ion and that the tetrahedral metal coordination is maintained throughout the dynamics (persistence of 100%) within the hCA II active site. However, the absence in the ligands of an aromatic/heteroaromatic ring compared to classical CAIs destabilised the persistence of the two H-bonds with the side chain OH (76% and 73%) and backbone NH (81% and 84%) of T199 (Figure 6).

The length of the linker is strategic for the triazole ring interactions with Q92 and F131. Indeed, the shorter derivative **27** (*n* = 3, Figure 6(A)) undertook more long-lasting water-bridged H-bonds with the triazole N2 atom and the sidechain NH_2_ of Q92 than the longer **44** (*n* = 4, 26% vs 16% persistence). Instead, the triazole of compound **44** only was able to engage a π-π stacking interaction with the phenyl ring of the residue F131 (47%, Figure 6(B)).

The benzyl moiety of **27** lodges in a hydrophobic cleft establishing π-π interactions with the phenyl ring of F131 (52%, Figure 6(A)). Instead, within the same pocket, the interactions established by the 4-fluorophenyl moiety of **44** involve the weaker F^…^H_2_N (Q136, 11% persistence) and water-bridged C = O^…^HOH^…^O = C (P301, 31% persistence) H-bonds (Figure 6(B)).

**Figure 9.**
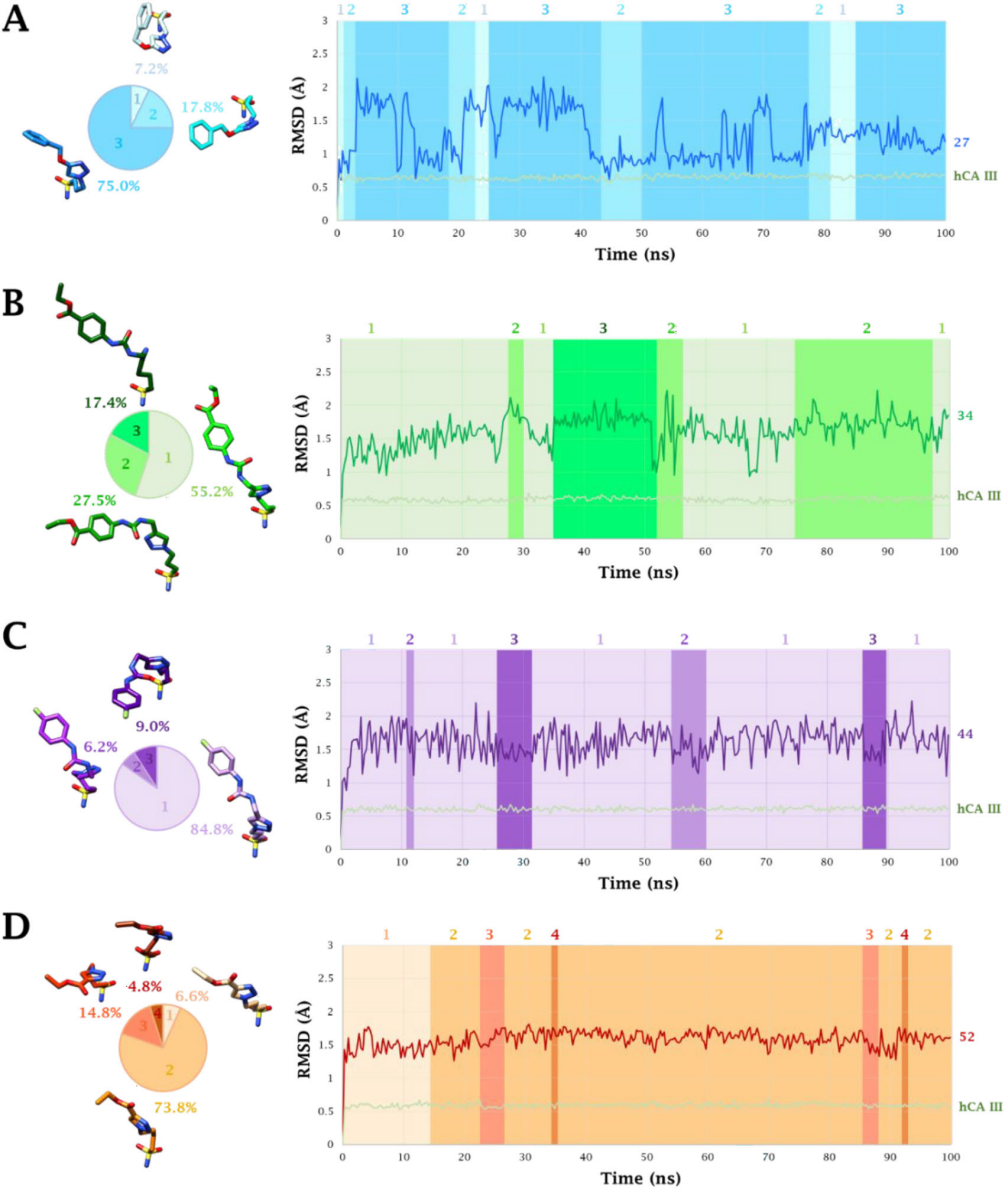
RMSD (Å) from 100 ns long MD simulations for A) **27**-hCA III, B) **34**-hCA III, C) **44**-hCA III and D) **52**-CA III adducts. The representative conformers per cluster are depicted on the left together with the pie representation of their respective abundancies. The binding conformation assumed by ligands during MD simulation is indicated with the same colour code in the graph.

In the case of hCA III the zinc atom is penta-coordinated during the entire 100 ns long MD simulation of **27**, **34**, **44** and **52**. Three and four clusters of conformers are formed for compounds **27**, **34**, **44** and compound **52**, respectively (Figures 7 and 9). In the zinc-coordination sphere residues H94, H96, and H119 occupy three binding sites around the metal ion while the other two sites are bound by the ligand-deprotonated sulfonamide moiety (NH^-^ and S = O). This group also strongly interacts with the backbone NH (100%) and sidechain OH (100%) of T199 and the binding within the active site is stabilised by a wide network of vdW interactions involving the aliphatic linker and the sidechain of F198 (Figure 7).

Although the steric hindrance of the scaffold bearing the sulfonamide moiety was significantly lightened in the class of derivatives herein reported, compared to classic aromatic sulfonamides, the presence of F198 in the hCA III active site still did not allow a typical tetrahedral coordination even for the aliphatic such derivatives **27–52**. Instead, only the pentacoordination described above was possible. In fact, the presence of an aliphatic scaffold, in place of an aromatic ring, to bear the zinc-binding group allowed to bypass the steric encumbrance of F198 with the flexible chain engaging a wide network of vdW contacts with it to stabilise the ligand-target interaction. The last evidence is pointed out by the higher RMSD of compounds **27** and **44** within the hCA II active site than that in complex with hCA III (**27**: 1.49 Å *vs* 1.28 Å for; **44**: 2.04 Å *vs* 1.61 Å; Figures 8 and 9).

Indeed, all the ligands **27**, **34, 44** and **52** are inclined to fold, independently by the linker length (*n* = 3 or 4), to improve the hydrophobic contacts with the target allowing the triazole ring to engage a π-π stacking interaction with F198 (79%, 91%, 62% and 59%, respectively), water-bridged H-bonds with the sidechain of R91 (18%, 29%, 93%, 88%, correspondingly) and Q92 (56%, 22%, 59%, 64%, ono-to-one), and undertake a plethora of interactions with the sidechain ammonium group of R67 (89%, 63%, 58%, 39%, in that order), such as direct H-bond, water-bridged H-bond and π-cation interactions (Figure 7). Only the triazole ring of derivatives **52** engaged a second π-π stacking interaction with the phenyl ring of residue F131 (41%).

Generally, the substituent in position 4 on the triazole ring took place in the cleft lined by P202, E204, C206, L135, F131 and K136, prevalently interacting with hydrophobic contacts. In particular, ligand **27** engaged a non-very persistent water-bridged H-bond between the ether O atom and the backbone C = O of P201 (11%, Figure 7(A)), derivatives **34** and **44** rarely achieve an H-bond distance with the sidechain NH_3_^+^ of K136 (13% and 9%, respectively Figures 7(B,C)), while the shorter pendant of compound **52** is oriented towards the exit of the active site (Figure 7(D)).

## Conclusions

hCA III is a rather neglected isoform expressed in a number of tissues, such as skeletal muscle, adipose tissue, liver and brain. Compared to other isoforms, such as hCA II and VII, hCA III shows a low CO_2_ hydrase activity, around 1% that of hCA II. This is probably due to two particular amino acid substitutions in the hCA III active site, which are His64/Lys64 and Leu198/Phe198, as compoared to other cytosolic CAs. During the last decades, several hypotheses were made over the physio-pathological role of hCA III in oxidative stress, obesity and several malignancies, but its functions remain largely uncertain. In order to investigate the role of this enzyme in several pathologic or physiologic processes, it is crucial to have effective and isoform-selective hCA III inhibitors, which do not exist at the moment, because, according to our drug design strategy, the Leu198/Phe198 mutation prevents the efficient binding of aromatic/heterocyclic sulfonamides and other classes of inhibitors. Here, we reported for the first time the design and synthesis of a new class of aliphatic sulfonamides bypassing the issues of classical hCAIs in selectively hitting hCA III. The synthesised compounds **27**–**52** were tested against a panel of hCA isoforms, namely hCA I, II, III, IV, VA and VB, VII and XII, showing a marked selectivity for hCA III. Compounds **39** and **52** were the most potent and selective hCA III inhibitors, with K_I_ values of 162.6 nM and 310.4 nM, respectively, with the latter being also the most selective one with a hCA III/hCA II *SI* of 26.2. Docking and molecular dynamics simulations were used to investigate the ligand/target interaction mode of this new class of hCAIs, which will represent valuable tools to improve our understanding of the physio-pathological role of hCA III.

## Experimental section

### Chemistry

Anhydrous solvents and all reagents were purchased from Merck, Fluorochem, and TCI. All reactions involving air- or moisture-sensitive compounds were performed under a nitrogen atmosphere using dried glassware and syringe techniques to transfer solutions. Nuclear magnetic resonance (^1^H-NMR, ^13^C-NMR) spectra were recorded using a Bruker Advance III 400 MHz spectrometer in DMSO-d_6_. Chemical shifts are reported in parts per million (ppm), and the coupling constants (J) are expressed in Hertz (Hz). Splitting patterns are designated as follows: s, singlet; d, doublet; t, triplet; q, quadruplet; m, multiplet; dd, doublet of doublets. The assignment of exchangeable protons was confirmed by the addition of D_2_O. Analytical thin-layer chromatography (TLC) was carried out on Sigma-Aldrich silica gel F-254 plates. Flash chromatography purifications were performed on Sigma-Aldrich Silica gel 60 (230–400 mesh ASTM) as the stationary phase, and ethyl acetate/n-hexane or MeOH/DCM were used as eluents. Melting points (mp) were measured in open capillary tubes with a Gallenkamp MPD350.BM3.5 apparatus and were uncorrected. HRMS analyses were performed on a Bruker Daltonics MicrOTOF-Q II mass spectrometer. All spectra were in accord with the assigned structures. All final compounds were >96% pure. The purity of target compounds was assessed by HPLC using an Agilent 1200 Series gradient HPLC system with a Luna PFP column (3 μm, 2 mm x 30 mm) at a flow rate of 0.25 ml/min and a linear gradient of the mobile phase – *i.e.* 10 mM formic acid and 5 mM ammonium formate in mQ water solution (solvent A) and 10 mM formic acid and 5 mM ammonium formate in methanol (solvent B). HPLC traces are reported in the Supporting Information.

### General synthetic procedure of sodium 3-Azidopropane-1-sulfonate (3) and sodium 4-azidobutane-1-sulfonate (4)

A solution of NaN_3_ (1 equiv) in H_2_O (4 ml) was added dropwise at rt to a stirred solution of an appropriate sultone **1** or **2** (1 g, 1 equiv) in acetone (20 ml) and the resulting mixture was stirred for about 4 h at rt. Solvent was removed under vacuum and the resulting sodium salt was suspended in Et_2_O and filtered to obtain a white powder in high yield.

#### Sodium 3-azidopropane-1-sulfonate (3)

Compound **3** was obtained according to the general procedure earlier reported with *1,3-propanesultone* (**1**) as starting material. Yield 98%; m.p. 163–166 °C; silica gel TLC R*_f_* 0.01 (MeOH/DCM 10% *v/v*); δ_H_ (400 MHz, DMSO-d_6_): 3.40 (t, *J* = 6.2 Hz, 2H, CH_2_), 2.45 (t, *J* = 6.9 Hz, 2H, CH_2_), 1.81 (m, 2H, CH_2_); δ_C_ (400 MHz, DMSO-d_6_): 49.3, 48.0, 23.0.

#### Sodium 4-azidobutane-1-sulfonate (4)

Compound **4** was obtained according to the general procedure earlier reported with *1,4-butanesultone* (**2**) as starting material. Yield 74%; m.p. 187–189 °C; silica gel TLC R*_f_* 0.01 (MeOH/DCM 10% *v/v*); δ_H_ (400 MHz, DMSO-d_6_): 3.33 (t, *J* = 6.4 Hz, 2H, CH_2_), 2.44 (t, *J* = 6.7 Hz, 2H, CH_2_), 1.27 (m, 4H, 2 x CH_2_); δ_C_ (400 MHz, DMSO-d_6_): 51.9, 47.0, 24.1, 20.2.

### *General synthetic procedure of 5*–*6*

SOCl_2_ (5 equiv) was added dropwise at 0 °C to appropriate sulfonate **3** or **4** (0.5 g, 1 equiv) under nitrogen atmosphere, then the suspension was heated o.n. at 60 °C. The solvent was removed under nitrogen and the obtained powder was solubilised in DCM (50 ml). The organic phase was washed with a NaHCO_3(s)_ (3 × 25 ml) and it was evaporated under vacuum to obtain an oil. NH_4_OH 28–30% (2 equiv) was added dropwise at 0 °C to a solution of the earlier obtained sulfonyl chloride (1 equiv) in dry THF under a nitrogen atmosphere. The solvent was removed under vacuum and acetone (30 ml) was added to give a suspension, that was filtered off. The organic phase was evaporated to obtain the desired product as a light yellow powder **5** or an uncoloured oil **6**.

#### 3-Azidopropane-1-sulfonamide (5)

Compound **5** was obtained according to the general procedure earlier reported with **3** as starting material. Yield 58%; m.p. 159–161 °C; silica gel TLC R*_f_* 0.62 (MeOH/DCM 10% *v/v*); δ_H_ (400 MHz, DMSO-d_6_): 6.90 (s, 2H, exchange with D_2_O, SO_2_NH_2_), 3.50 (d, *J* = 6.2 Hz, 2H, CH_2_), 3.05 (d, *J* = 6.2 Hz, 2H, CH_2_), 1.95 (q, *J* = 6.2 Hz, 2H, CH_2_); δ_C_ (400 MHz, DMSO-d_6_): 59.7, 48.0, 19.2.

#### 3-Azidobutane-1-sulfonamide (6)

Compound **6** was obtained according to the general procedure earlier reported with **4** as starting material. Yield 67%; m.p. 175–178 °C; silica gel TLC R*_f_* 0.60 (MeOH/DCM 10% *v/v*); δ_H_ (400 MHz, DMSO-d_6_): 6.83 (s, 2H, exchange with D_2_O, SO_2_NH_2_), 3.01 (d, *J* = 6.2 Hz, 2H, CH_2_), 1.74 (d, *J* = 6.2 Hz, 2H, CH_2_), 1.65 (m, 4H, 2 x CH_2_); δ_C_ (400 MHz, DMSO-d_6_): 62.3, 50.0, 28.4, 16.2.

### *General synthetic procedure of ethers 11*–*14*

K_2_CO_3_ (1.2 equiv) and propargyl bromide (1.2 equiv) were added to a stirred solution of appropriate phenol **7**–**10** (1 g, 1 equiv) in dry DMF (2 ml) under nitrogen atmosphere and the reaction mixture was heated at 60 °C for about 6 h. The suspension was cooled and quenched with slush and NaOH 5 M until pH = 10. The resulting precipitate was filtered and washed with H_2_O and Et_2_O to give a white powder.

#### (Prop-2-yn-1-yloxy)benzene (11)

Compound **11** was obtained according to the general procedure earlier reported with *phenol* (7) as starting material. Yield 96%; m.p. 273 °C; silica gel TLC R*_f_* 0.9 (MeOH/DCM 10% *v/v*); δ_H_ (400 MHz, DMSO-d_6_): 7.33 (t, *J* = 8.1 Hz, 2H, Ar-H), 7.00 (m, 3H, Ar-H), 4.81 (d, *J* = 2.1 Hz, 2H, CH_2_), 3.59 (t, *J* = 2.1 Hz, 1H, C*H*); δ_C_ (400 MHz, DMSO-d_6_): 156.2, 128.9, 121.4, 114.8, 79.2, 78.7, 55.3.

#### 1-Fluoro-4-(prop-2-yn-1-yloxy)benzene (12)

Compound **12** was obtained according to the general procedure earlier reported with *4-fluorophenol* (8) as starting material. Yield 85%; m.p. 280 °C; silica gel TLC R*_f_* 0.87 (MeOH/DCM 10% *v/v*); δ_H_ (400 MHz, DMSO-d_6_): 6.97 (m, 4H, Ar-H), 4.66 (d, *J* = 2.5 Hz, 2H, CH_2_), 2.52 (t, *J* = 2.5 Hz, 1H, C*H*); δ_C_ (400 MHz, DMSO-d_6_): 157.8 (d, *J* = 238.0 Hz), 153.6 (d, *J* = 2.0 Hz), 116.2 (d, *J* = 8.0 Hz), 115.9 (d, *J* = 23.0 Hz), 78.2, 75.6, 56.5.

#### 1-Methoxy-4-(prop-2-yn-1-yloxy)benzene (13)

Compound **13** was obtained according to the general procedure earlier reported with *4-methoxyphenol* (9) as starting material. Yield 89%; m.p. 143–146 °C; silica gel TLC R*_f_* 0.85 (MeOH/DCM 10% *v/v*); δ_H_ (400 MHz, DMSO-d_6_): 6.93 (d, *J* = 9.0 Hz, 2H, Ar-H), 6.85 (d, *J* = 9.0 Hz, 2H, Ar-H), 4.64 (d, *J* = 2.5 Hz, 2H, CH_2_), 3.78 (s, 3H, CH_3_), 2.51 (t, *J* = 2.5 Hz, 1H, C*H*); δ_C_ (400 MHz, DMSO-d_6_): 154.5, 151.6, 116.1, 114.6, 78.9, 75.3, 56.6, 55.7.

#### 1-Methoxy-3-(prop-2-yn-1-yloxy)benzene (14)

Compound **14** was obtained according to the general procedure earlier reported with *3-methoxyphenol* (10) as starting material. Yield 62%; m.p. 239 °C; silica gel TLC R*_f_* 0.72 (MeOH/DCM 8% *v/v*); δ_H_ (400 MHz, CDCl_3_-d_3_): 7.20 (m, 1H, Ar-H), 6.59 (m, 3H, Ar-H), 4.68 (d, *J* = 2.6 Hz, 2H, CH_2_), 3.80 (s, 3 H, CH_3_), 2.53 (t, *J* = 2.6 Hz, 1H, C*H*); δ_C_ (400 MHz, CDCl_3_-d_3_): 160.5, 158.5, 129.6, 106.9, 106.6, 101.2, 78.2, 75.2, 55.5, 55.0.

### *General synthetic procedure of ureas 21*–*26*

Propargyl amine (1.5 equiv) and DIPEA (cat) were added to a solution of appropriate isocyanate **15**–**20** (1 g, 1 equiv) in dry ACN (10 ml) under a nitrogen atmosphere and the reaction mixture was stirred at rt for about 3 h. The suspension was quenched with slush and HCl 1 M and the resulting precipitate was filtered and washed with H_2_O and Et_2_O to give a white powder with high yield and purity.

#### 1–(4-Fluorophenyl)-3-(prop-2-yn-1-yl)urea (21)

Compound **21** was obtained according to the general procedure earlier reported with *4-fuorobenzeneisocyanate* (15) as starting material. Yield 98%; m.p. 215–216 °C; silica gel TLC R*_f_* 0.49 (MeOH/DCM 8% *v/v*); δ_H_ (400 MHz, DMSO-d_6_): 8.67 (s, 1H, exchange with D_2_O, NHCONH), 7.42 (m, 2H, Ar-H), 7.09 (t, *J* = 9.3 Hz, 2H, Ar-H), 6.50 (t, *J* = 5.9 Hz, 1H, exchange with D_2_O, NHCONH), 3.89 (dd, *J* = 5.9 2.0 Hz, 2H, CH_2_), 3.13 (t, *J* = 2.0 Hz, 1H, C*H*); δ_C_ (400 MHz, DMSO-d_6_): 160.2 (d, *J* = 241.0 Hz), 156.8, 135.7, 117.1, 115.9 (d, *J* = 21.0 Hz), 80.1, 73.2, 32.0.

#### 1-(Prop-2-yn-1-yl)-3-(p-tolyl)urea (22)

Compound **22** was obtained according to the general procedure earlier reported with *4-tolylisocyanate* (16) as starting material. Yield 89%; m.p. 202–205 °C; silica gel TLC R*_f_* 0.38 (EtOAc/Hexane 50% *v/v*); δ_H_ (400 MHz, DMSO-d_6_): 8.48 (s, 1H, exchange with D_2_O, NHCONH), 7.28 (d, J = 8.2 Hz, 2H, Ar-H), 7.05 (d, J = 82 Hz, 2H, Ar-H), 6.41 (s, 1H, exchange with D_2_O, NHCONH), 3.88 (s, 2H, CH_2_), 3.11 (s, 1H, C*H*); δ_C_ (400 MHz, DMSO-d_6_): 162.9, 154.3, 135.0, 119.3, 115.4, 80.0, 72.9, 32.1.

#### 1-(Prop-2-yn-1-yl)-3–(4-(trifluoromethyl)phenyl)urea (23)

Compound **23** was obtained according to the general procedure earlier reported with *4-trifluorobenzeneisocyanate* (17) as starting material. Yield 91%; m.p. 193–195 °C; silica gel TLC R*_f_* 0.36 (EtOAc/Hexane 50% *v/v*); δ_H_ (400 MHz, DMSO-d_6_): 9.07 (s, 1H, exchange with D_2_O, NHCONH), 7.61 (m, 4H, Ar-H), 6.67 (t, *J* = 5.9 Hz, 1H, exchange with D_2_O, NHCONH), 3.91 (dd, *J* = 5.9 2.8 Hz, 2H, CH_2_), 3.15 (t, *J* = 2.9 Hz, 1H, C*H*); δ_C_ (400 MHz, DMSO-d_6_): 162.3, 142.7, 132.1 (q, J = 269.4 Hz), 125.3, 124.1 (q, J = 26.7 Hz), 121.9, 81.0, 73.8, 32.3.

#### Ethyl 4–(3-(prop-2-yn-1-yl)ureido)benzoate (24)

Compound **24** was obtained according to the general procedure earlier reported with *ethyl 4-isocyanatobenzoate* (18) as starting material. Yield 96%; m.p. 181–184 °C; silica gel TLC R*_f_* 0.41 (MeOH/DCM 10% *v/v*); δ_H_ (400 MHz, DMSO-d_6_): 9.06 (s, 1H, exchange with D_2_O, NHCONH), 7.86 (d, *J* = 9.2 Hz, 2H, Ar-H), 7.54 (d, *J* = 9.2 Hz, 2H, Ar-H), 6.76 (t, *J* = 6.1 Hz, 1H, exchange with D_2_O, NHCONH), 4.29 (q, *J* = 6.9 Hz, 2H, CH_2_), 3.92 (dd, *J* = 6.1 2.4 Hz, 2H, CH_2_), 3.14 (t, *J* = 2.4 Hz, 1H, C*H*), 1.32 (t, *J* = 2.4 Hz, 3H, CH_3_); δ_C_ (400 MHz, DMSO-d_6_): 165.9, 154.3, 143.7, 130.1, 125.7, 121.5, 80.2, 73.1, 32.2, 14.1.

#### 1–(3-Methoxyphenyl)-3-(prop-2-yn-1-yl)urea (25)

Compound **25** was obtained according to the general procedure earlier reported with *3-methoxybenzeneisocyanate* (19) as starting material. Yield 79%; m.p. 147–149 °C; silica gel TLC R*_f_* 0.29 (EtOAc/Hexane 50% *v/v*); δ_H_ (400 MHz, DMSO-d_6_): 8.08 (d, *J* = 7.4 Hz, 1H, Ar-H), 8.00 (s, 1H, exchange with D_2_O, NHCONH), 7.17 (t, *J* = 9.1 Hz, 1H, exchange with D_2_O, NHCONH), 6.91 (t, *J* = 7.4 Hz, 1H, Ar-H), 6.90 (d, J *=* 7.4 Hz, 1H, Ar-H), 6.85 (t, *J* = 7.4 Hz, 1H, Ar-H), 3.89 (m, 2H, CH_2_), 3.74 (s, 3H, CH_3_), 3.13 (s, 1H, C*H*); δ_C_ (400 MHz, DMSO-d_6_): 155.2, 148.0, 129.6, 121.8, 121.0, 118.7, 111.2, 82.4, 73.4, 56.2, 9.1.

#### 1–(4-Methoxy-2-methylphenyl)-3-(prop-2-yn-1-yl)urea (26)

Compound **26** was obtained according to the general procedure earlier reported with *2-methyl-4-methoxybenzeneisocyanate* (20) as starting material. Yield 88%; m.p. 231–234 °C; silica gel TLC R*_f_* 0.41 (MeOH/DCM 10% *v/v*); δ_H_ (400 MHz, DMSO-d_6_): 7.63 (s, 1H, exchange with D_2_O, NHCONH), 7.46 (d, *J* = 8.7 Hz, 1H, Ar-H), 6.76 (d, *J* = 2.8 Hz, 1H, Ar-H), 6.71 (dd. J = 8.7 2.8 Hz, 1H, Ar-H), 6.59 (t, *J* = 5.6 Hz, 1H, exchange with D_2_O, NHCONH), 3.88 (dd, *J* = 5.6 2.0 Hz, 2H, CH_2_), 3.13 (t, *J* = 2.0 Hz, 1H, C*H*); δ_C_ (400 MHz, DMSO-d_6_): 156.1, 152.8, 135.3, 120.8, 111.9, 80.3, 73.3, 55.8, 32.1, 17.2.

### *General synthetic procedure of 27*–*52*

Cu(CH_3_COO)_2_ (0.1 equiv) and sodium ascorbate (1 equiv) were added to a stirred solution of appropriate azides **5** or **6** (0.15 g, 1 equiv) and alkynes **11**–**14**, **21**–**26,** ethyl propiolate, ethynylbenzene and 1-ethynyl-3-fluorobenzene (1 equiv) in MeOH/THF (1/3 ml) and the reaction mixture was heated o.n. at 40 °C. The solvent was evaporated under vacuum, slush was added and products were extracted in EtOAc (3 × 25 ml). The organic phase was dried with Na_2_SO_4_, filtered and evaporated to give products **27**–**52**. All the compounds were purified by silica gel chromatography (EtOAc/Hexane, 20% to 50%).

#### 3–(4-(Phenoxymethyl)-1H-1,2,3-triazol-1-yl)propane-1-sulfonamide (27)

Compound **27** was obtained according to the general procedure earlier reported with **5** and **11** as starting materials. Yield 30%; m.p. 254–255 °C; silica gel TLC R*_f_* 0.22 (MeOH/DCM 10% *v/v*); δ_H_ (400 MHz, DMSO-d_6_): 8.29 (s, 1H, Ar-H), 7.33 (t, *J* = 7.6 Hz, 2H, Ar-H), 7.06 (d, *J* = 7.6 H, 2H, Ar-H), 6.98 (t, *J* = 7.6 Hz, 1H, Ar-H), 6.92 (s, 2H, exchange with D_2_O, SO_2_NH_2_), 5.16 (s, 2H, CH_2_), 4.54 (t, *J* = 6.6 Hz, 2H, CH_2_), 3.01 (t, *J* = 6.6 Hz, 2H, CH_2_), 2.26 (m. 2H, CH_2_); δ_C_ (400 MHz, DMSO-d_6_): 159.1, 143.9, 130.5, 125.6, 121.9, 115.7, 62.0, 52.6, 48.8, 25.9; ESI-HRMS (m/z) [M-H]^-^: calculated for C12H15N4O3S 295.0870; found 295.0875.

#### 3–(4-((4-Fluorophenoxy)methyl)-1H-1,2,3-triazol-1-yl)propane-1-sulfonamide (28)

Compound **28** was obtained according to the general procedure earlier reported with **5** and **12** as starting materials. Yield 68%; m.p. 261–263 °C; silica gel TLC R*_f_* 0.18 (MeOH/DCM 10% *v/v*); δ_H_ (400 MHz, DMSO-d_6_): 8.28 (s, 1H, Ar-H), 7.16 (t, *J* = 8.5 Hz, 2H, Ar-H), 7.08 (m, 2H, Ar-H), 6.92 (s, 2H, exchange with D_2_O, SO_2_NH_2_), 5.14 (s, 2H, CH_2_), 4.54 (t, *J* = 6.8 Hz, 2H, CH_2_), 3.00 (t, *J* = 6.8 Hz, 2H, CH_2_), 2.26 (m. 2H, CH_2_); δ_C_ (400 MHz, DMSO-d_6_): 158.9 (d, *J* = 239.6), 155.4, 143.7, 125.6, 117.1 (d, *J* = 8.3 Hz), 117.0 (d, *J* = 23.9), 62.7, 52.6, 48.8, 25.9. ESI-HRMS (m/z) [M-H]^-^: calculated for C12H14FN4O3S 313.0776; found 313.0781.

#### 3–(4-((4-Methoxyphenoxy)methyl)-1H-1,2,3-triazol-1-yl)propane-1-sulfonamide (29)

Compound **29** was obtained according to the general procedure earlier reported with **5** and **13** as starting materials. Yield 67%; m.p. 273–274 °C; silica gel TLC R*_f_* 0.21 (MeOH/DCM 10% *v/v*); δ_H_ (400 MHz, DMSO-d_6_): 8.25 (s, 1H, Ar-H), 6.99 (d, *J* = 8.5 Hz, 2H, Ar-H), 6.90 (s, 2H, exchange with D_2_O, SO_2_NH_2_), 6.88 (d, *J* = 8.5 Hz, 2H, Ar-H), 5.09 (s, 2H, CH_2_), 4.63 (t, *J* = 6.7 Hz, 2H, CH_2_), 3.70 (s, 3H, CH_3_), 3.00 (t, *J* = 6.7 Hz, 2H, CH_2_), 2.26 (m. 2H, CH_2_); δ_C_ (400 MHz, DMSO-d_6_): 154.6, 153.1, 144.1, 125.5, 116.7, 115.6, 62.7, 56.4, 52.6, 48.8, 25.9. ESI-HRMS (m/z) [M-H]^-^: calculated for C13H17N4O4S 325.0976; found 325.0982.

#### 3–(4-((3-Methoxyphenoxy)methyl)-1H-1,2,3-triazol-1-yl)propane-1-sulfonamide (30)

Compound **30** was obtained according to the general procedure earlier reported with **5** and **14** as starting materials. Yield 39%; m.p. 270–272 °C; silica gel TLC R*_f_* 0.12 (MeOH/DCM 10% *v/v*); δ_H_ (400 MHz, DMSO-d_6_): 8.28 (s, 1H, Ar-H), 7.22 (t, *J* = 8.2 Hz, 1H, Ar-H), 6.92 (s, 2H, exchange with D_2_O, SO_2_NH_2_), 6.64 (m, 2H, Ar-H), 6.56 (d, *J* = 8.2 Hz, 2H, Ar-H), 5.14 (s, 2H, CH_2_), 4.54 (t, *J* = 6.9 Hz, 2H, CH_2_), 3.75 (s, 3H, CH_3_), 3.00 (t, *J* = 6.9 Hz, 2H, CH_2_), 2.26 (m, 2H, CH_2_); δ_C_ (400 MHz, DMSO-d_6_): 161.5, 160.3, 143.8, 131.0, 125.6, 107.9, 107.6, 102.0, 62.1, 56.1, 52.6, 48.8, 25.9. ESI-HRMS (m/z) [M-H]^-^: calculated for C13H17N4O4S 325.0976; found 325.0981.

#### 3–(4-((3–(4-Fluorophenyl)ureido)methyl)-1H-1,2,3-triazol-1-yl)propane-1-sulfonamide (31)

Compound **31** was obtained according to the general procedure earlier reported with **5** and **21** as starting materials. Yield 43%; m.p. 249–252 °C; silica gel TLC R*_f_* 0.19 (MeOH/DCM 10% *v/v*); δ_H_ (400 MHz, DMSO-d_6_): 8.62 (s, 1H, exchange with D_2_O, NHCONH), 8.00 (s, 1H, Ar-H), 7.42 (m, 2H, Ar-H), 7.08 (t, *J* = 8.7 Hz, 2H, Ar-H), 6.90 (s, 2H, exchange with D_2_O, SO_2_NH_2_), 6.60 (t, *J* = 5.2 Hz, 1H, exchange with D_2_O, NHCONH), 4.50 (t, *J* = 6.6 Hz, 2H, CH_2_), 4.34 (d, *J* = 5.2 Hz, 2H, CH_2_), 2.99 (t, *J* = 6.6 Hz, 2H, CH_2_), 2.24 (m, 2H, CH_2_); δ_C_ (400 MHz, DMSO-d_6_): 160.9, 159.2 (d, *J* = 238.9 Hz), 156.1, 137.7, 124.0, 120.4 (d, *J* = 7.98 Hz), 116.3 (d, *J* = 21.1), 52.8, 48.8, 36.0, 26.0. ESI-HRMS (m/z) [M-H]^-^: calculated for C13H16FN6O3S 355.0994; found 355.0999.

#### 3–(4-((3-(p-Tolyl)ureido)methyl)-1H-1,2,3-triazol-1-yl)propane-1-sulfonamide (32)

Compound **32** was obtained according to the general procedure earlier reported with **5** and **22** as starting materials. Yield 43%; m.p. 217–218 °C; silica gel TLC R*_f_* 0.28 (MeOH/DCM 10% *v/v*); δ_H_ (400 MHz, DMSO-d_6_): 8.46 (s, 1H, exchange with D_2_O, NHCONH), 8.01 (s, 1H, Ar-H), 7.29 (d, J = 8.9 Hz, 2H, Ar-H), 7.04 (d, *J* = 8.9 Hz, 2H, Ar-H), 6.91 (s, 2H, exchange with D_2_O, SO_2_NH_2_), 6.55 (t, *J* = 5.1 Hz, 1H, exchange with D_2_O, NHCONH), 4.50 (t, *J* = 6.9 Hz, 2H, CH_2_), 4.35 (d, *J* = 5.1 Hz, 2H, CH_2_), 2.99 (t, *J* = 6.9 Hz, 2H, CH_2_), 2.23 (m, 5H, CH_3_ + CH_2_); δ_C_ (400 MHz, DMSO-d_6_): 159.6, 156.4, 139.1, 131.0, 130.3, 123.9, 119.0, .52.7, 48.7, 36.0, 26.0, 21.5. ESI-HRMS (m/z) [M-H]^-^: calculated for C14H19N6O3S 351.1245; found 351.1251.

#### 3–(4-((3–(4-(Trifluoromethyl)phenyl)ureido)methyl)-1H-1,2,3-triazol-1-yl)propane-1-sulfonamide (33)

Compound **33** was obtained according to the general procedure earlier reported with **5** and **23** as starting materials. Yield 21%; m.p. >300 °C; silica gel TLC R*_f_* 0.18 (MeOH/DCM 10% *v/v*); δ_H_ (400 MHz, DMSO-d_6_): 9.18 (s, 1H, exchange with D_2_O, NHCONH), 8.02 (s, 1H, Ar-H), 7.61 (m, 4H, Ar-H), 6.93 (t, *J* = 5.6 Hz, 1H, exchange with D_2_O, NHCONH), 6.90 (s, 2H, exchange with D_2_O, SO_2_NH_2_), 4.51 (t, *J* = 6.4 Hz, 2H, CH_2_), 4.37 (d, *J* = 5.6 Hz, 2H, CH_2_), 2.99 (t, *J* = 6.4 Hz, 2H, CH_2_), 2.24 (m, 2H, CH_2_); δ_C_ (400 MHz, DMSO-d_6_): 155.9, 145.9, 130.2 (q, J = 306 Hz), 127.0, 123.8, 122.5 (q, J = 25.2 Hz), 121.9, 118.3, 52.6, 48.7, 35.9, 26.0. ESI-HRMS (m/z) [M-H]^-^: calculated for C14H16F3N6O3S 405.0962; found 405.0966.

#### Ethyl 4–(3-((1–(3-sulfamoylpropyl)-1H-1,2,3-triazol-4-yl)methyl)ureido)benzoate (34)

Compound **34** was obtained according to the general procedure earlier reported with **5** and **24** as starting materials. Yield 41%; m.p. 239–241 °C; silica gel TLC R*_f_* 0.38 (MeOH/DCM 10% *v/v*); δ_H_ (400 MHz, DMSO-d_6_): 9.06 (s, 1H, exchange with D_2_O, NHCONH), 8.03 (s, 1H, Ar-H), 7.86 (d, J = 8.2 Hz, 2H, Ar-H), 7.54 (d, *J* = 8.2 Hz, 2H, Ar-H), 6.91 (s, 2H, exchange with D_2_O, SO_2_NH_2_), 6.81 (t, *J* = 5.5 Hz, 1H, exchange with D_2_O, NHCONH), 4.50 (t, *J* = 6.8 Hz, 2H, CH_2_), 4.37 (d, *J* = 5.5 Hz, 2H, CH_2_), 4.28 (q, *J* = 6.8 Hz, 2H, CH_2_), 2.99 (t, *J* = 6.8 Hz, 2H, CH_2_), 2.24 (m, 2H, CH_2_), 1.32 (t, *J* = 6.8 Hz, 3H, CH_3_); δ_C_ (400 MHz, DMSO-d_6_): 165.2, 156.3, 139.1, 131.2, 130.2, 126.5, 119.1, 108.2, 55.0, 49.9, 36.0, 29.7, 22.1, 21.6. ESI-HRMS (m/z) [M-H]^-^: calculated for C16H21N6O5S 409.1300; found 409.1306.

#### 3–(4-((3–(3-Methoxyphenyl)ureido)methyl)-1H-1,2,3-triazol-1-yl)propane-1-sulfonamide (35)

Compound **35** was obtained according to the general procedure earlier reported with **5** and **25** as starting materials. Yield 30%; m.p. 214–215 °C; silica gel TLC R*_f_* 0.14 (MeOH/DCM 10% *v/v*); δ_H_ (400 MHz, DMSO-d_6_): 8.65 (s, 1H, exchange with D_2_O, NHCONH), 8.01 (s, 1H, Ar-H), 7.17 (s, 1H, Ar-H), 7.13 (t, *J* = 8.2 Hz, 1H, Ar-H), 6.90 (s, 2H, exchange with D_2_O, SO_2_NH_2_), 6.87 (d, *J* = 8.2 Hz, 1H, Ar-H), 6.65 (t, *J* = 5.1 Hz, 1H, exchange with D_2_O, NHCONH), 6.50 (d, *J* = 8.2 Hz, 1H, Ar-H), 4.50 (t, *J* = 6.7 Hz, 2H, CH_2_), 4.35 (d, *J* = 5.1 Hz, 2H, CH_2_), 3.72 (s, 3H, CH_3_), 2.99 (t, *J* = 6.7 Hz, 2H, CH_2_), 2.24 (m, 2H, CH_2_); δ_C_ (400 MHz, DMSO-d_6_): 160.8, 156.0, 142.7, 131.4, 130.5, 123.7, 111.3, 107.9, 104.6, 55.8, 52.8, 48.7, 35.9, 25.8. ESI-HRMS (m/z) [M-H]^-^: calculated for C14H19N6O4S 367.1194; found 367.1198.

#### 3–(4-((3–(4-Methoxy-2-methylphenyl)ureido)methyl)-1H-1,2,3-triazol-1-yl)propane-1-sulfonamide (36)

Compound **36** was obtained according to the general procedure earlier reported with **5** and **26** as starting materials. Yield 48%; m.p. 254–256 °C; silica gel TLC R*_f_* 0.09 (MeOH/DCM 10% *v/v*); δ_H_ (400 MHz, DMSO-d_6_): 8.00 (s, 1H, exchange with D_2_O, NHCONH), 7.62 (s, 1H, Ar-H), 7.53 (d, *J* = 8.2 Hz, 1H, Ar-H), 6.90 (s, 2H, exchange with D_2_O, SO_2_NH_2_), 6.76 (m, 1H, exchange with D_2_O, NHCONH, 1H, Ar-H), 6.70 (dd, *J* = 8.2 2.2 Hz, 1H, Ar-H), 4.51 (t, *J* = 6.9 Hz, 2H, CH_2_), 4.34 (d, *J* = 4.9 Hz, 2H, CH_2_), 3.72 (s, 3H, CH_3_), 3.00 (t, *J* = 6.9 Hz, 2H, CH_2_), 2.24 (m, 2H, CH_2_), 2.16 (s, 3H, CH_3_); δ_C_ (400 MHz, DMSO-d_6_): 168.1, 156.9, 156.1, 147.2, 132.7, 131.5, 124.7, 116.4, 112.2, 56.3, 52.7, 48.8, 36.1, 26.1, 19.3. ESI-HRMS (m/z) [M-H]^-^: calculated for C15H21N6O4S 381.1350; found 381.1356.

#### Ethyl 1–(3-Sulfamoylpropyl)-1H-1,2,3-triazole-4-carboxylate (37)

Compound **37** was obtained according to the general procedure earlier reported with **5** and ethyl propiolate as starting materials. Yield 96%; m.p. 163–165 °C; silica gel TLC R*_f_* 0.1 (MeOH/DCM 10% *v/v*); δ_H_ (400 MHz, DMSO-d_6_): 8.85 (s, 1H, Ar-H), 6.91 (s, 2H, exchange with D_2_O, SO_2_NH_2_), 4.59 (t, *J* = 6.5 Hz, 2H, CH_2_), 4.34 (q, *J* = 6.7 Hz, 2H, CH_2_), 3.00 (t, *J* = 6.5 Hz, 2H, CH_2_), 2.24 (m, 2H, CH_2_), 1.33 (t, *J* = 6.7 Hz, 3H, CH_3_); δ_C_ (400 MHz, DMSO-d_6_): 161.5, 140.0. 130.3, 61.7, 52.9, 49.2, 26.1, 15.1. ESI-HRMS (m/z) [M-H]^-^: calculated for C8H13N4O4S 261.0663; found 261.0668.

#### 3–(4-Phenyl-1H-1,2,3-triazol-1-yl)propane-1-sulfonamide (38)

Compound **38** was obtained according to the general procedure earlier reported with **5** and ethynylbenzene as starting materials. Yield 75%; m.p. 201–203 °C; silica gel TLC R*_f_* 0.31 (MeOH/DCM 10% *v/v*); δ_H_ (400 MHz, DMSO-d_6_): 8.63 (s, 1H, Ar-H), 7.87 (d, *J* = 8.1 Hz, 2H, Ar-H), 7.48 (t, *J* = 8.1 Hz, 2H, Ar-H), 7.37 (t, *J* = 8.1 Hz, 1H, Ar-H), 6.91 (s, 2H, exchange with D_2_O, SO_2_NH_2_), 4.58 (t, *J* = 6.8 Hz, 2H, CH_2_), 3.05 (t, *J* = 6.8 Hz, 2H, CH_2_), 2.31 (m, 2H, CH_2_); δ_C_ (400 MHz, DMSO-d_6_): 147.5, 131.9, 130.1, 129.0, 126.4, 122.6, 52.6, 49.0, 26.0. ESI-HRMS (m/z) [M-H]^-^: calculated for C11H13N4O2S 265.0765; found 265.0770.

#### 3–(4-(3-Fluorophenyl)-1H-1,2,3-triazol-1-yl)propane-1-sulfonamide (39)

Compound **39** was obtained according to the general procedure earlier reported with **5** and 1-ethynyl-3-fluorobenzene as starting materials. Yield 70%; m.p. 209–210 °C; silica gel TLC R*_f_* 0.21 (MeOH/DCM 10% *v/v*); δ_H_ (400 MHz, DMSO-d_6_): 8.71 (s, 1H, Ar-H), 7.73 (d, *J* = 8.0 Hz, 1H, Ar-H), 7.68 (dt, *J* = 10.0 2.3 Hz, 1H, Ar-H), 7.53 (q, *J* = 8.0 Hz, 1H, Ar-H), 7.20 (td, *J* = 8.0 2.3 Hz, 1H, Ar-H), 6.92 (s, 2H, exchange with D_2_O, SO_2_NH_2_), 4.59 (t, *J* = 6.9 Hz, 2H, CH_2_), 3.05 (t, *J* = 6.9 Hz, 2H, CH_2_), 2.31 (m, 2H, CH_2_); δ_C_ (400 MHz, DMSO-d_6_): 162 (d, *J* = 259 Hz), 146.6, 134.2 (d, *J* = 8.3 Hz,), 132.1 (d, *J* = 8.3 Hz), 123.3, 122.3, 115.7 (d, *J* = 19.6 Hz), 112.9 (d, *J* = 19.6 Hz), 52.8, 49.0, 26.1. ESI-HRMS (m/z) [M-H]^-^: calculated for C11H12FN4O2S 283.0670; found 283.0675.

#### 4–(4-(Phenoxymethyl)-1H-1,2,3-triazol-1-yl)butane-1-sulfonamide (40)

Compound **40** was obtained according to the general procedure earlier reported with **6** and **11** as starting materials. Yield 23%; m.p. 221–223 °C; silica gel TLC R*_f_* 0.25 (MeOH/DCM 10% *v/v*); δ_H_ (400 MHz, DMSO-d_6_): 8.27 (s, 1H, Ar-H), 7.32 (t. *J* = 7.5 Hz, 2H, Ar-H), 7.05 (d, *J* = 7.5 H, 2H, Ar-H), 6.97 (t, *J* = 7.5 Hz, 1H, Ar-H), 6.82 (s, 2H, exchange with D_2_O, SO_2_NH_2_), 5.15 (s, 2H, CH_2_), 4.43 (t, *J* = 6.8 Hz, 2H, CH_2_), 3.03 (t, *J* = 6.8 Hz, 2H, CH_2_), 1.97 (m. 2H, CH_2_), 1.68 (m. 2H, CH_2_); δ_C_ (400 MHz, DMSO-d_6_): 159.1, 143.8, 130.6, 125.5, 121.9, 115.7, 62.1, 54.8, 49.9, 29.6, 21.8. ESI-HRMS (m/z) [M-H]^-^: calculated for C13H17N4O3S 309.1027; found 309.1032.

#### 4–(4-((4-Fluorophenoxy)methyl)-1H-1,2,3-triazol-1-yl)butane-1-sulfonamide (41)

Compound **41** was obtained according to the general procedure earlier reported with **6** and **12** as starting materials. Yield 32%; m.p. 246–248 °C; silica gel TLC R*_f_* 0.16 (MeOH/DCM 10% *v/v*); δ_H_ (400 MHz, DMSO-d_6_): 8.25 (s, 1H, Ar-H), 7.16 (d, *J* = 8.9 H, 2H, Ar-H), 7.08 (m, 2H, Ar-H), 6.82 (s, 2H, exchange with D_2_O, SO_2_NH_2_), 5.13 (s, 2H, CH_2_), 4.42 (t, *J* = 6.7 Hz, 2H, CH_2_), 3.03 (t, *J* = 6.7 Hz, 2H, CH_2_), 1.97 (m. 2H, CH_2_), 1.68 (m. 2H, CH_2_); δ_C_ (400 MHz, DMSO-d_6_): 158.9 (d, *J* = 237.0 Hz), 155.4, 143.7, 125.5, 117.2 (d, *J* = 8.4 Hz), 117.0 (d, *J* = 23.5), 62.8, 54.7, 50.0, 29.3, 21.8. ESI-HRMS (m/z) [M-H]^-^: calculated for C13H16FN4O3S 327.0933; found 327.0938.

#### 4–(4-((4-Methoxyphenoxy)methyl)-1H-1,2,3-triazol-1-yl)butane-1-sulfonamide (42)

Compound **42** was obtained according to the general procedure earlier reported with **6** and **13** as starting materials. Yield 45%; m.p. 251–253 °C; silica gel TLC R*_f_* 0.25 (MeOH/DCM 10% *v/v*); δ_H_ (400 MHz, DMSO-d_6_): 8.23 (s, 1H, Ar-H), 6.99 (d, *J* = 8.3 H, 2H, Ar-H), 6.88 (d, *J* = 8.3 H, 2H, Ar-H), 6.81 (s, 2H, exchange with D_2_O, SO_2_NH_2_), 5.08 (s, 2H, CH_2_), 4.42 (t, *J* = 6.8 Hz, 2H, CH_2_), 3.72 (s, 3H, CH_3_), 3.03 (t, *J* = 6.8 Hz, 2H, CH_2_), 1.97 (m. 2H, CH_2_), 1.68 (m. 2H, CH_2_); δ_C_ (400 MHz, DMSO-d_6_): 154.7, 153.2, 144.3, 125.4, 116.9, 115.6, 62.7, 56.9, 54.8, 50.1, 29.7, 21.7. ESI-HRMS (m/z) [M-H]^-^: calculated for C14H19N4O4S 339.1132; found 339.1138.

#### 4–(4-((3-Methoxyphenoxy)methyl)-1H-1,2,3-triazol-1-yl)butane-1-sulfonamide (43)

Compound **43** was obtained according to the general procedure earlier reported with **6** and **14** as starting materials. Yield 29%; m.p. 186–188 °C; silica gel TLC R*_f_* 0.15 (MeOH/DCM 10% *v/v*); δ_H_ (400 MHz, DMSO-d_6_): 8.26 (s, 1H, Ar-H), 7.20 (t, *J* = 8.2 Hz, 1H, Ar-H), 6.82 (s, 2H, exchange with D_2_O, SO_2_NH_2_), 6.64 (d, *J* = 8.2 Hz, 1H, Ar-H), 6.59 (s, 1H, Ar-H), 6.56 (d, *J* = 8.2 Hz, 1H, Ar-H), 5.13 (s, 2H, CH_2_), 4.43 (t, *J* = 6.3 Hz, 2H, CH_2_), 3.75 (s, 3H, CH_3_), 3.03 (t, *J* = 6.3 Hz, 2H, CH_2_), 1.97 (m. 2H, CH_2_), 1.68 (m. 2H, CH_2_); δ_C_ (400 MHz, DMSO-d_6_): 161.6, 160.1, 142.3, 130.7, 128.6, 108.3, 106.6, 100.4, 72.3, 62.3, 55.8, 52.1, 16.6. ESI-HRMS (m/z) [M-H]^-^: calculated for C14H19N4O4S 339.1132; found 339.1138.

#### 4–(4-((3–(4-Fluorophenyl)ureido)methyl)-1H-1,2,3-triazol-1-yl)butane-1-sulfonamide (44)

Compound **44** was obtained according to the general procedure earlier reported with **6** and **21** as starting materials. Yield 20%; m.p. 284–286 °C; silica gel TLC R*_f_* 0.14 (MeOH/DCM 10% *v/v*); δ_H_ (400 MHz, DMSO-d_6_): 8.63 (s, 1H, exchange with D_2_O, NHCONH), 7.99 (s, 1H, Ar-H), 7.42 (m, 2H, Ar-H), 7.09 (t, *J* = 8.8 Hz, 2H, Ar-H), 6.80 (s, 2H, exchange with D_2_O, SO_2_NH_2_), 6.59 (t, *J* = 5.0 Hz, 1H, exchange with D_2_O, NHCONH), 4.39 (t, *J* = 6.2 Hz, 2H, CH_2_), 4.34 (d, *J* = 5.0 Hz, 2H CH_2_), 3.03 (t, *J* = 6.2 Hz, 2H, CH_2_), 1.95 (m. 2H, CH_2_), 1.68 (m. 2H, CH_2_); δ_C_ (400 MHz, DMSO-d_6_): 159.2 (d, *J* = 234.9 Hz), 156.2, 137.8, 136.1, 123.7, 120.4 (d, *J* = 7.8 Hz), 116.2 (d, *J* = 22.3 Hz), 55.0, 50.1, 36.0, 29.6, 22.2. ESI-HRMS (m/z) [M-H]^-^: calculated for C14H18FN6O3S 369.1151; found 369.1155.

#### 4–(4-((3-(p-Tolyl)ureido)methyl)-1H-1,2,3-triazol-1-yl)butane-1-sulfonamide (45)

Compound **45** was obtained according to the general procedure earlier reported with **6** and **22** as starting materials. Yield 44%; m.p. 271–272 °C; silica gel TLC R*_f_* 0.22 (MeOH/DCM 10% *v/v*); δ_H_ (400 MHz, DMSO-d_6_): 8.46 (s, 1H, exchange with D_2_O, NHCONH), 7.98 (s, 1H, Ar-H), 7.29 (d, *J* = 8.7 Hz, 2H, Ar-H), 7.05 (d, *J* = 8.7 Hz, 2H, Ar-H), 6.81 (s, 2H, exchange with D_2_O, SO_2_NH_2_), 6.54 (t, *J* = 5.2 Hz, 1H, exchange with D_2_O, NHCONH), 4.38 (t, *J* = 6.7 Hz, 2H, CH_2_), 4.33 (d, *J* = 5.2 Hz, 2H CH_2_), 3.02 (t, *J* = 6.7 Hz, 2H, CH_2_), 2.23 (s, 3H, CH_3_), 1.94 (m. 2H, CH_2_), 1.67 (m. 2H, CH_2_); δ_C_ (400 MHz, DMSO-d_6_): 171.4, 166.9, 155.7, 146.8, 131.4, 124.5, 118.3, 61.1, 52.5, 49.1, 36.3, 26.0, 15.3. ESI-HRMS (m/z) [M-H]^-^: calculated for C15H21N6O3S 365.1401; found 365.1407.

#### 4–(4-((3–(4-(Trifluoromethyl)phenyl)ureido)methyl)-1H-1,2,3-triazol-1-yl)butane-1-sulfonamide (46)

Compound **46** was obtained according to the general procedure earlier reported with **6** and **23** as starting materials. Yield 14%; m.p. >300 °C; silica gel TLC R*_f_* 0.11 (MeOH/DCM 10% *v/v*); δ_H_ (400 MHz, DMSO-d_6_): 9.75 (s, 1H, exchange with D_2_O, NHCONH), 8.02 (s, 1H, Ar-H), 7.64 (d, *J* = 8.2 Hz, 2H, Ar-H), 7.58 (d, *J* = 8.2 Hz, 2H, Ar-H), 7.18 (t, *J* = 5.5 Hz, 1H, exchange with D_2_O, NHCONH), 6.81 (s, 2H, exchange with D_2_O, SO_2_NH_2_), 4.37 (m, 4H, 2 x CH_2_), 3.02 (t, *J* = 6.9 Hz, 2H, CH_2_), 1.95 (m. 2H, CH_2_), 1.68 (m. 2H, CH_2_); δ_C_ (400 MHz, DMSO-d_6_): 156.1, 145.5, 130.2 (q, J = 276.2 Hz), 127.0, 123.6, 122.0 (q, *J* = 28.4 Hz), 121.7, 118.1, 54.70, 49.8, 35.8, 29.3, 21.8. ESI-HRMS (m/z) [M-H]^-^: calculated for C15H18F3N6O3S 419.1119; found 419.1125.

#### Ethyl 4–(3-((1–(4-sulfamoylbutyl)-1H-1,2,3-triazol-4-yl)methyl)ureido)benzoate (47)

Compound **47** was obtained according to the general procedure earlier reported with **6** and **24** as starting materials. Yield 37%; m.p. 259–261 °C; silica gel TLC R*_f_* 0.16 (MeOH/DCM 10% *v/v*); δ_H_ (400 MHz, DMSO-d_6_): 9.07 (s, 1H, exchange with D_2_O, NHCONH), 8.00 (s, 1H, Ar-H), 7.86 (d, J = 8.0 Hz, 2H, Ar-H), 7.55 (d, *J* = 8.0 Hz, 2H, Ar-H), 6.81 (s, 2H, exchange with D_2_O, SO_2_NH_2_), 6.66 (t, *J* = 5.7 Hz, 1H, exchange with D_2_O, NHCONH), 4.37 (m, 4H, 2 x CH_2_), 4.28 (q, *J* = 6.8 Hz, 2H, CH_2_), 302 (t, *J* = 6.4 Hz, 2H, CH_2_), 1.94 (m. 2H, CH_2_), 1.68 (m. 2H, CH_2_), 1.32 (t, *J* = 6.8 Hz, 3H, CH_3_); δ_C_ (400 MHz, DMSO-d_6_): 166.6, 158.5, 155.6, 146.0, 131.5, 123.8, 123.1, 117.9, 61.3, 54.7, 50.1, 36.4, 29.6, 22.2, 15.4. ESI-HRMS (m/z) [M-H]^-^: calculated for C17H23N6O5S 423.1456; found 423.1461.

#### 4–(4-((3–(3-Methoxyphenyl)ureido)methyl)-1H-1,2,3-triazol-1-yl)butane-1-sulfonamide (48)

Compound **48** was obtained according to the general procedure earlier reported with **6** and **25** as starting materials. Yield 28%; m.p. 217–218 °C; silica gel TLC R*_f_* 0.24 (MeOH/DCM 10% *v/v*); δ_H_ (400 MHz, DMSO-d_6_): 8.69 (s, 1H, exchange with D_2_O, NHCONH), 7.99 (s, 1H, Ar-H), 7.17 (t, J = 2.0 Hz, 1H, Ar-H), 7.13 (t, *J* = 8.4 Hz, 1H, Ar-H), 6.88 (dd, *J* = 8.4 2.0 Hz, 1H, Ar-H), 6.82 (s, 2H, exchange with D_2_O, SO_2_NH_2_), 6.68 (t, *J* = 5.7 Hz, 1H, exchange with D_2_O, NHCONH), 6.49 (dd, *J* = 8.4 2.0 Hz, 1H, Ar-H), 4.38 (t, *J* = 6.5 Hz, 2H, CH_2_), 4.34 (d, *J* = 5.7 Hz, 2H, CH_2_), 3.72 (s, 3H, CH_3_), 3.02 (t, *J* = 6.5 Hz, 2H, CH_2_), 1.93 (m. 2H, CH_2_), 1.67 (m. 2H, CH_2_); δ_C_ (400 MHz, DMSO-d_6_): 160.7, 156.0, 146.5, 142.6, 130.4, 123.5, 111.1, 107.6, 104.5, 55.9, 45.8, 49.8, 35.9, 29.4, 21.8. ESI-HRMS (m/z) [M-H]^-^: calculated for C15H21N6O4S 381.1350; found 381.1356.

#### 4–(4-((3–(4-Methoxy-2-methylphenyl)ureido)methyl)-1H-1,2,3-triazol-1-yl)butane-1-sulfonamide (49)

Compound **49** was obtained according to the general procedure earlier reported with **6** and **26** as starting materials. Yield 24%; m.p. 282–285 °C; silica gel TLC R*_f_* 0.08 (MeOH/DCM 8% *v/v*); δ_H_ (400 MHz, DMSO-d_6_): 7.99 (s, 1H, exchange with D_2_O, NHCONH), 7.64 (s, 1H, Ar-H), 7.54 (d, *J* = 8.0 Hz, 1H, Ar-H), 6.82 (s, 2H, exchange with D_2_O, SO_2_NH_2_), 6.77 (t, *J* = 5.9 Hz, 1H, exchange with D_2_O, NHCONH), 6.75 (d, J = 2.4 Hz, 1H, Ar-H), 6.70 (dd, *J* = 8.0 2.4 Hz, 1H, Ar-H), 4.39 (t, *J* = 6.6 Hz, 2H, CH_2_), 4.32 (d, *J* = 5.9 Hz, 2H, CH_2_), 3.71 (s, 3H, CH_3_), 3.02 (t, *J* = 6.6 Hz, 2H, CH_2_), 2.16 (s, 3H, CH_3_), 1.94 (m. 2H, CH_2_), 1.68 (m. 2H, CH_2_); δ_C_ (400 MHz, DMSO-d_6_): 157.3, 155.9, 146.7, 132.0, 131.3, 124.6, 123.6, 116.4, 112.1, 56.1, 54.7, 49.8, 36.0, 29.4, 21.8, 19.1. ESI-HRMS (m/z) [M-H]^-^: calculated for C16H23N6O4S 395.1507; found 395.1512.

#### Ethyl 1–(4-sulfamoylbutyl)-1H-1,2,3-triazole-4-carboxylate (50)

Compound **50** was obtained according to the general procedure earlier reported with **6** and *ethyl propiolate* as starting materials. Yield 95%; m.p. 180–181 °C; silica gel TLC R*_f_* 0.09 (MeOH/DCM 10% *v/v*); δ_H_ (400 MHz, DMSO-d_6_): 8.82 (s, 1H, Ar-H), 6.81 (s, 2H, exchange with D_2_O, SO_2_NH_2_), 4.48 (t, *J* = 6.2 Hz, 2H, CH_2_), 4.33 (q, *J* = 6.4 Hz, 2H, CH_2_), 3.03 (t, *J* = 6.2 Hz, 2H, CH_2_), 2.00 (m, 2H, CH_2_), 1.65 (m, 2H, CH_2_), 1.33 (t, *J* = 6.4 Hz, 3H, CH_3_); δ_C_ (400 MHz, DMSO-d_6_): 161.3, 139.7, 130.0, 61.5, 54.7, 50.3, 29.0, 21.6, 12.2. ESI-HRMS (m/z) [M-H]^-^: calculated for C9H15N4O4S 275.0819; found 275.0825.

#### 4–(4-Phenyl-1H-1,2,3-triazol-1-yl)butane-1-sulfonamide (51)

Compound **51** was obtained according to the general procedure earlier reported with **6** and *ethynylbenzene* as starting materials. Yield 45%; m.p. 199–201 °C; silica gel TLC R*_f_* 0.14 (MeOH/DCM 10% *v/v*); δ_H_ (400 MHz, DMSO-d_6_): 8.62 (s, 1H, Ar-H), 7.86 (d, *J* = 8.3 Hz, 2H, Ar-H), 7.47 (t, *J* = 8.3 Hz, 2H, Ar-H), 7.35 (t, *J* = 8.3 Hz, 1H, Ar-H), 6.81 (s, 2H, exchange with D_2_O, SO_2_NH_2_), 4.46 (t, *J* = 6.3 Hz, 2H, CH_2_), 3.04 (t, *J* = 6.3 Hz, 2H, CH_2_), 2.02 (m, 2H, CH_2_), 1.61 (m, 2H, CH_2_); δ_C_ (400 MHz, DMSO-d_6_): 147.3, 131.9, 129.9, 128.8, 126.1, 122.3, 54.7, 50.1, 29.2, 21.8. ESI-HRMS (m/z) [M-H]^-^: calculated for C12H15N4O2S 279.0921; found 279.0926.

#### 4–(4-(3-Fluorophenyl)-1H-1,2,3-triazol-1-yl)butane-1-sulfonamide (52)

Compound **52** was obtained according to the general procedure earlier reported with **6** and *1-ethynyl-3-fluorobenzene* as starting materials. Yield 37%; m.p. 226–229 °C; silica gel TLC R*_f_* 0.14 (MeOH/DCM 10% *v/v*); δ_H_ (400 MHz, DMSO-d_6_): 8.69 (s, 1H, Ar-H), 7.72 (d, *J* = 8.0 Hz, 1H, Ar-H), 7.67 (dt, *J* = 10.6 2.2 Hz, 1H, Ar-H), 7.52 (q, *J* = 8.0 Hz, 1H, Ar-H), 7.19 (td, *J* = 8.0 2.2 Hz, 1H, Ar-H), 6.82 (s, 2H, exchange with D_2_O, SO_2_NH_2_), 4.47 (t, *J* = 6.3 Hz, 2H, CH_2_), 3.05 (t, *J* = 6.3 Hz, 2H, CH_2_), 2.02 (m, 2H, CH_2_), 1.70 (m, 2H, CH_2_); δ_C_ (400 MHz, DMSO-d_6_): 164.7 (d, *J* = 219.4 Hz), 146.3, 134.3 (d, *J* = 9.3 Hz), 132.2 (d, *J* = 9.3 Hz), 123.1, 122.2, 115.7 (d, *J* = 23.8 Hz), 112.8 (d, *J* = 23.8 Hz), 54.6, 50.2, 29.1, 21.8. ESI-HRMS (m/z) [M-H]^-^: calculated for C12H14FN4O2S 297.0827; found 297.0833.

### Carbonic anhydrase inhibition

An Applied Photophysics stopped-flow instrument has been used for assaying the CA catalysed CO_2_ hydration activity^47^. Phenol red (at a concentration of 0.2 mM) has been used as indicator, working at the absorbance maximum of 557 nm, with 20 mM Hepes (pH 7.5) as buffer and 20 mM Na_2_SO_4_ (for maintaining constant the ionic strength), following the initial rates of the CA-catalysed CO_2_ hydration reaction for a period of 10 − 100 s. The CO_2_ concentrations ranged from 1.7 to 17 mM for the determination of the kinetic parameters and inhibition constants. For each inhibitor, at least six traces of the initial 5 − 10% of the reaction have been used for determining the initial velocity. The uncatalysed rates were determined in the same manner and subtracted from the total observed rates. Stock solutions of inhibitor (0.1 mM) were prepared in distilled − deionized water and dilutions up to 0.01 nM were done thereafter with the assay buffer. Inhibitor and enzyme solutions were preincubated together for 15 min at room temperature before assay to allow for the formation of the E − I complex. The inhibition constants were obtained by nonlinear least-squares methods using PRISM 3 and the Cheng − Prusoff equation, as reported earlier^48^^,^^49^, and represent the mean from at least three different determinations. The enzyme concentrations were in the range 6–14 nM. All hCA isoforms were recombinant ones obtained in-house as reported earlier^50^.

### In silico studies

The crystal structure of hCA II (PDB 3K34)^51^^,^^52^ and hCA III (PDB 1Z93)^20^^,^^51^ used for computational studies were prepared using the Protein Preparation Wizard tool implemented in the Schrödinger suite, assigning bond orders, adding hydrogens, deleting water molecules, and optimising H-bonding networks. The energy minimisation protocol with a Root Mean Square Deviation (RMSD) value of 0.30 Å was applied using an Optimised Potentials for Liquid Simulation (OPLS4) force field^53–58^.

The 3D ligand structures were prepared by Maestro (v.13.4)^53^a and evaluated for their ionisation states at pH 7.4 ± 0.5 with Epik (v.5.7)^53^b. The conjugate gradient method in Macromodel (v.13.3)^53^c was used for energy minimisation (maximum iteration number: 2500; convergence criterion: 0.05 Kcal/mol/Å^2^).

For molecular docking studies the software Glide SP (v.9.2; default settings)^53^^d,^^55^ was used. In this regard, grids were centred in the centroid of the complexed ligand. The standard precision (SP) mode of the Glide Score function was applied to evaluate the predicted binding poses.

Molecular dynamics (MD) simulations were performed using Desmond Molecular Dynamics System (v.6.7)^53^e (Schrödinger suite) and OPLS4 force field. All systems were solvated in an orthorhombic box using simple point charge water molecules extended 15 Å away from any protein atom. The system was neutralised with 0.15 M Cl^-^ and Na^+^ ions. The simulation protocol included a starting relaxation step followed by a final production phase of 100 ns. In particular, the relaxation step comprised the following: (a) a stage of 100 ps at 10 K retaining the harmonic restraints on the solute heavy atoms (force constant of 50 Kcal/mol/Å^2^) using the NPT ensemble with Brownian dynamics; (b) a stage of 12 ps at 10 K with harmonic restraints on the solute heavy atoms (force constant of 50 Kcal/mol/Å^2^), using the NVT ensemble and Berendsen thermostat; (c) a stage of 12 ps at 10 K and 1 atm, retaining the harmonic restraints and using the NPT ensemble and Berendsen thermostat and barostat; (f) a stage of 12 ps at 300 K and 1 atm, retaining the harmonic restraints and using the NPT ensemble and Berendsen thermostat and barostat; (g) a final 24 ps stage at 300 K and 1 atm without harmonic restraints, using the NPT Berendsen thermostat and barostat. The final production phase of MD was run using a canonical NPT Berendsen ensemble at 300 K. During the MD simulation, a time step of 2 fs was used while constraining the bond lengths of H atoms with the M-SHAKE algorithm. The atomic coordinates of the system were saved every 100 ps along the MD trajectory. Protein RMSD, ligand RMSD/RMSF (Root Mean Square Fluctuation) ligand torsions evolution and occurrence of intermolecular H-bonds and hydrophobic contacts were provided by the Simulation Interaction Diagram (SID) implemented in Maestro along with the production phase of the MD simulation. The tool reads the MD trajectory file and identifies ligand/target interactions repeatedly occurring during the simulation time (for instance, a 60% value suggests that the interaction is maintained for 60% of the MD). The 1000 frames resulting from MDs were clustering using the Conformer Cluster tool implemented in the Schrödinger suite in 10 clusters. Figures were generated with Chimera^59^.

## Supplementary Material

Supplemental Material
