# Supplementary material for: Discovery of the first-in-class potent and isoform-selective human carbonic anhydrase III inhibitors
Source: J Enzyme Inhib Med Chem. 2023 Apr 24;38(1):2202360. doi: 10.1080/14756366.2023.2202360 (PMC10128460; doi:10.1080/14756366.2023.2202360)
Supplement: Supplemental Material [file IENZ_A_2202360_SM6400.pdf]

Supporting Information

Discovery of the first-in-class potent and isoform-selective human carbonic anhydrase III inhibitors

Simone Giovannuzzi,[a,1] Alessandro Bonardi,[b,1] Paola Gratteri,[b] Alessio Nocentini,[a,b*] Claudiu T. Supuran[a,*]

a. NEUROFARBA Department, Pharmaceutical and Nutraceutical Section, University of Florence, Via U. Schiff 6, 50019 Sesto Fiorentino, Firenze, Italy.

b. NEUROFARBA Department, Laboratory of Molecular Modeling Cheminformatics & QSAR, University of Florence, Via U. Schiff 6, 50019 Sesto Fiorentino, Firenze, Italy.

1. These authors contributed equally to this work

Additional drug design information **S2**

^{1}H-, ^{13}C-NMR spectra **S3**

HPLC chromatograms **S29**

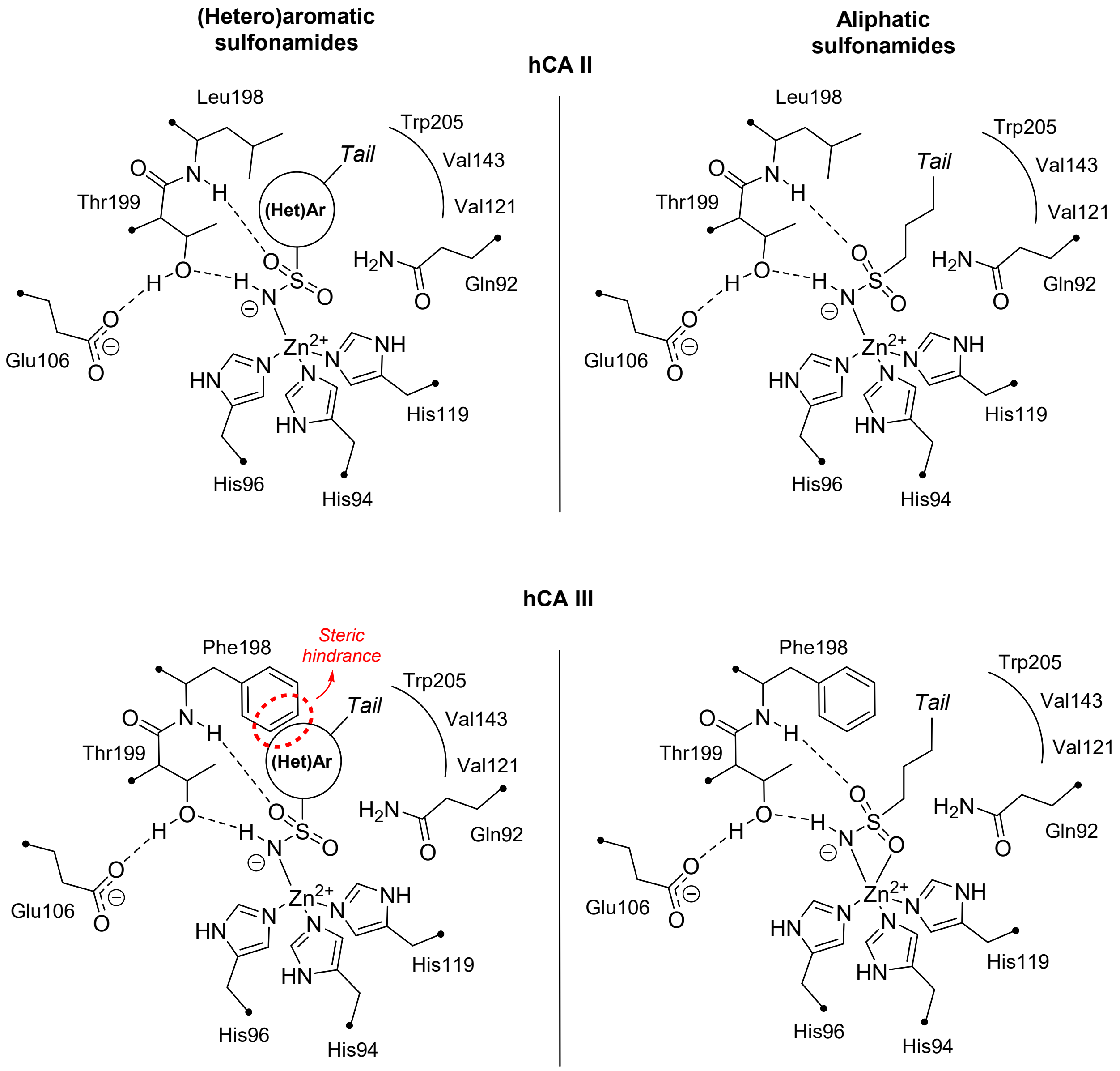

Figure S1. 2D drawing of the binding mode of (hetero)aromatic sulfonamides and aliphatic sulfonamides in the hCA II and hCA III active sites.

[1]H-27

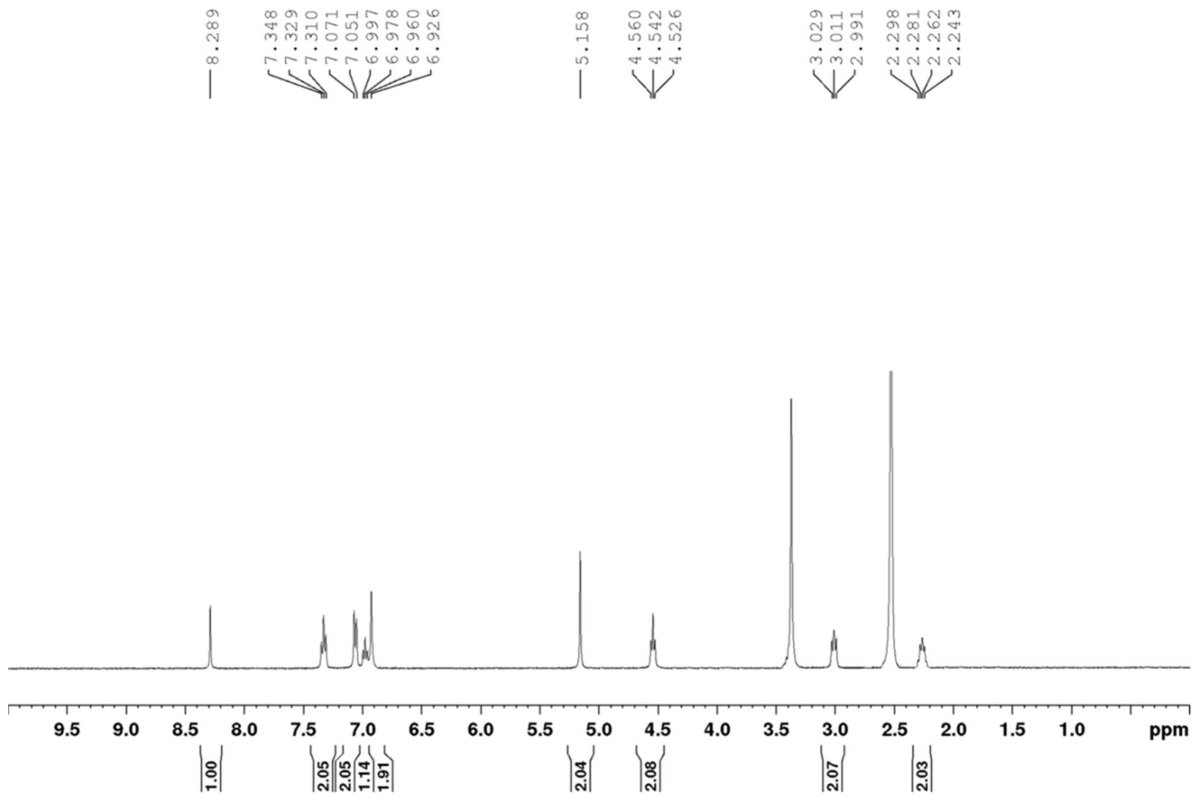
8.289
7.348
7.329
7.310
7.071
7.051
6.997
6.978
6.960
6.926
5.158
4.560
4.542
4.526
3.029
3.011
2.991
2.298
2.281
2.262
2.243
9.5 9.0 8.5 8.0 7.5 7.0 6.5 6.0 5.5 5.0 4.5 4.0 3.5 3.0 2.5 2.0 1.5 1.0 ppm
1.00
2.05
2.05
1.14
1.91
2.04
2.08
2.07
2.03

[13]C-27

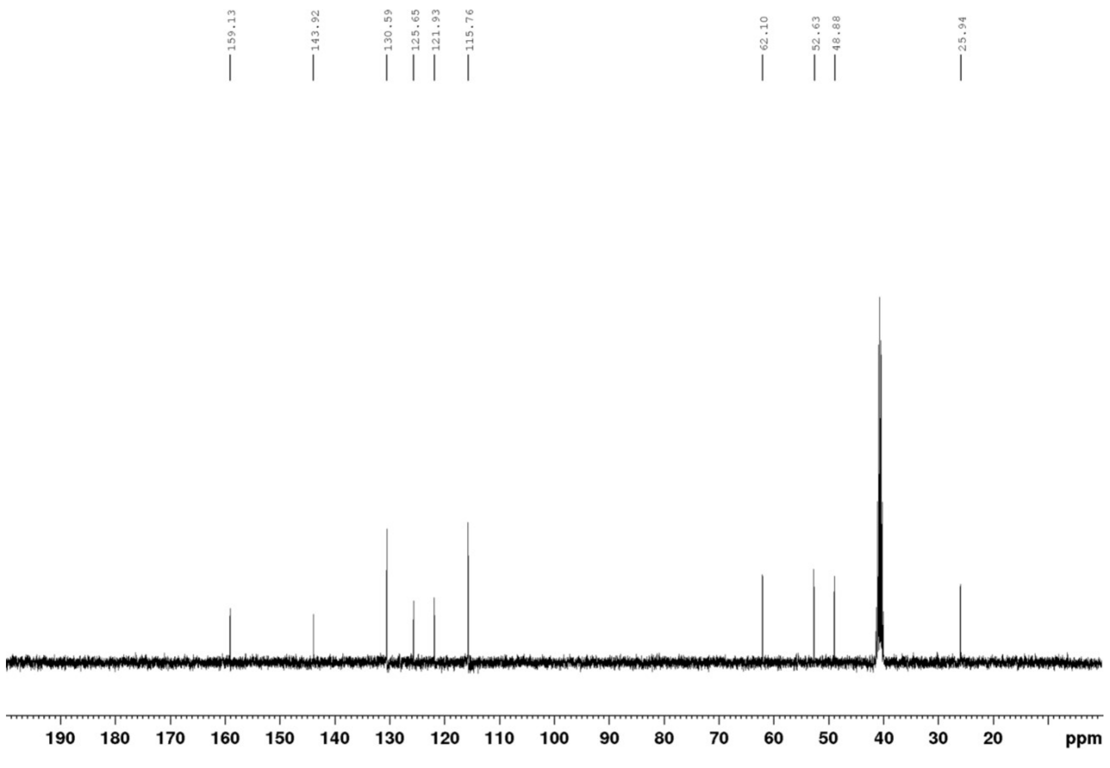
159.13
143.92
130.59
125.65
121.93
115.76
62.10
52.63
48.88
25.94
190 180 170 160 150 140 130 120 110 100 90 80 70 60 50 40 30 20 ppm

^{1}H-28

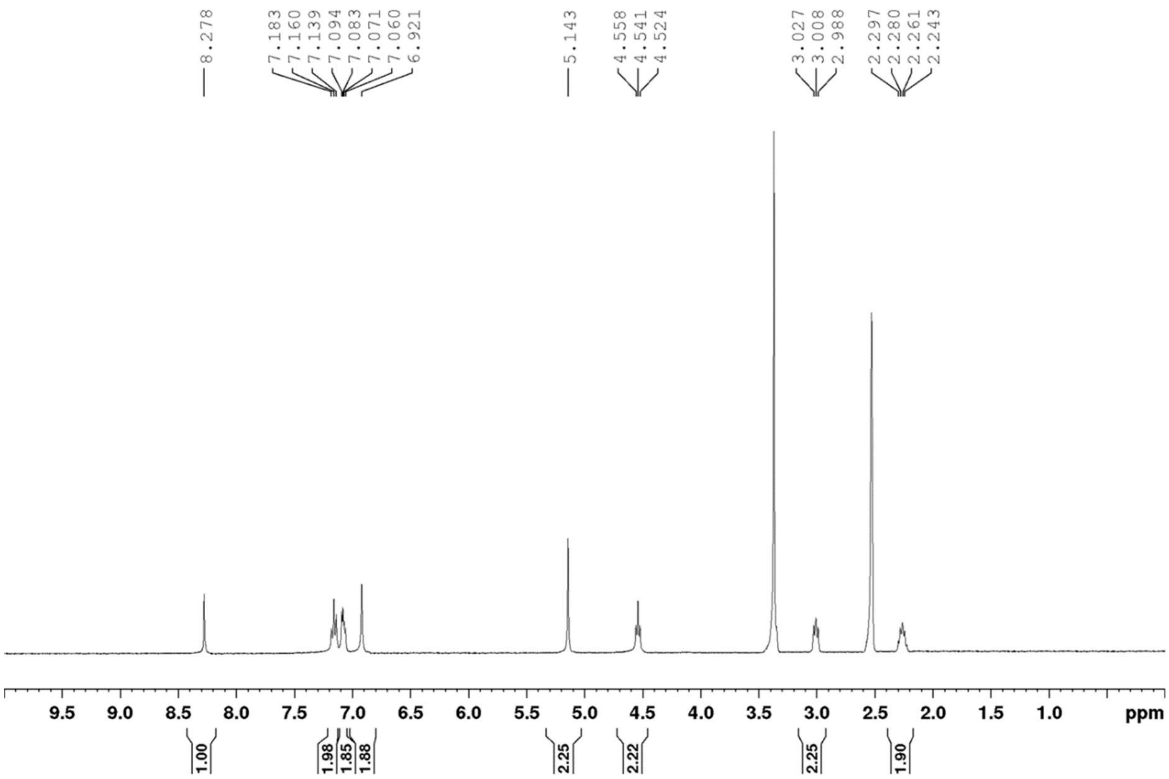
8.278
7.183
7.160
7.139
7.094
7.083
7.071
7.060
6.921
5.143
4.558
4.541
4.524
3.027
3.008
2.988
2.297
2.280
2.261
2.243
9.5 9.0 8.5 8.0 7.5 7.0 6.5 6.0 5.5 5.0 4.5 4.0 3.5 3.0 2.5 2.0 1.5 1.0 ppm
1.00
1.98
1.85
1.88
2.25
2.22
2.25
1.90

^{13}C-28

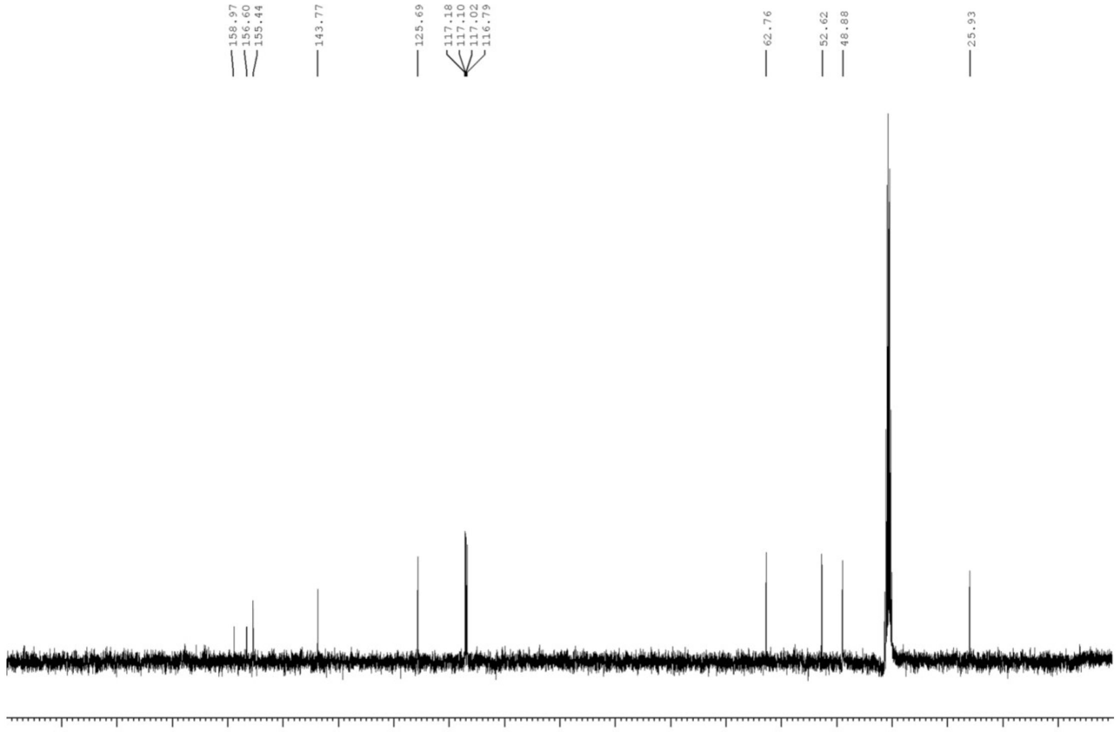
158.97
156.60
155.44
143.77
125.69
117.18
117.10
117.02
116.79
62.76
52.62
48.88
25.93
190 180 170 160 150 140 130 120 110 100 90 80 70 60 50 40 30 20 ppm

¹H-29

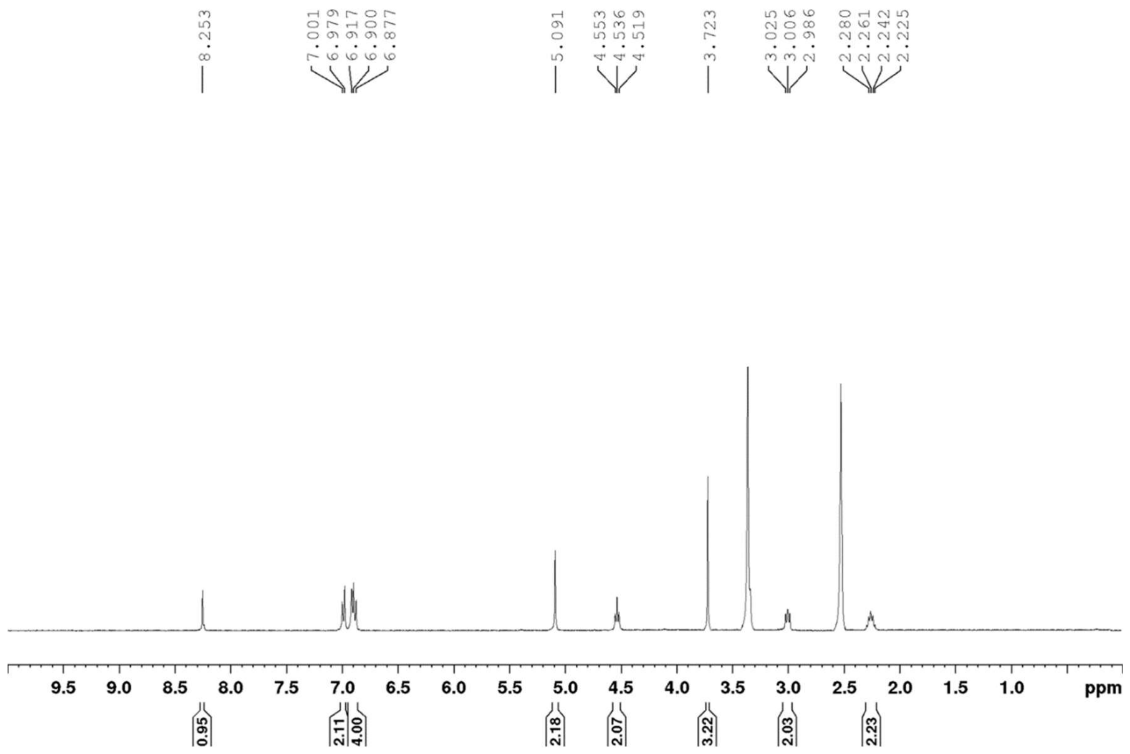
8.253
7.001
6.979
6.917
6.900
6.877
5.091
4.553
4.536
4.519
3.723
3.025
3.006
2.986
2.280
2.261
2.242
2.225
9.5 9.0 8.5 8.0 7.5 7.0 6.5 6.0 5.5 5.0 4.5 4.0 3.5 3.0 2.5 2.0 1.5 1.0 ppm
0.95
2.11
4.00
2.18
2.07
3.22
2.03
2.23

¹³C-29

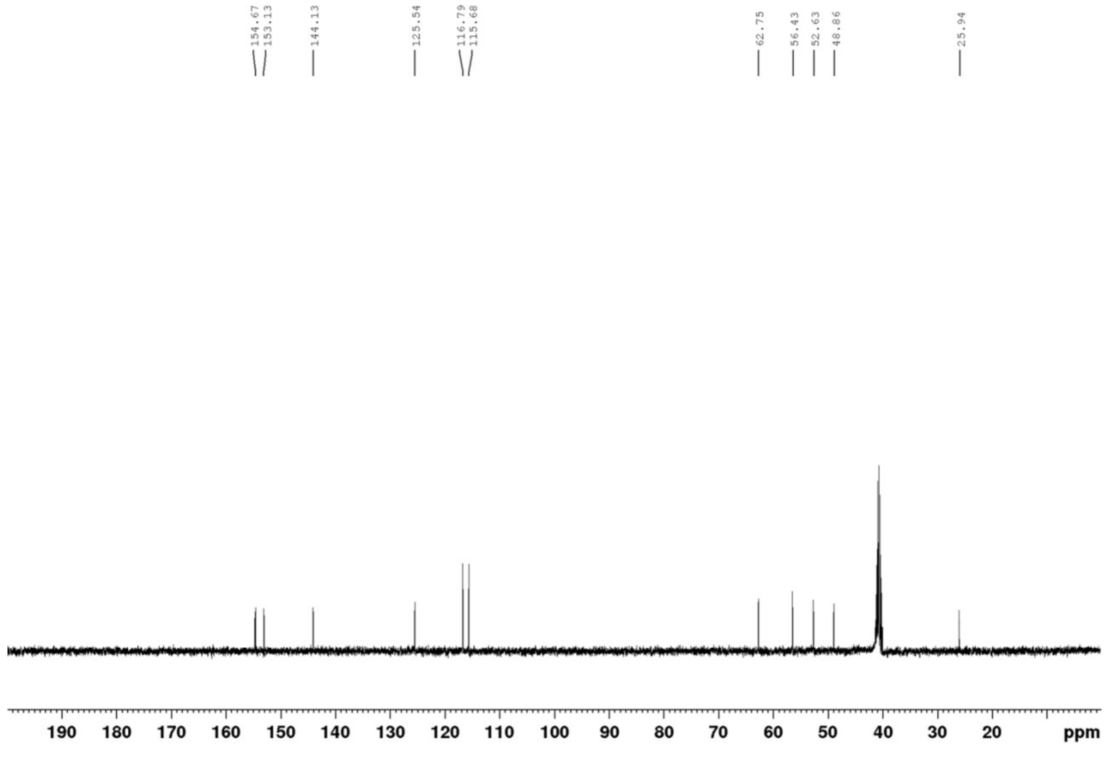
154.67
153.13
144.13
125.54
116.79
115.68
62.75
56.43
52.63
48.86
25.94
190 180 170 160 150 140 130 120 110 100 90 80 70 60 50 40 30 20 ppm

^{1}H-30

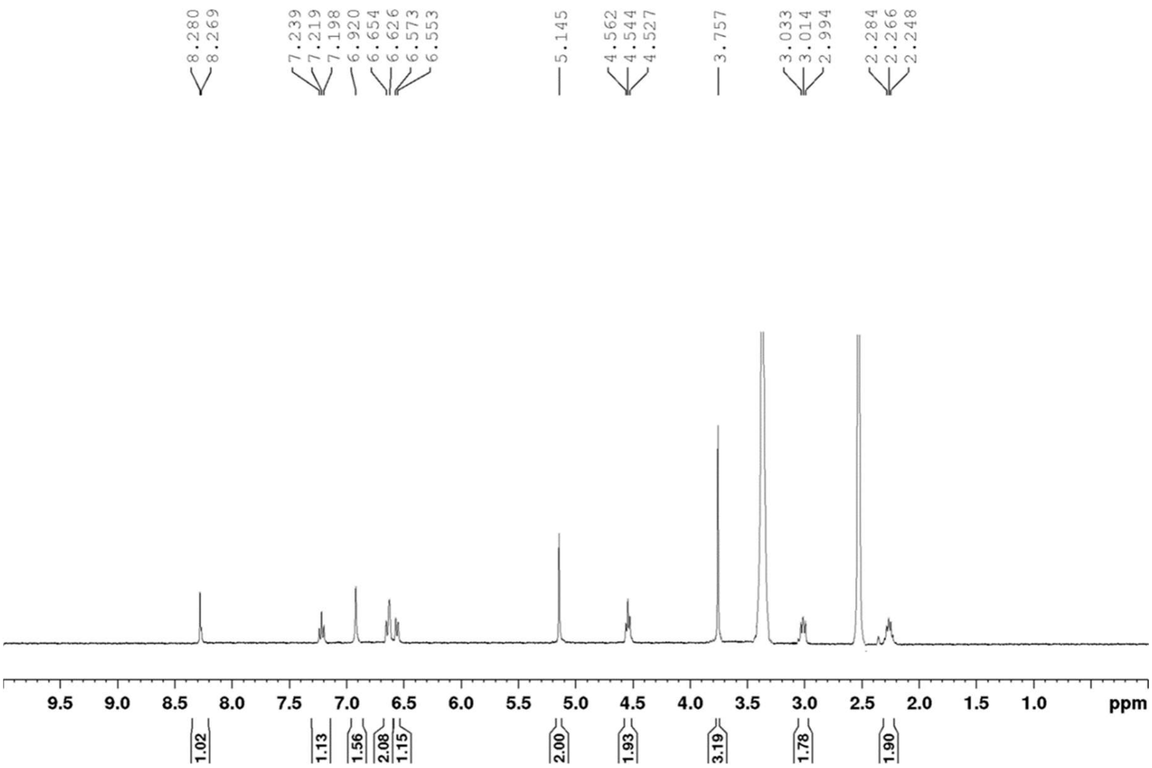
8.280 8.269
7.239 7.219 7.198 6.920 6.654 6.626 6.573 6.553
5.145
4.562 4.544 4.527
3.757
3.033 3.014 2.994
2.284 2.266 2.248
9.5 9.0 8.5 8.0 7.5 7.0 6.5 6.0 5.5 5.0 4.5 4.0 3.5 3.0 2.5 2.0 1.5 1.0 ppm
1.02 1.13 1.56 2.08 1.15 2.00 1.93 3.19 1.78 1.90

^{13}C-30

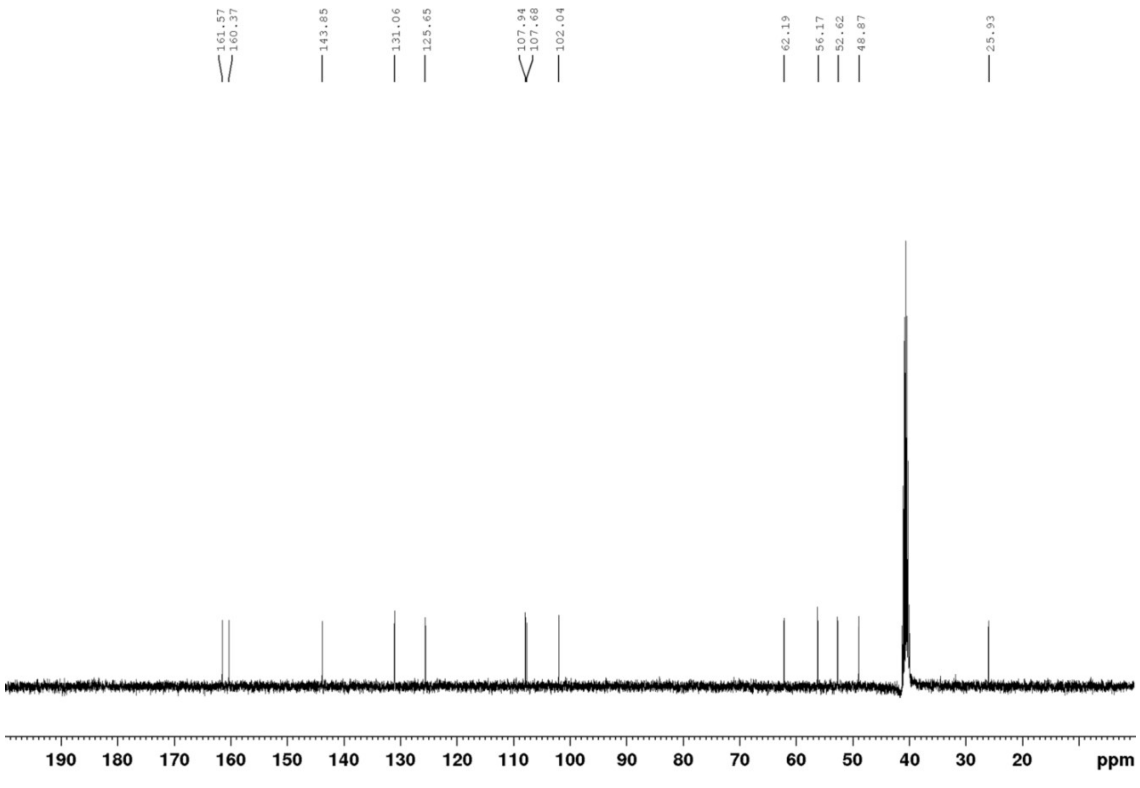
161.57 160.37
143.85
131.06
125.65
107.94 107.68
102.04
62.19
56.17
52.62
48.87
25.93
190 180 170 160 150 140 130 120 110 100 90 80 70 60 50 40 30 20 ppm

¹H-31

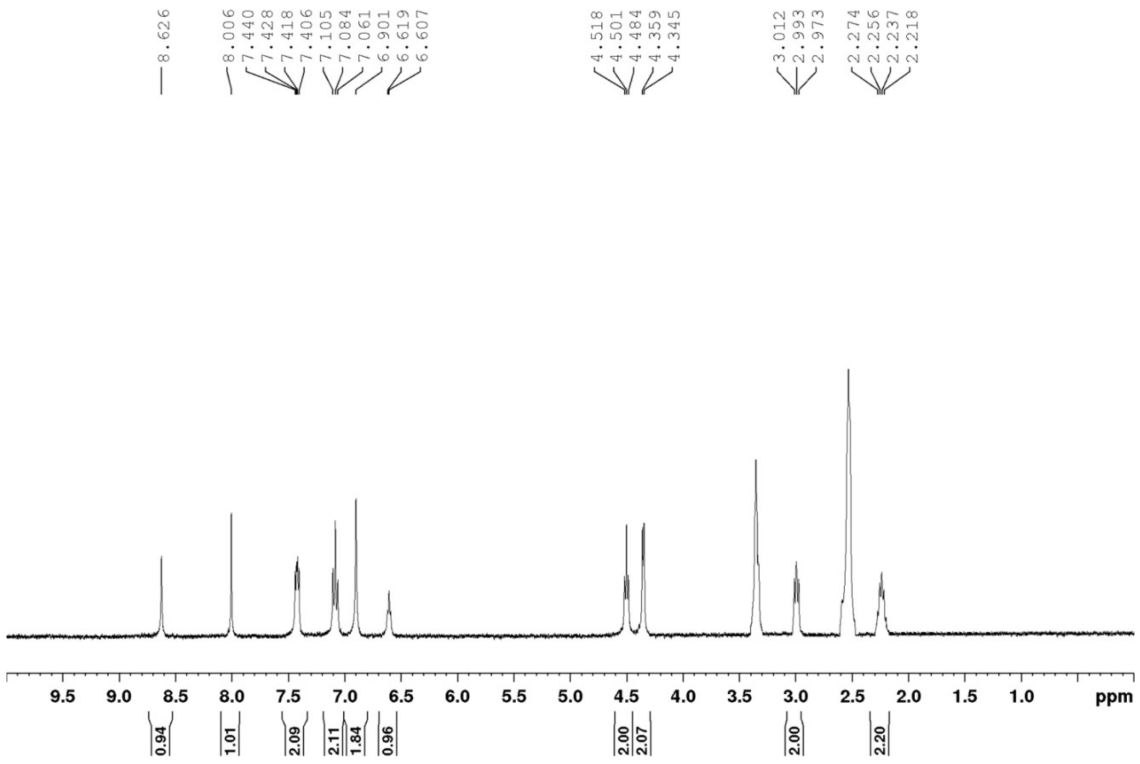

¹³C-31

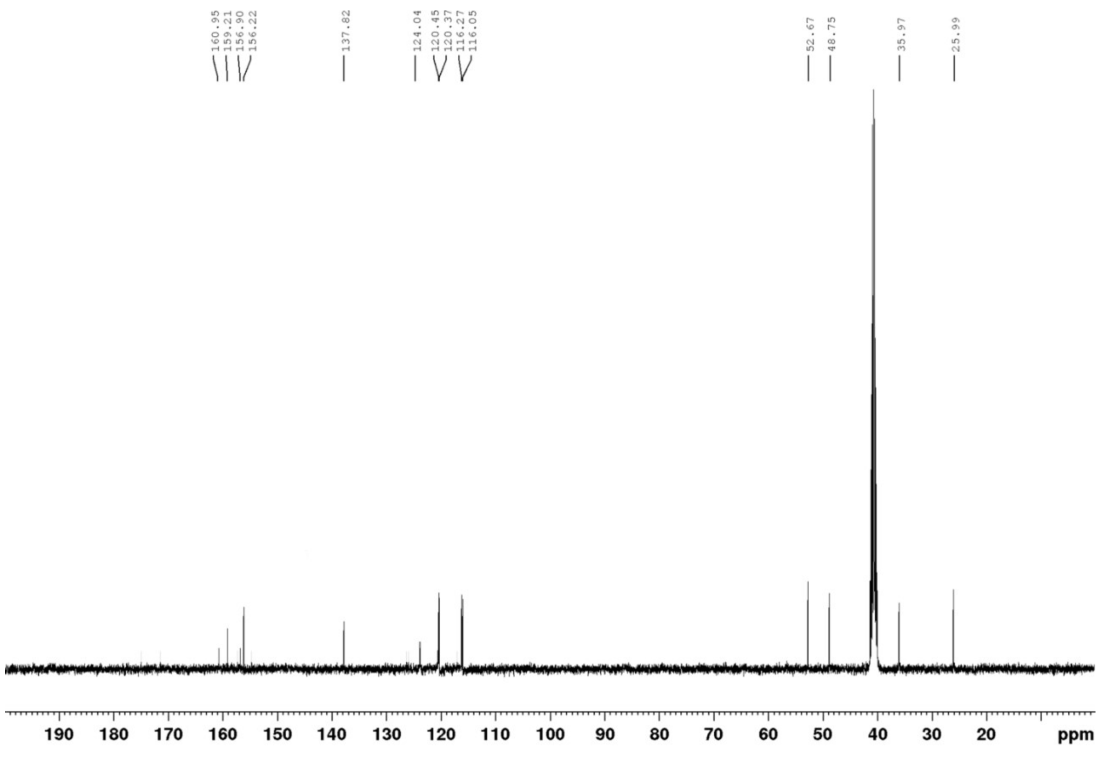

^{1}H-32

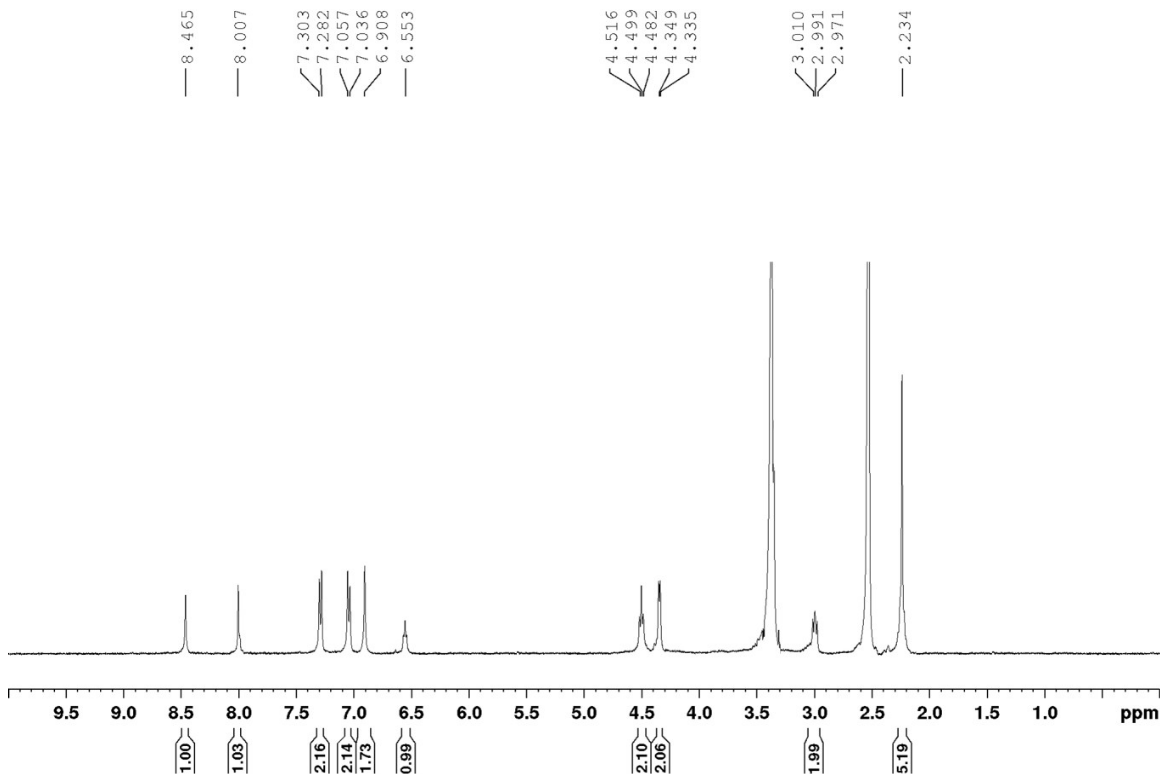
8.465
8.007
7.303
7.282
7.057
7.036
6.908
6.553
4.516
4.499
4.482
4.349
4.335
3.010
2.991
2.971
2.234
9.5 9.0 8.5 8.0 7.5 7.0 6.5 6.0 5.5 5.0 4.5 4.0 3.5 3.0 2.5 2.0 1.5 1.0 ppm
1.00
1.03
2.16
2.14
1.73
0.99
2.10
2.06
1.99
5.19

^{13}C-32

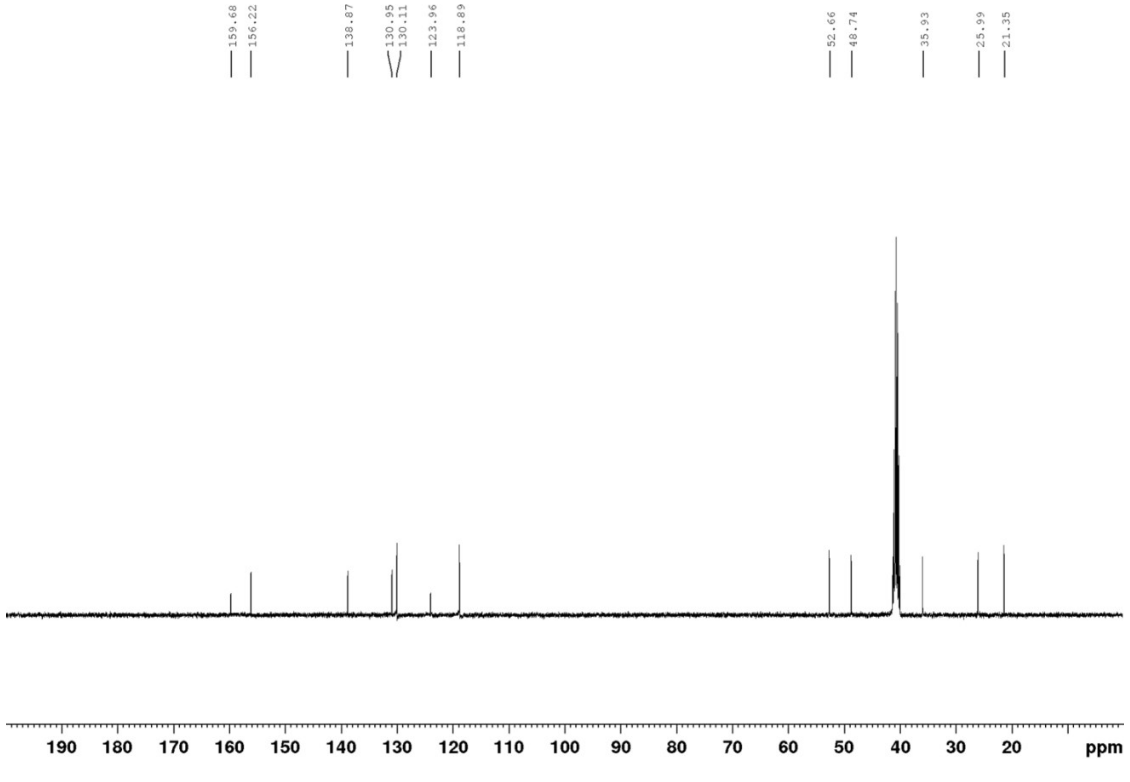
159.68
156.22
138.87
130.95
130.11
123.96
118.89
52.66
48.74
35.93
25.99
21.35
190 180 170 160 150 140 130 120 110 100 90 80 70 60 50 40 30 20 ppm

^{1}H-33

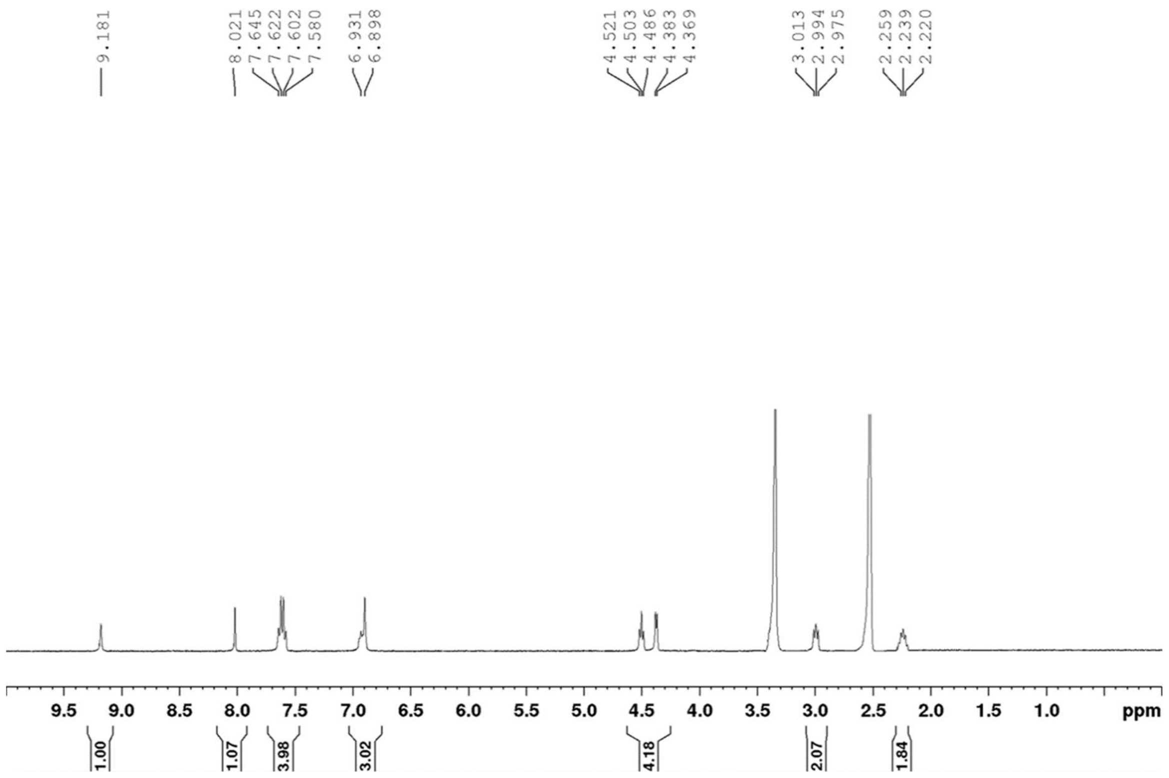

9.181
8.021
7.645
7.622
7.602
7.580
6.931
6.898
4.521
4.503
4.486
4.383
4.369
3.013
2.994
2.975
2.259
2.239
2.220
9.5 9.0 8.5 8.0 7.5 7.0 6.5 6.0 5.5 5.0 4.5 4.0 3.5 3.0 2.5 2.0 1.5 1.0 ppm
1.00
1.07
3.98
3.02
4.18
2.07
1.84

^{13}C-33

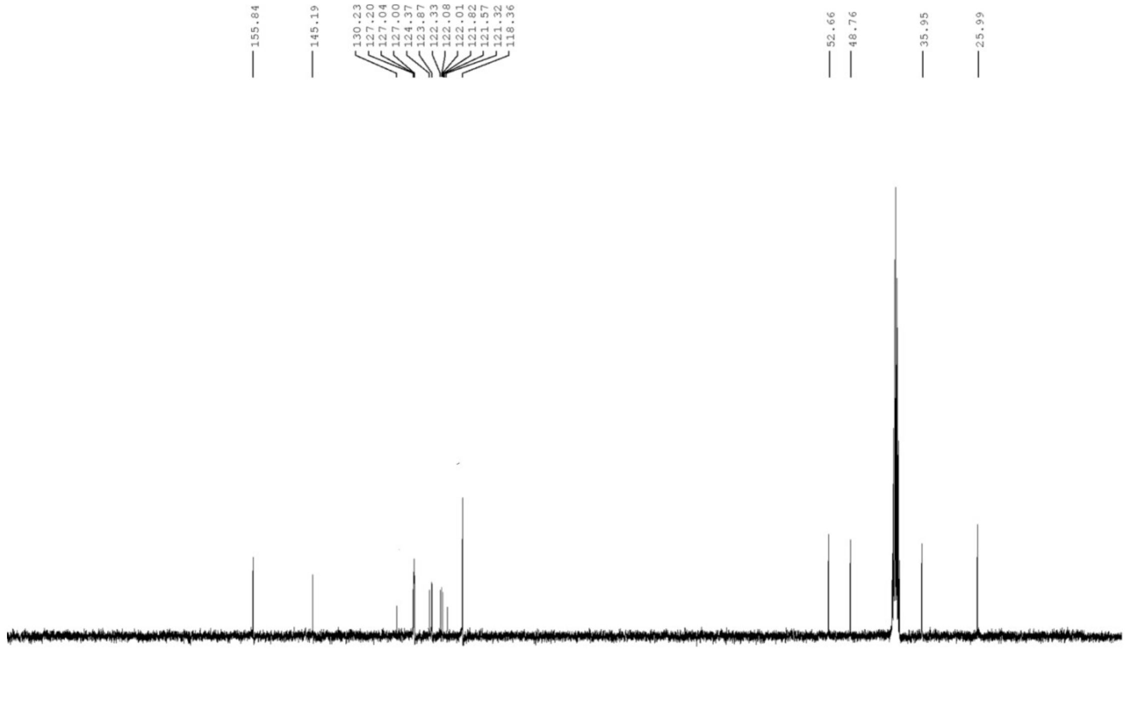

155.84
145.19
130.23
127.20
127.04
127.00
124.37
123.87
122.33
122.08
122.01
121.82
121.57
121.32
118.36
52.66
48.76
35.95
25.99
190 180 170 160 150 140 130 120 110 100 90 80 70 60 50 40 30 20 ppm

^{1}H-34

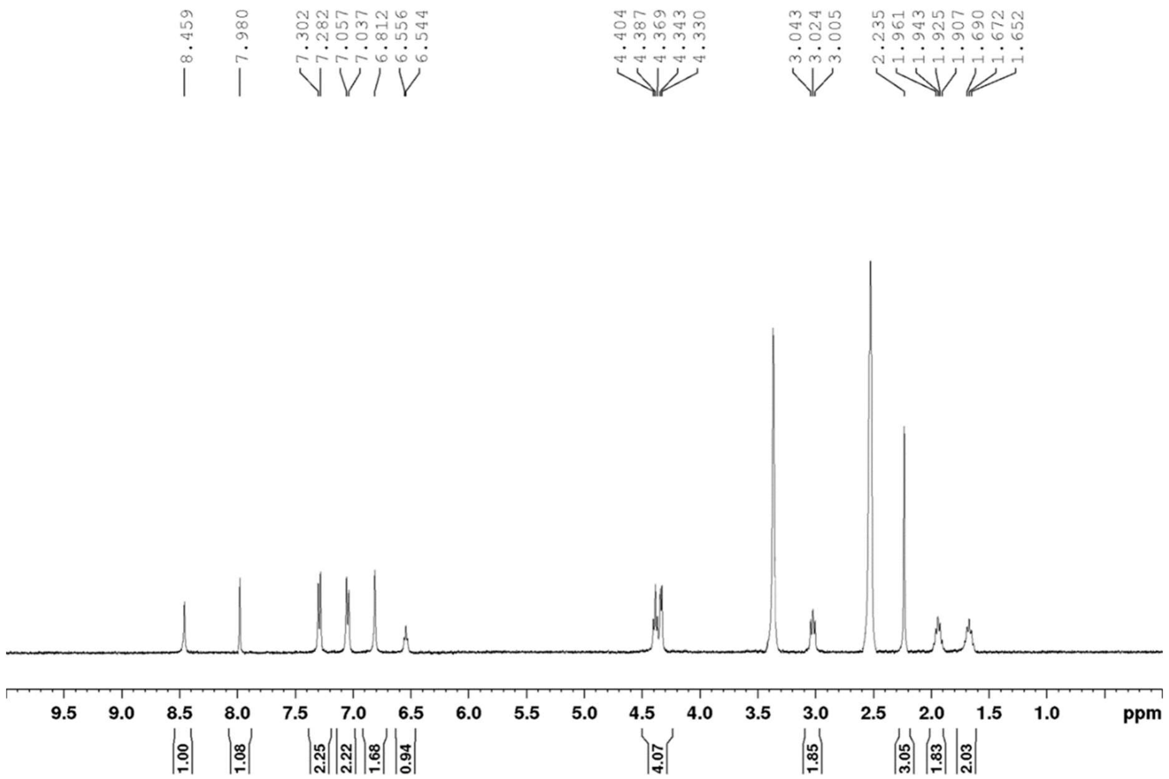
8.459
7.980
7.302
7.282
7.057
7.037
6.812
6.556
6.544
4.404
4.387
4.369
4.343
4.330
3.043
3.024
3.005
2.235
1.961
1.943
1.925
1.907
1.690
1.672
1.652
9.5 9.0 8.5 8.0 7.5 7.0 6.5 6.0 5.5 5.0 4.5 4.0 3.5 3.0 2.5 2.0 1.5 1.0 ppm
1.00
1.08
2.25
2.22
1.68
0.94
4.07
1.85
3.05
1.83
2.03

^{13}C-34

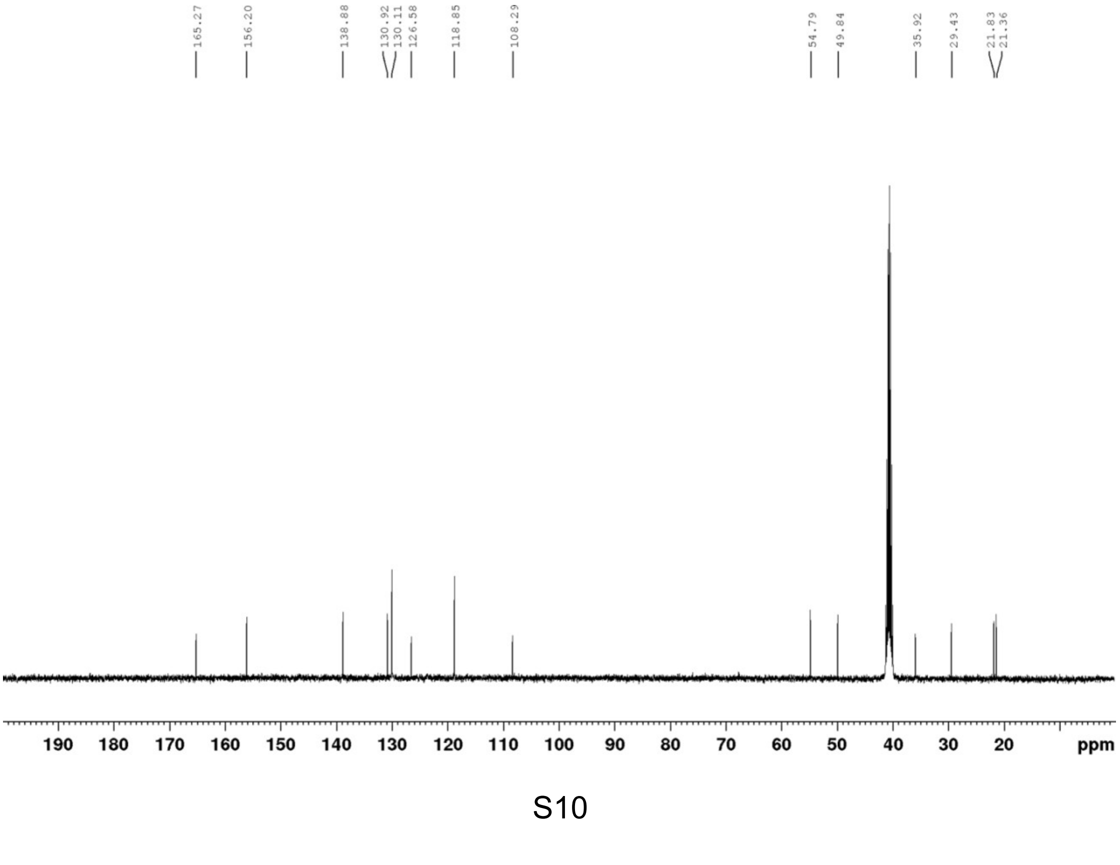
165.27
156.20
138.88
130.92
130.11
126.58
118.85
108.29
54.79
49.84
35.92
29.43
21.83
21.36
190 180 170 160 150 140 130 120 110 100 90 80 70 60 50 40 30 20 ppm

^{1}H-35

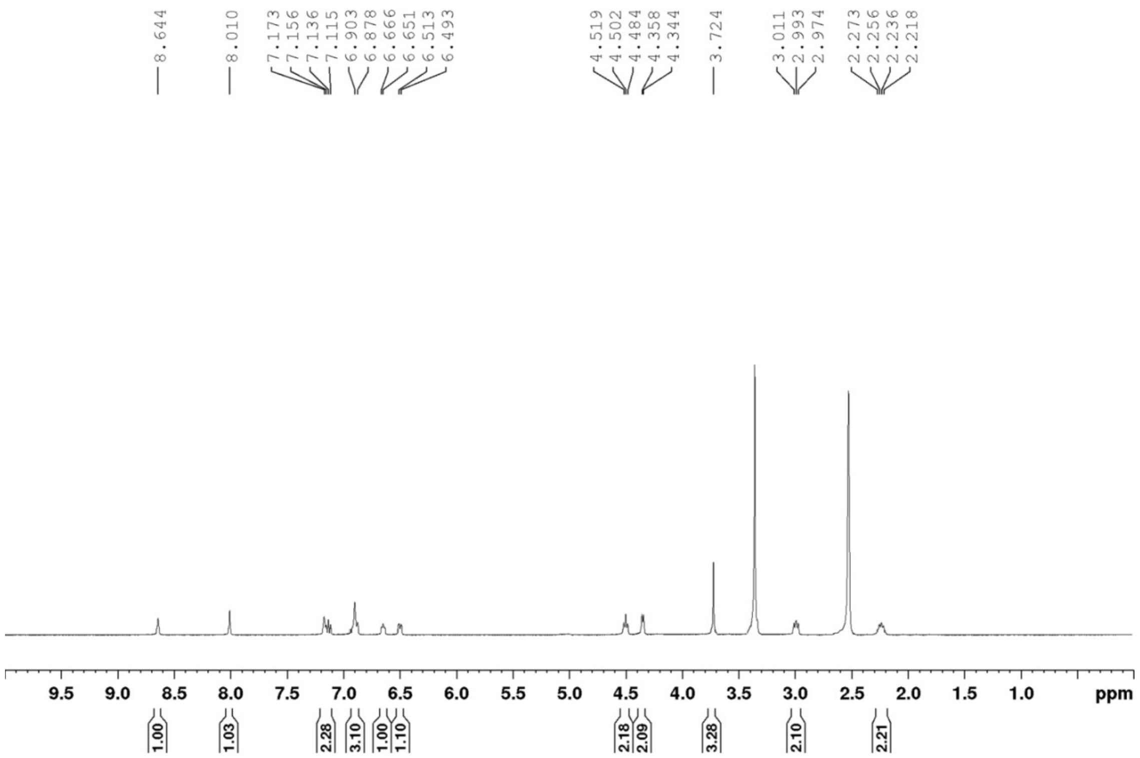
8.644
8.010
7.173
7.156
7.136
7.115
6.903
6.878
6.666
6.651
6.513
6.493
4.519
4.502
4.484
4.358
4.344
3.724
3.011
2.993
2.974
2.273
2.256
2.236
2.218
9.5 9.0 8.5 8.0 7.5 7.0 6.5 6.0 5.5 5.0 4.5 4.0 3.5 3.0 2.5 2.0 1.5 1.0 ppm
1.00
1.03
2.28
3.10
1.00
1.10
2.18
2.09
3.28
2.10
2.21

^{13}C-35

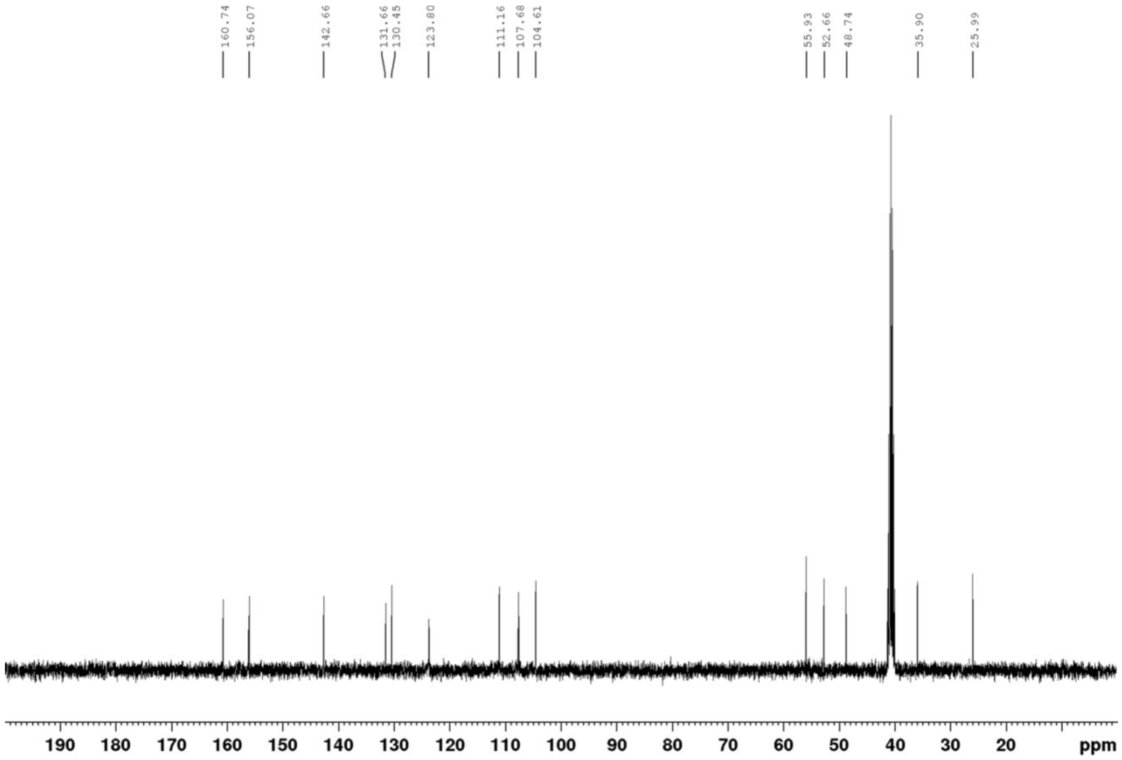
160.74
156.07
142.66
131.66
130.45
123.80
111.16
107.68
104.61
55.93
52.66
48.74
35.90
25.99
190 180 170 160 150 140 130 120 110 100 90 80 70 60 50 40 30 20 ppm

[1]H-36

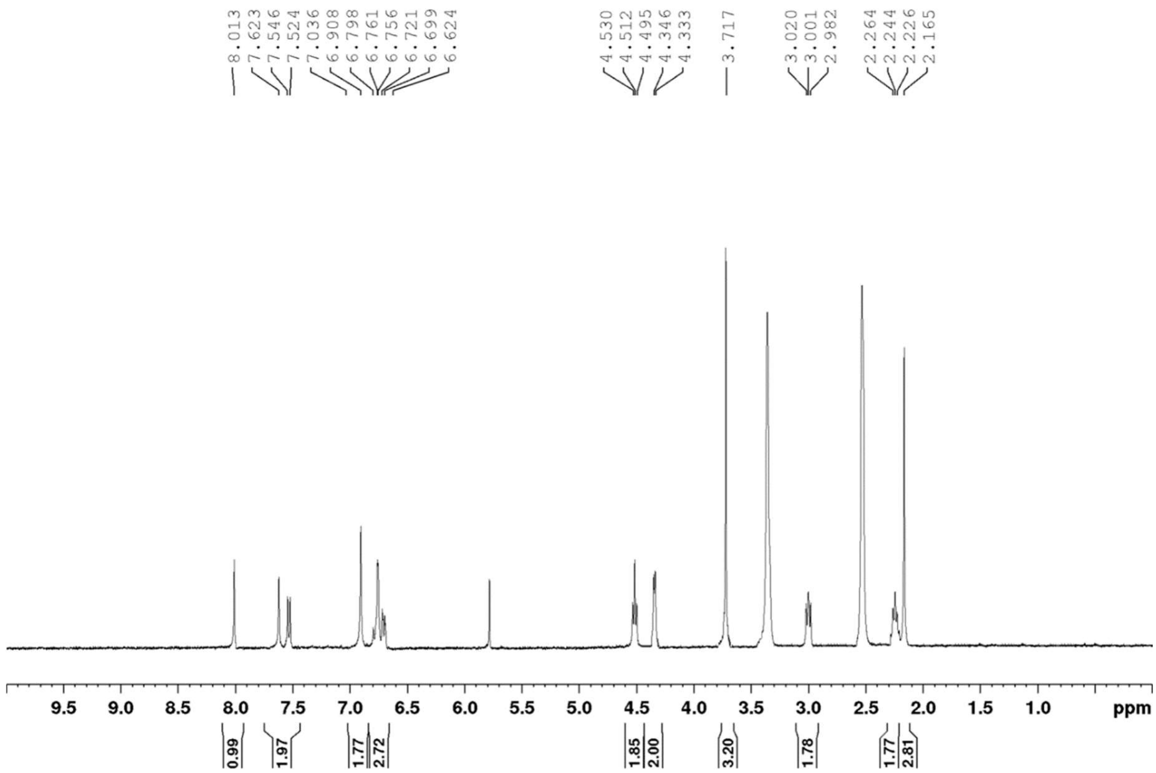
8.013
7.623
7.546
7.524
7.036
6.908
6.798
6.761
6.756
6.721
6.699
6.624
4.530
4.512
4.495
4.346
4.333
3.717
3.020
3.001
2.982
2.264
2.244
2.226
2.165
9.5 9.0 8.5 8.0 7.5 7.0 6.5 6.0 5.5 5.0 4.5 4.0 3.5 3.0 2.5 2.0 1.5 1.0 ppm
0.99
1.97
1.77
2.72
1.85
2.00
3.20
1.78
1.77
2.81

[13]C-36

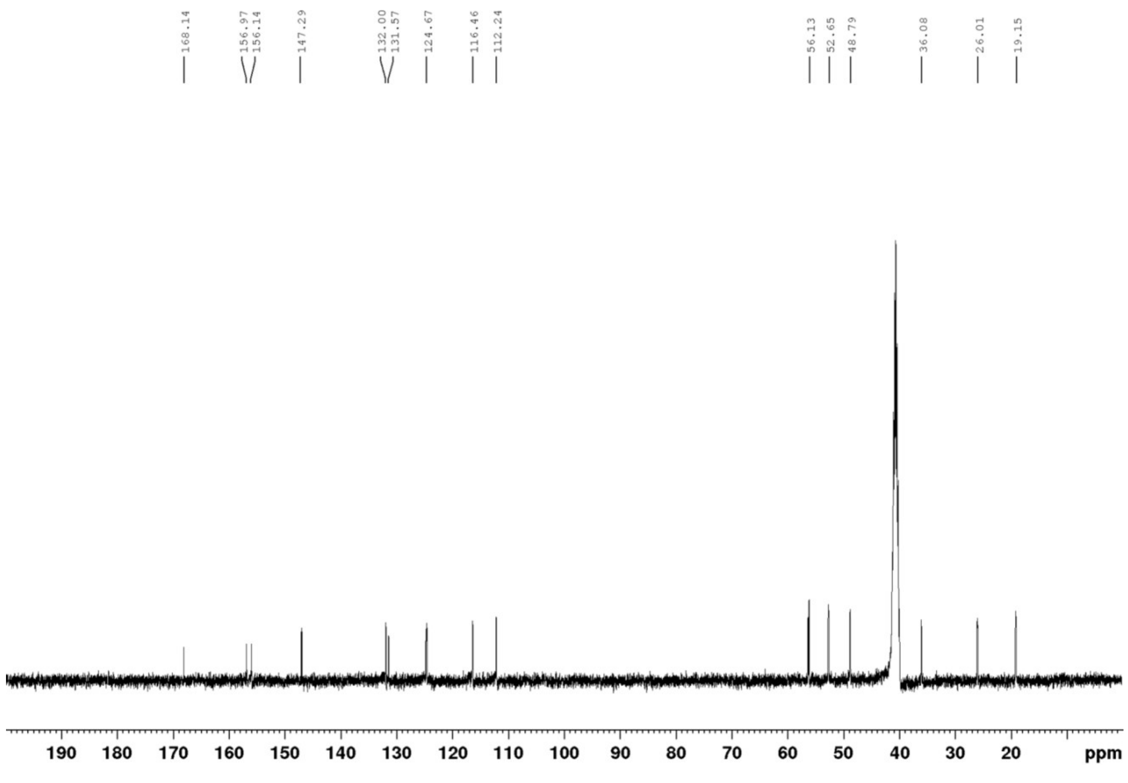
168.14
156.97
156.14
147.29
132.00
131.57
124.67
116.46
112.24
56.13
52.65
48.79
36.08
26.01
19.15
190 180 170 160 150 140 130 120 110 100 90 80 70 60 50 40 30 20 ppm

^{1}H-37

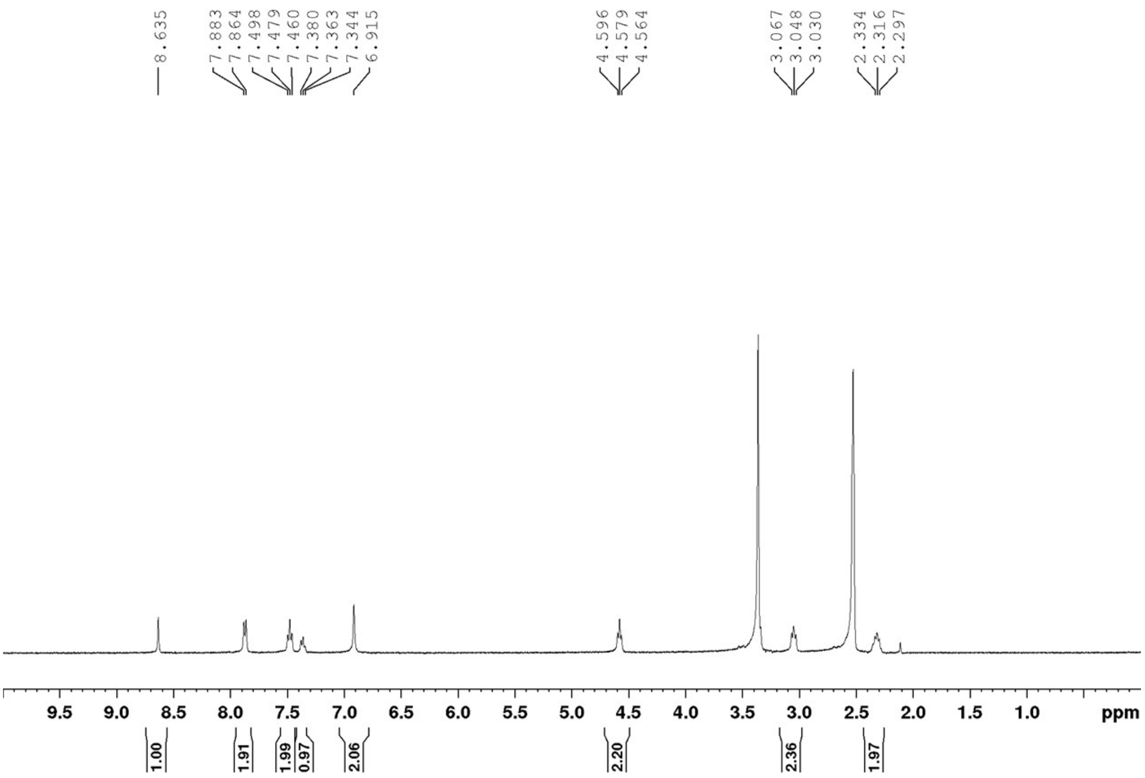
8.635
7.883
7.864
7.498
7.479
7.460
7.380
7.363
7.344
6.915
4.596
4.579
4.564
3.067
3.048
3.030
2.334
2.316
2.297
ppm
1.00
1.91
1.99
0.97
2.06
2.20
2.36
1.97

^{13}C-37

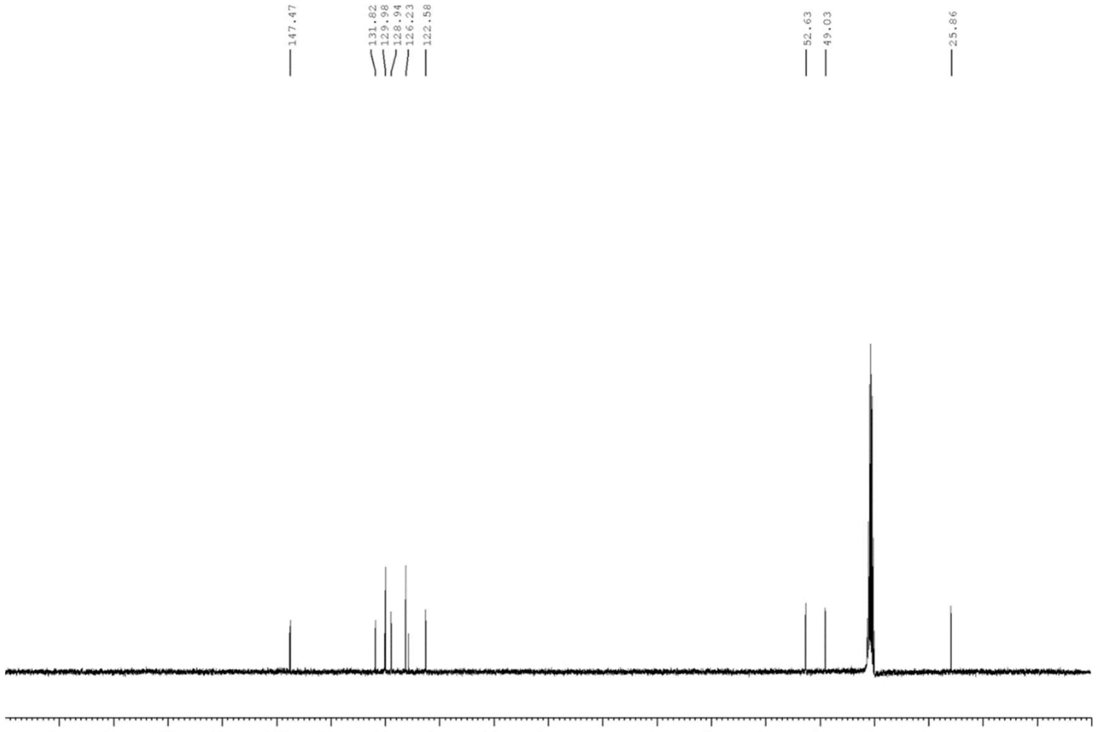
147.47
131.82
129.98
128.94
126.23
122.58
52.63
49.03
25.86
ppm

^{1}H-38

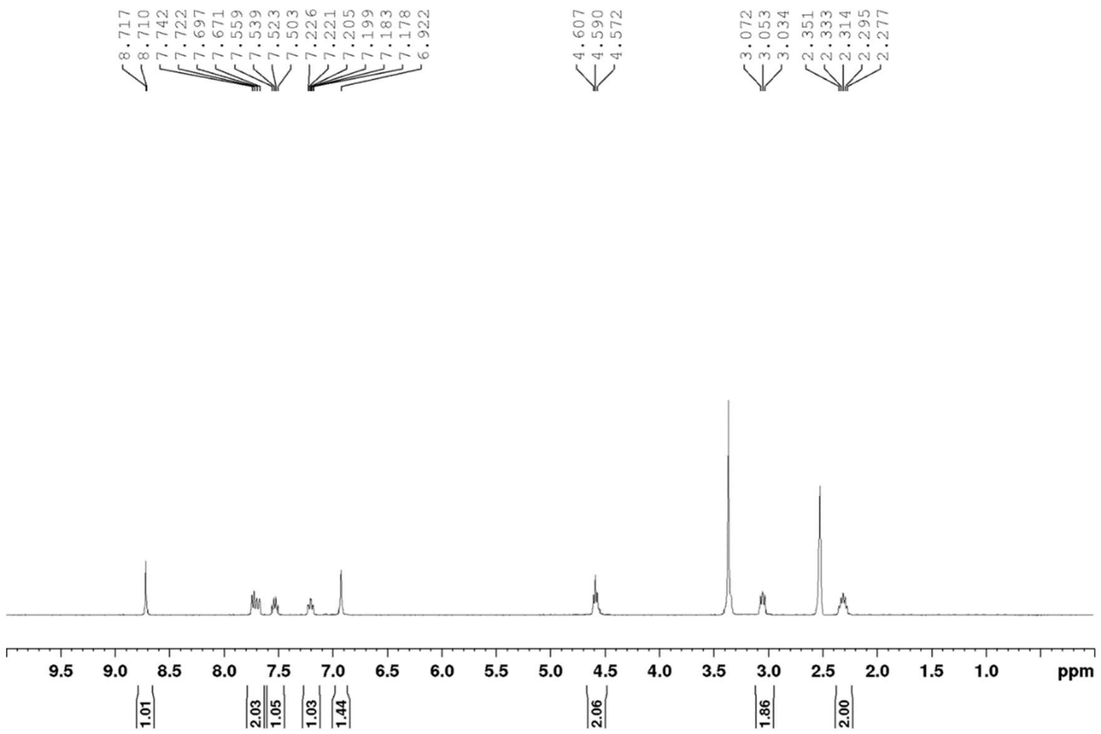

^{13}C-38

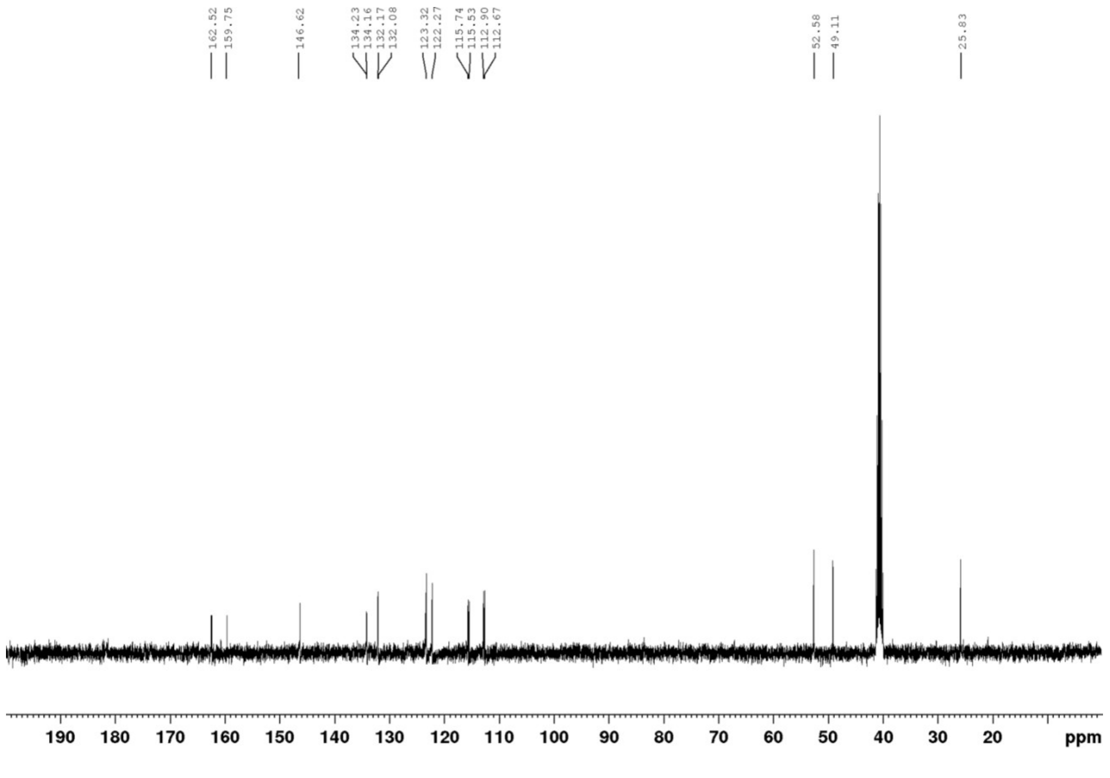

^{1}H-39

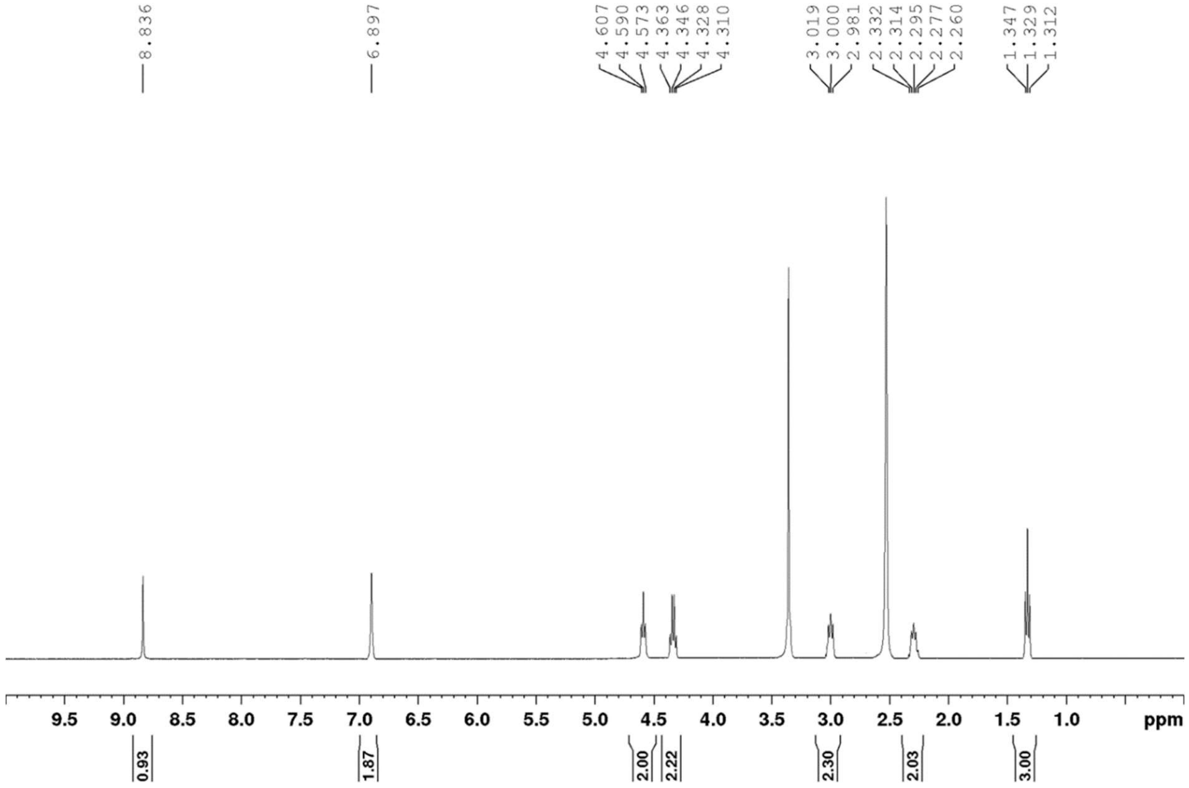
8.836
6.897
4.607
4.590
4.573
4.363
4.346
4.328
4.310
3.019
3.000
2.981
2.332
2.314
2.295
2.277
2.260
1.347
1.329
1.312
9.5 9.0 8.5 8.0 7.5 7.0 6.5 6.0 5.5 5.0 4.5 4.0 3.5 3.0 2.5 2.0 1.5 1.0 ppm
0.93
1.87
2.00
2.22
2.30
2.03
3.00

[13]C-39

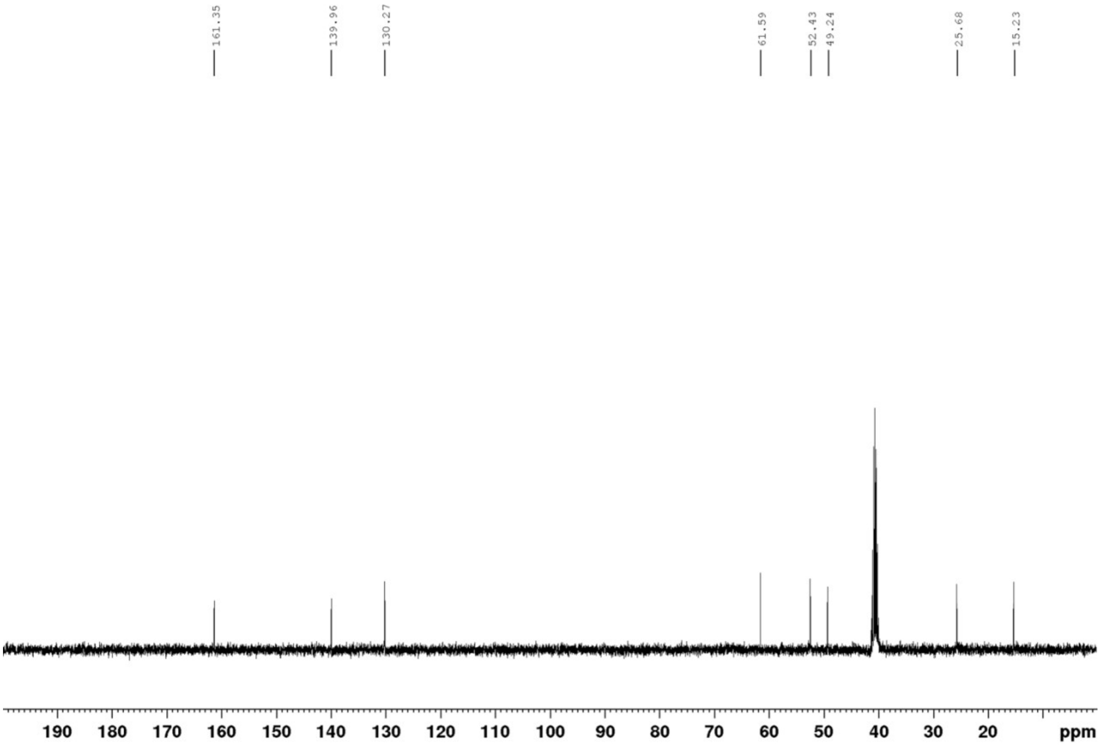
161.35
139.96
130.27
61.59
52.43
49.24
25.68
15.23
190 180 170 160 150 140 130 120 110 100 90 80 70 60 50 40 30 20 ppm

[1]H-40

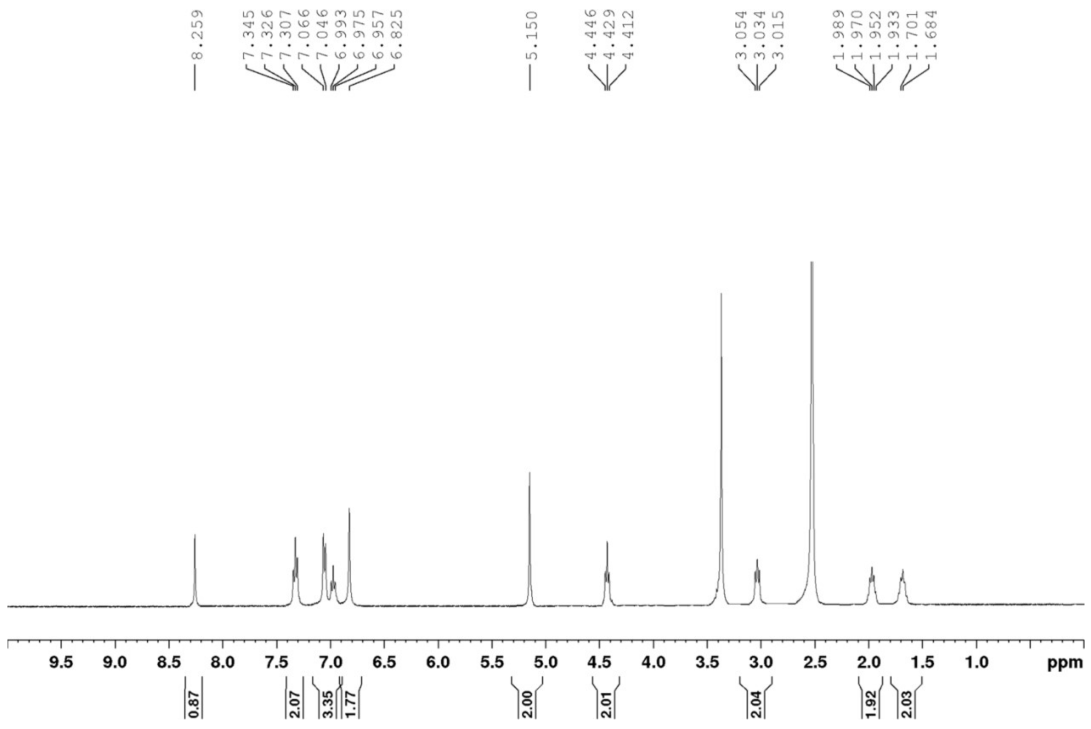
8.259
7.345
7.326
7.307
7.066
7.046
6.993
6.975
6.957
6.825
5.150
4.446
4.429
4.412
3.054
3.034
3.015
1.989
1.970
1.952
1.933
1.701
1.684
9.5 9.0 8.5 8.0 7.5 7.0 6.5 6.0 5.5 5.0 4.5 4.0 3.5 3.0 2.5 2.0 1.5 1.0 ppm
0.87
2.07
3.35
1.77
2.00
2.01
2.04
1.92
2.03

^{13}C-40

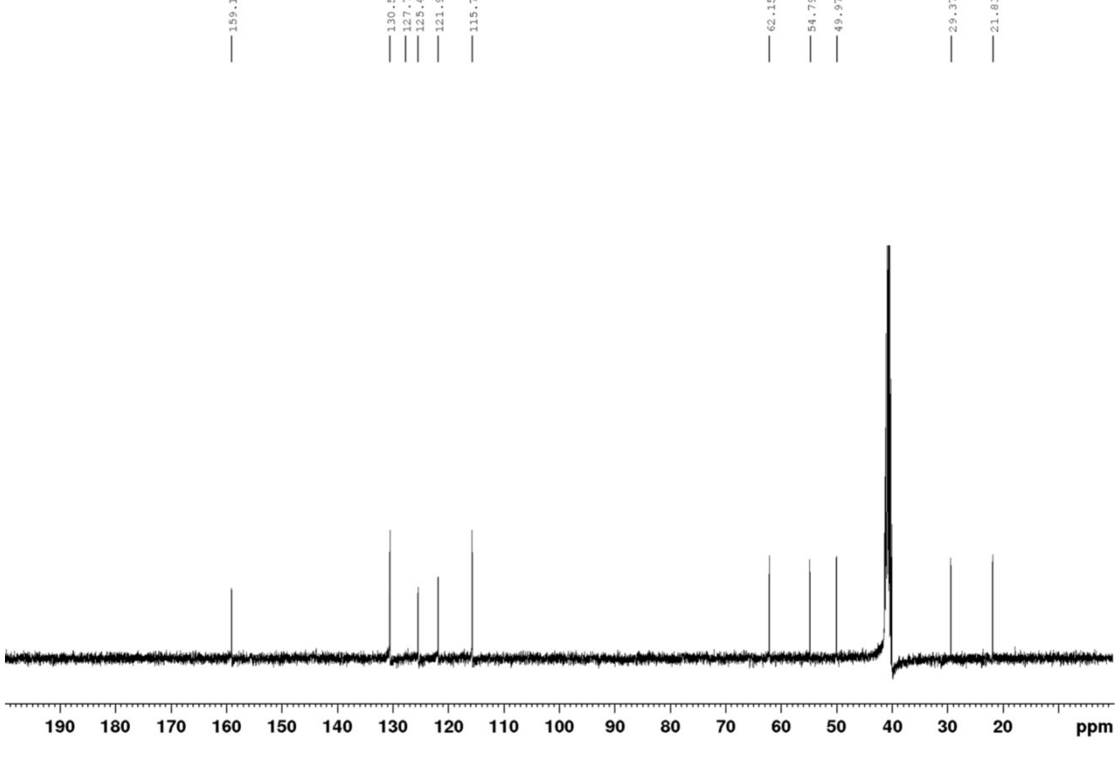
159.15
130.58
127.77
125.46
121.91
115.76
62.15
54.79
49.97
29.37
21.83
190 180 170 160 150 140 130 120 110 100 90 80 70 60 50 40 30 20 ppm

^{1}H-41

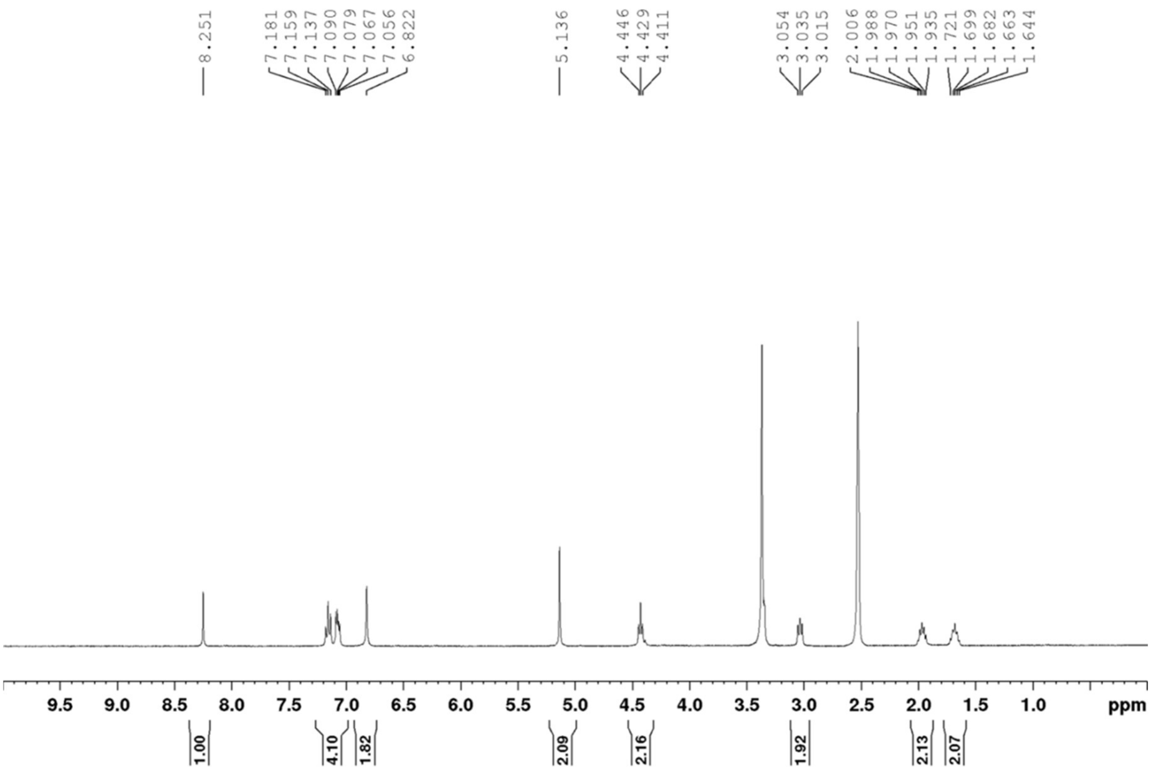
8.251
7.181
7.159
7.137
7.090
7.079
7.067
7.056
6.822
5.136
4.446
4.429
4.411
3.054
3.035
3.015
2.006
1.988
1.970
1.951
1.935
1.721
1.699
1.682
1.663
1.644
9.5 9.0 8.5 8.0 7.5 7.0 6.5 6.0 5.5 5.0 4.5 4.0 3.5 3.0 2.5 2.0 1.5 1.0 ppm
1.00
4.10
1.82
2.09
2.16
1.92
2.13
2.07

^{13}C-41

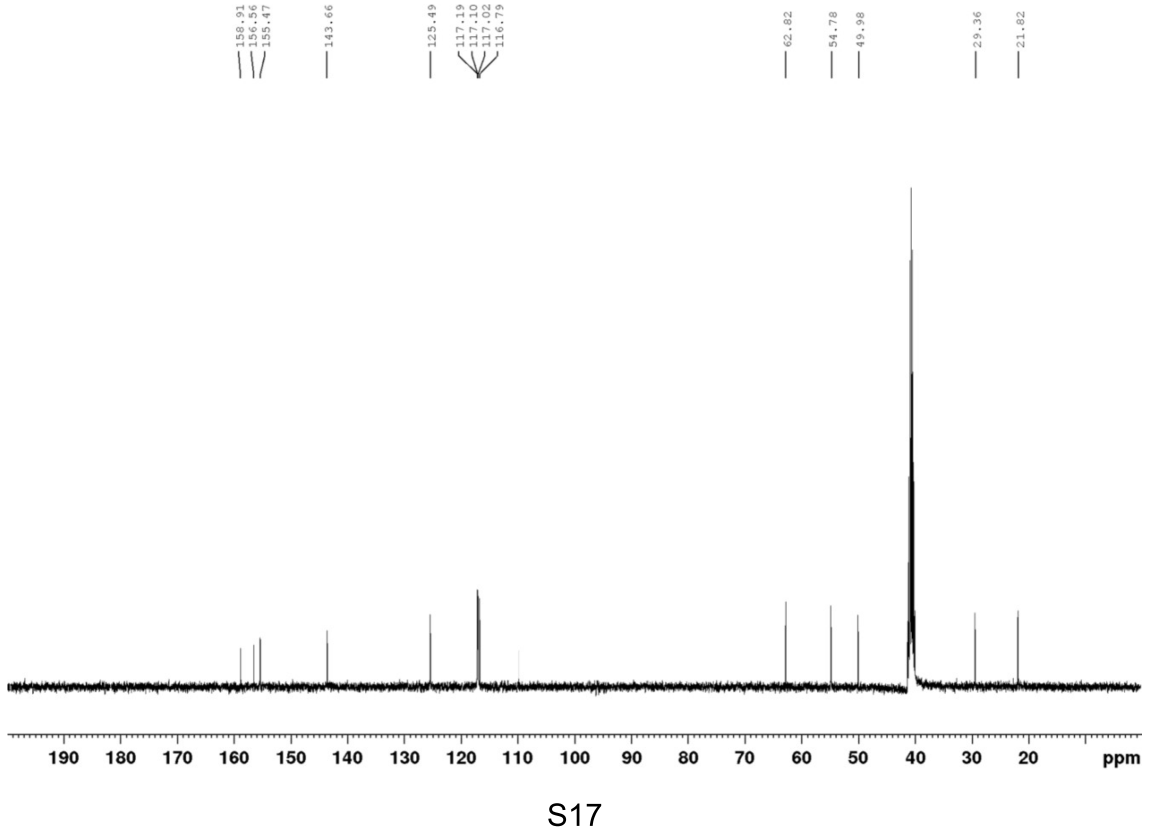
158.91
156.56
155.47
143.66
125.49
117.19
117.10
117.02
116.79
62.82
54.78
49.98
29.36
21.82
190 180 170 160 150 140 130 120 110 100 90 80 70 60 50 40 30 20 ppm

^{1}H-42

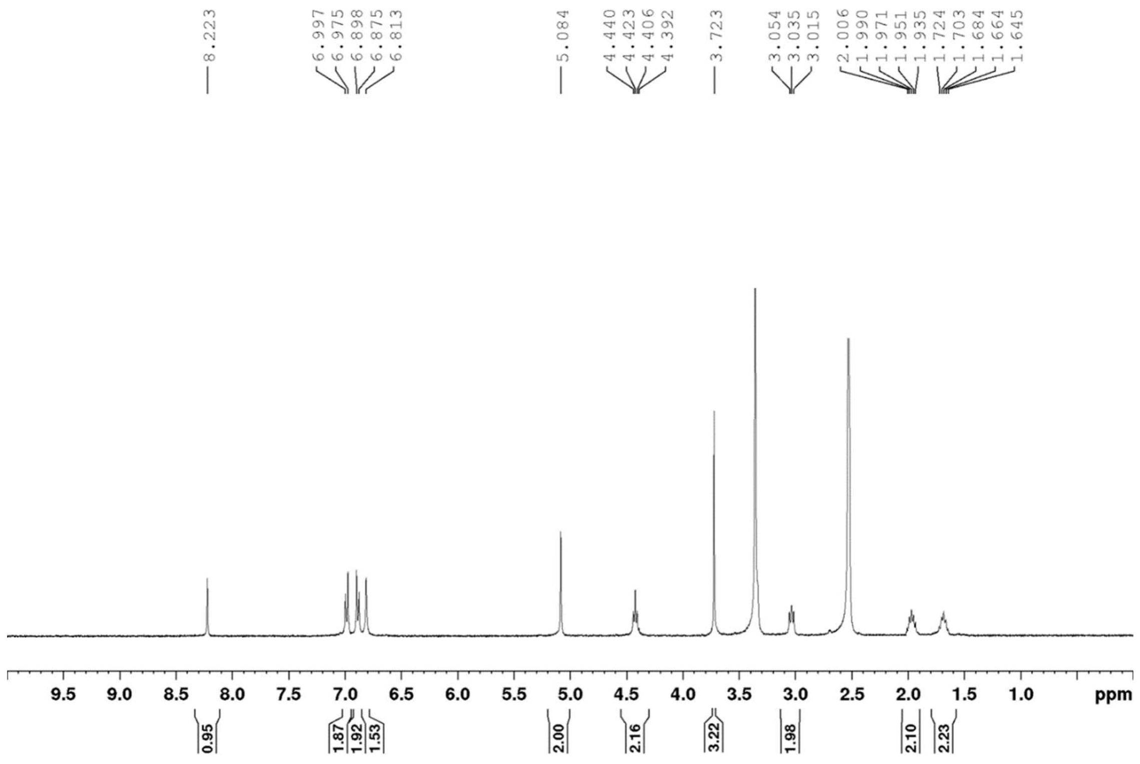
8.223
6.997
6.975
6.898
6.875
6.813
5.084
4.440
4.423
4.406
4.392
3.723
3.054
3.035
3.015
2.006
1.990
1.971
1.951
1.935
1.724
1.703
1.684
1.664
1.645
9.5 9.0 8.5 8.0 7.5 7.0 6.5 6.0 5.5 5.0 4.5 4.0 3.5 3.0 2.5 2.0 1.5 1.0 ppm
0.95
1.87
1.92
1.53
2.00
2.16
3.22
1.98
2.10
2.23

^{13}C-42

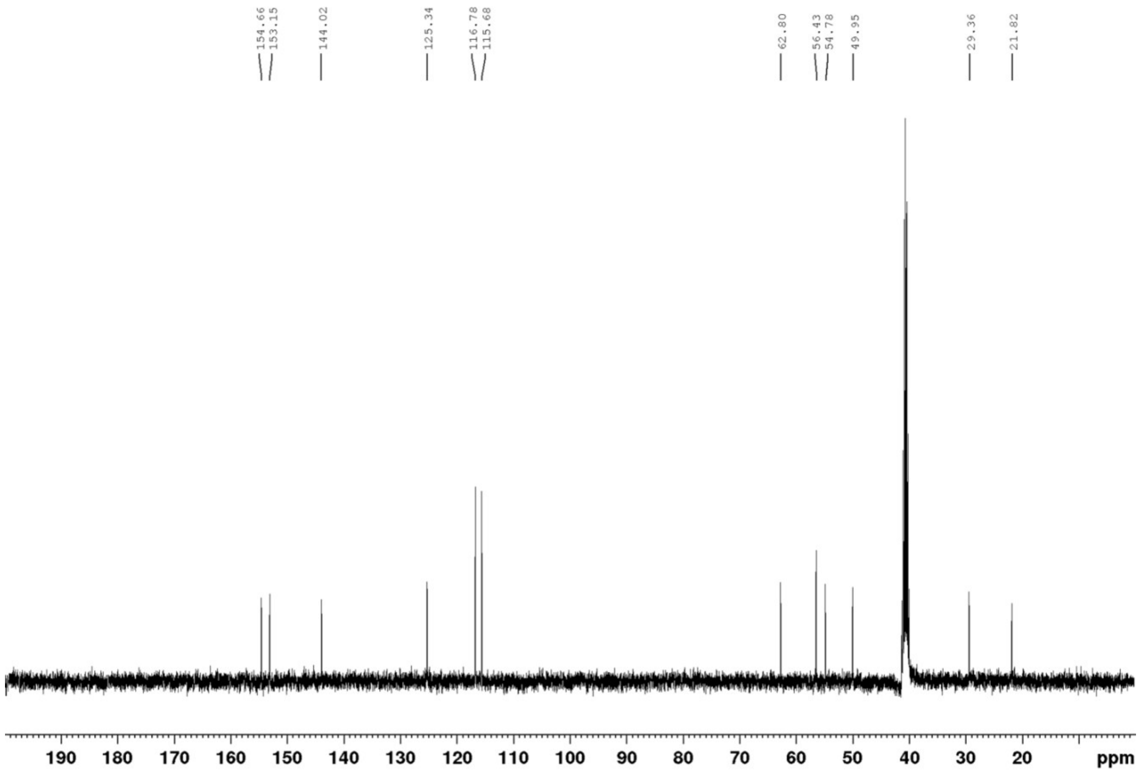
154.66
153.15
144.02
125.34
116.78
115.68
62.80
56.43
54.78
49.95
29.36
21.82
190 180 170 160 150 140 130 120 110 100 90 80 70 60 50 40 30 20 ppm

^{1}H-43

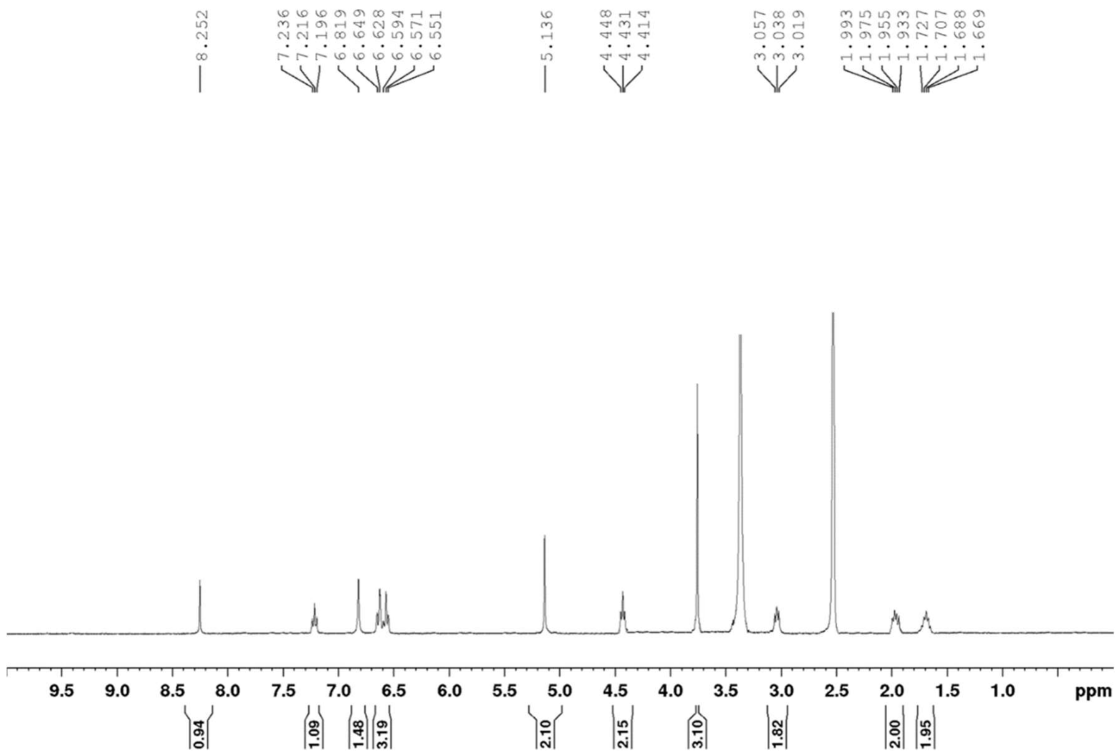
8.252
7.236
7.216
7.196
6.819
6.649
6.628
6.594
6.571
6.551
5.136
4.448
4.431
4.414
3.057
3.038
3.019
1.993
1.975
1.955
1.933
1.727
1.707
1.688
1.669
9.5 9.0 8.5 8.0 7.5 7.0 6.5 6.0 5.5 5.0 4.5 4.0 3.5 3.0 2.5 2.0 1.5 1.0 ppm
0.94
1.09
1.48
3.19
2.10
2.15
3.10
1.82
2.00
1.95

^{13}C-43

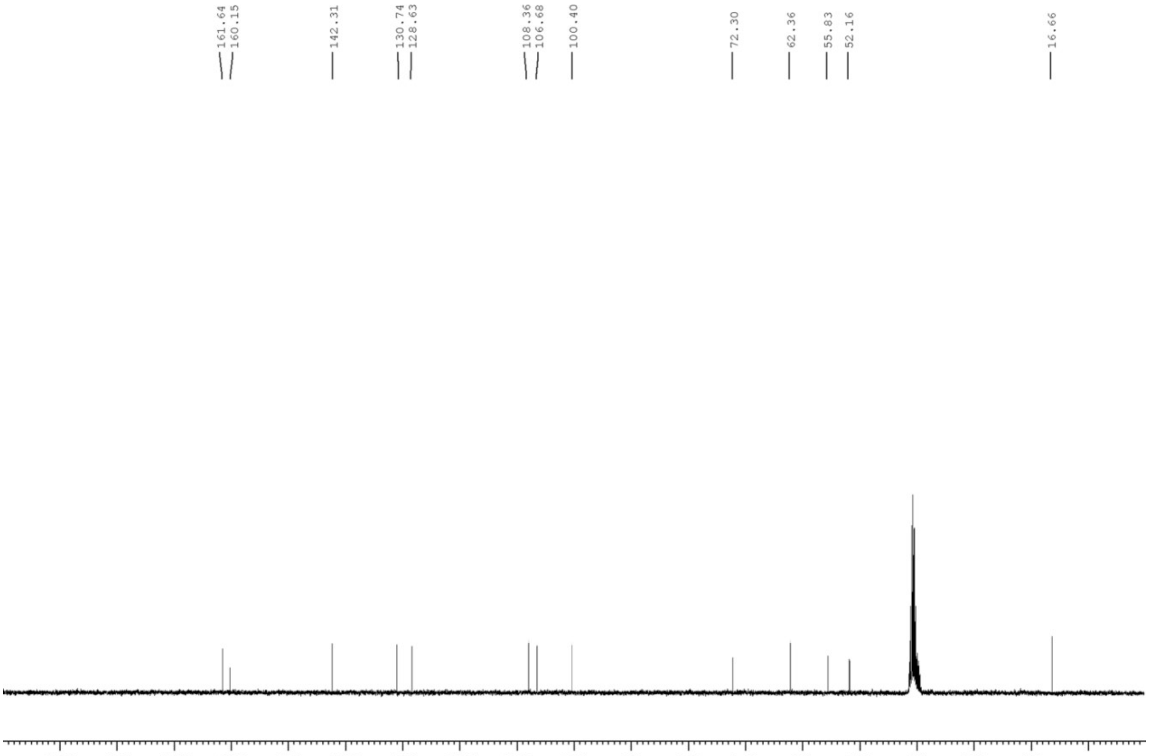
161.64
160.15
142.31
130.74
128.63
108.36
106.68
100.40
72.30
62.36
55.83
52.16
16.66
190 180 170 160 150 140 130 120 110 100 90 80 70 60 50 40 30 20 ppm

^{1}H-44

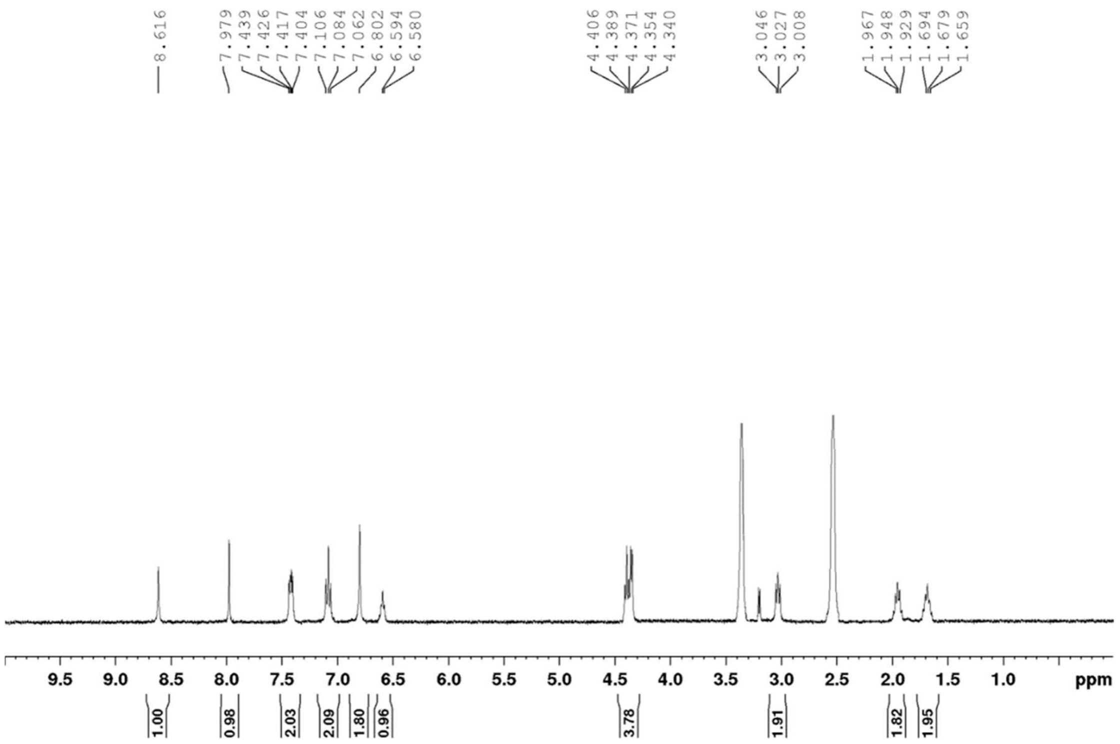
8.616
7.979
7.439
7.426
7.417
7.404
7.106
7.084
7.062
6.802
6.594
6.580
4.406
4.389
4.371
4.354
4.340
3.046
3.027
3.008
1.967
1.948
1.929
1.694
1.679
1.659
9.5 9.0 8.5 8.0 7.5 7.0 6.5 6.0 5.5 5.0 4.5 4.0 3.5 3.0 2.5 2.0 1.5 1.0 ppm
1.00
0.98
2.03
2.09
1.80
0.96
3.78
1.91
1.82
1.95

^{13}C-44

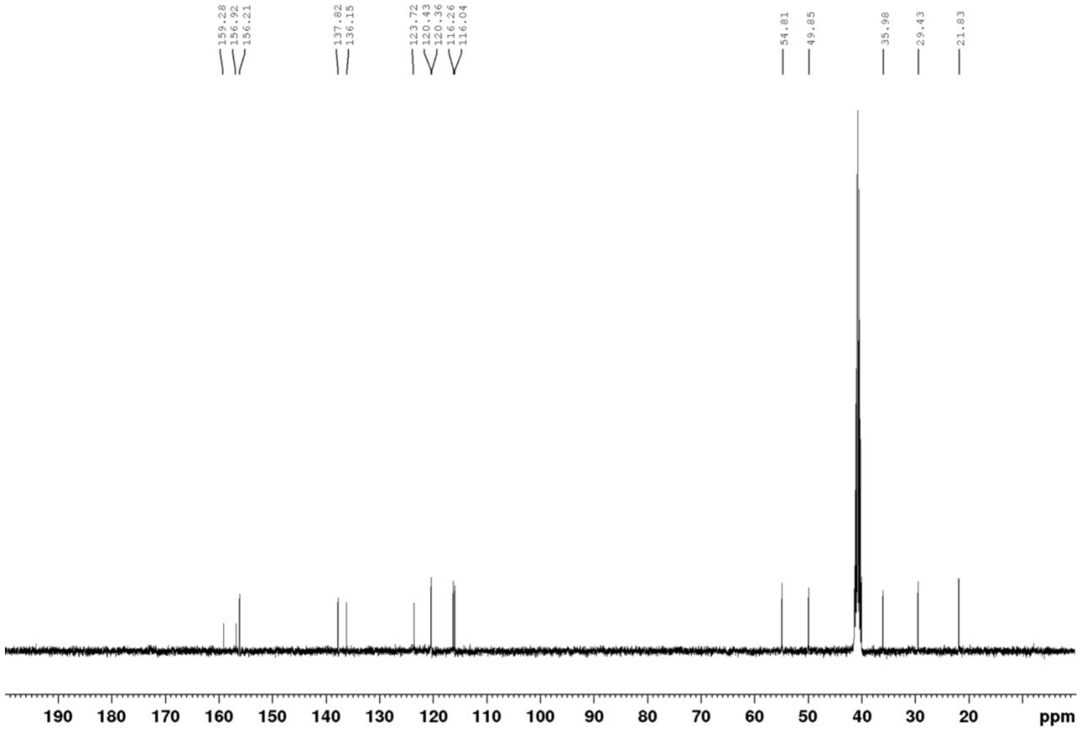
159.28
156.92
156.21
137.82
136.15
123.72
120.43
120.36
116.26
116.04
54.81
49.85
35.98
29.43
21.83
190 180 170 160 150 140 130 120 110 100 90 80 70 60 50 40 30 20 ppm

[1]H-45

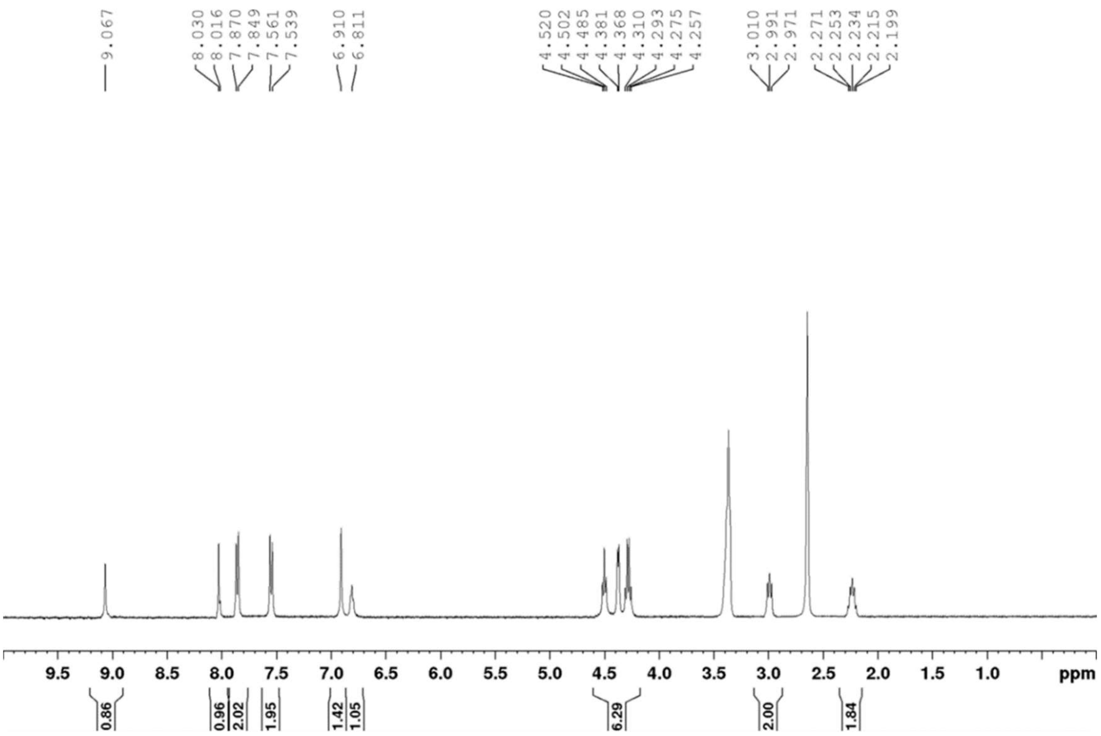
9.067
8.030
8.016
7.870
7.849
7.561
7.539
6.910
6.811
4.520
4.502
4.485
4.381
4.368
4.310
4.293
4.275
4.257
3.010
2.991
2.971
2.271
2.253
2.234
2.215
2.199
9.5 9.0 8.5 8.0 7.5 7.0 6.5 6.0 5.5 5.0 4.5 4.0 3.5 3.0 2.5 2.0 1.5 1.0 ppm
0.86
0.96
2.02
1.95
1.42
1.05
6.29
2.00
1.84

[13]C-45

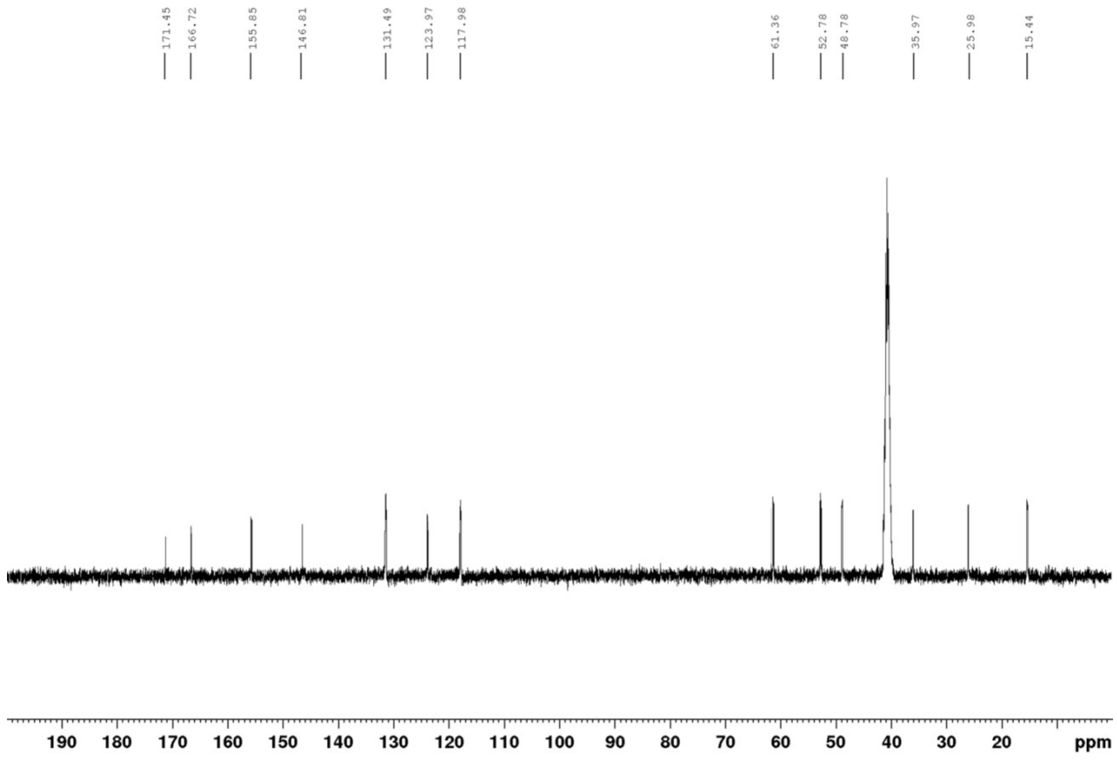
171.45
166.72
155.85
146.81
131.49
123.97
117.98
61.36
52.78
48.78
35.97
25.98
15.44
190 180 170 160 150 140 130 120 110 100 90 80 70 60 50 40 30 20 ppm

^{1}H-46

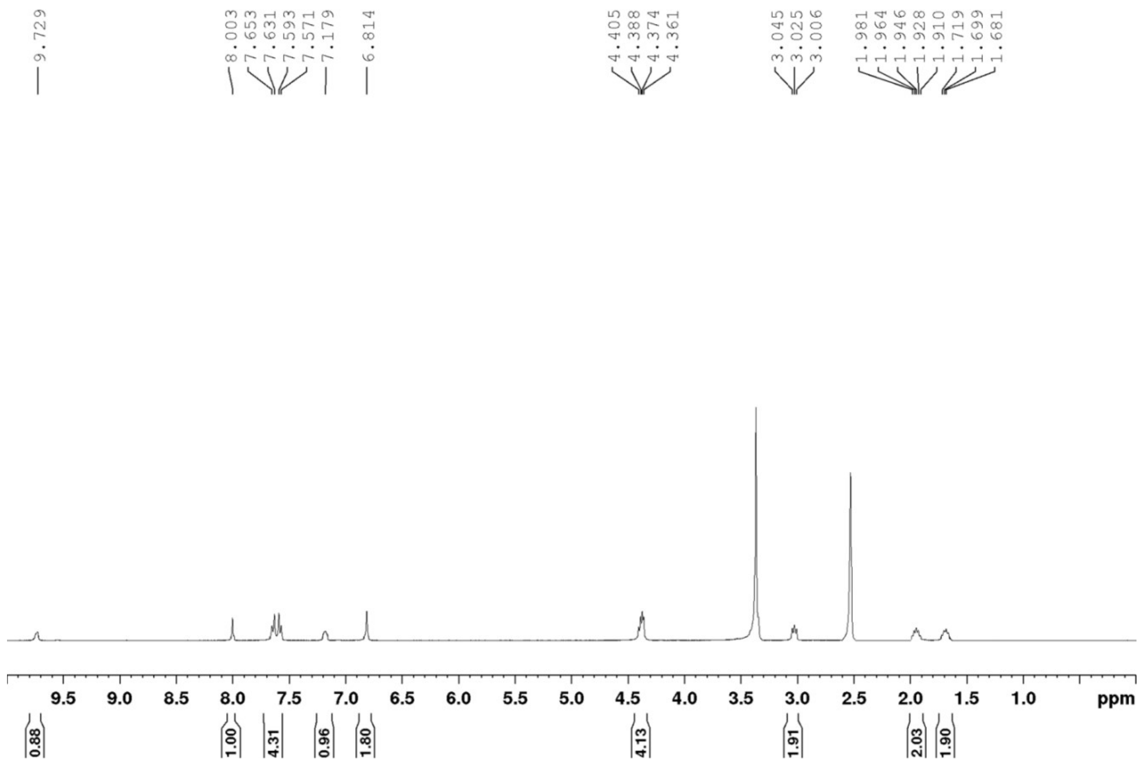
9.729
8.003
7.653
7.631
7.593
7.571
7.179
6.814
4.405
4.388
4.374
4.361
3.045
3.025
3.006
1.981
1.964
1.946
1.928
1.910
1.719
1.699
1.681
9.5 9.0 8.5 8.0 7.5 7.0 6.5 6.0 5.5 5.0 4.5 4.0 3.5 3.0 2.5 2.0 1.5 1.0 ppm
0.88
1.00
4.31
0.96
1.80
4.13
1.91
2.03
1.90

^{13}C-46

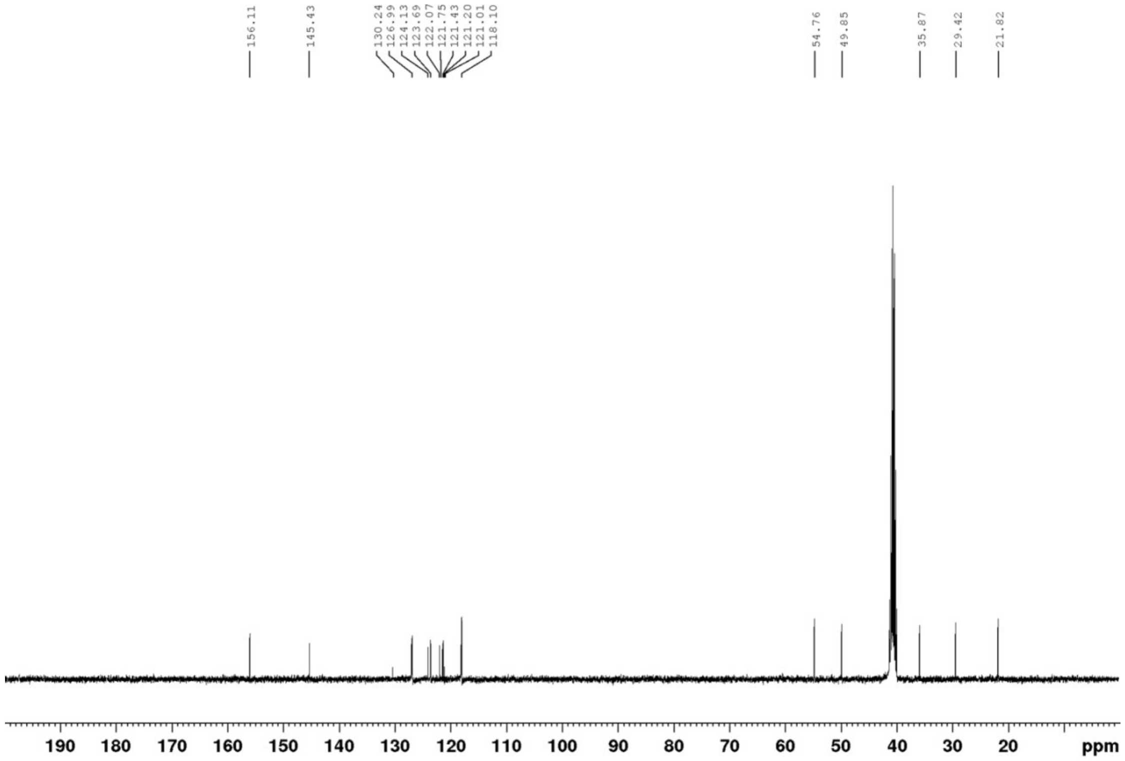
156.11
145.43
130.24
126.99
124.13
123.69
122.07
121.75
121.43
121.20
121.01
118.10
54.76
49.85
35.87
29.42
21.82
190 180 170 160 150 140 130 120 110 100 90 80 70 60 50 40 30 20 ppm

[1]H-47

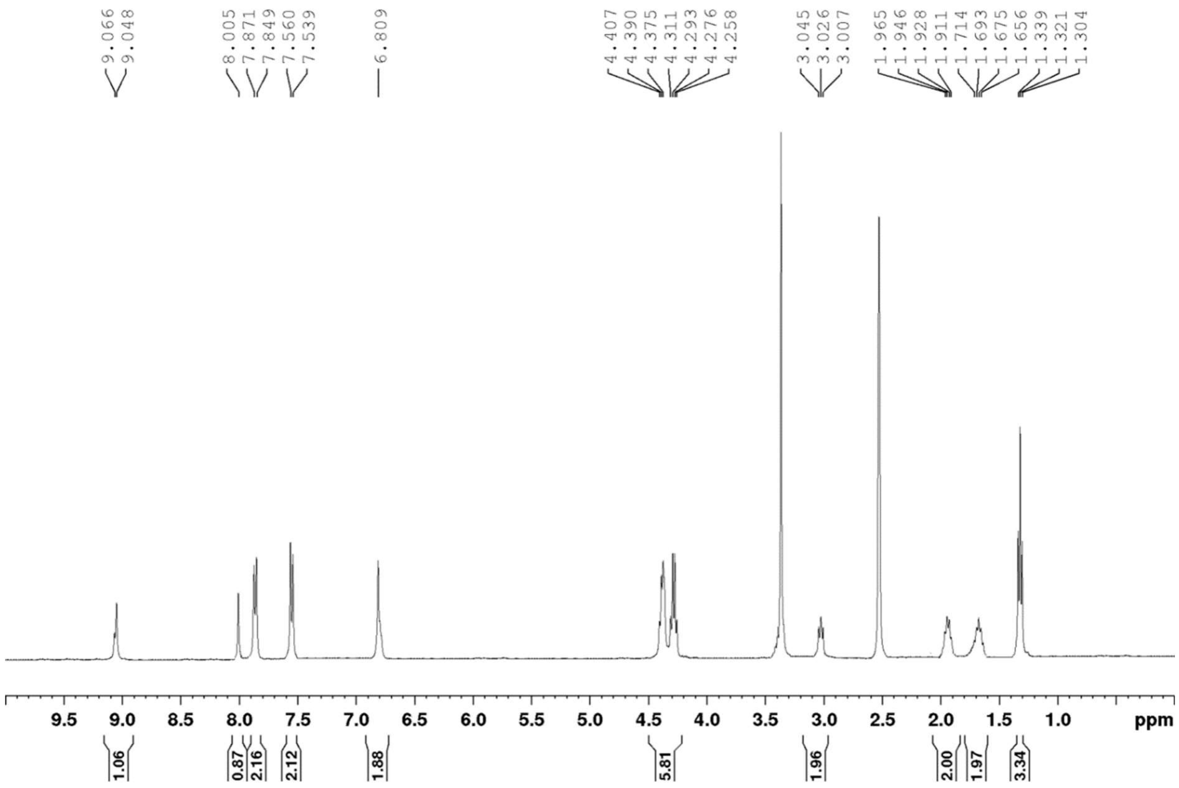
9.066
9.048
8.005
7.871
7.849
7.560
7.539
6.809
4.407
4.390
4.375
4.311
4.293
4.276
4.258
3.045
3.026
3.007
1.965
1.946
1.928
1.911
1.714
1.693
1.675
1.656
1.339
1.321
1.304
9.5 9.0 8.5 8.0 7.5 7.0 6.5 6.0 5.5 5.0 4.5 4.0 3.5 3.0 2.5 2.0 1.5 1.0 ppm
1.06
0.87
2.16
2.12
1.88
5.81
1.96
2.00
1.97
3.34

[13]C-47

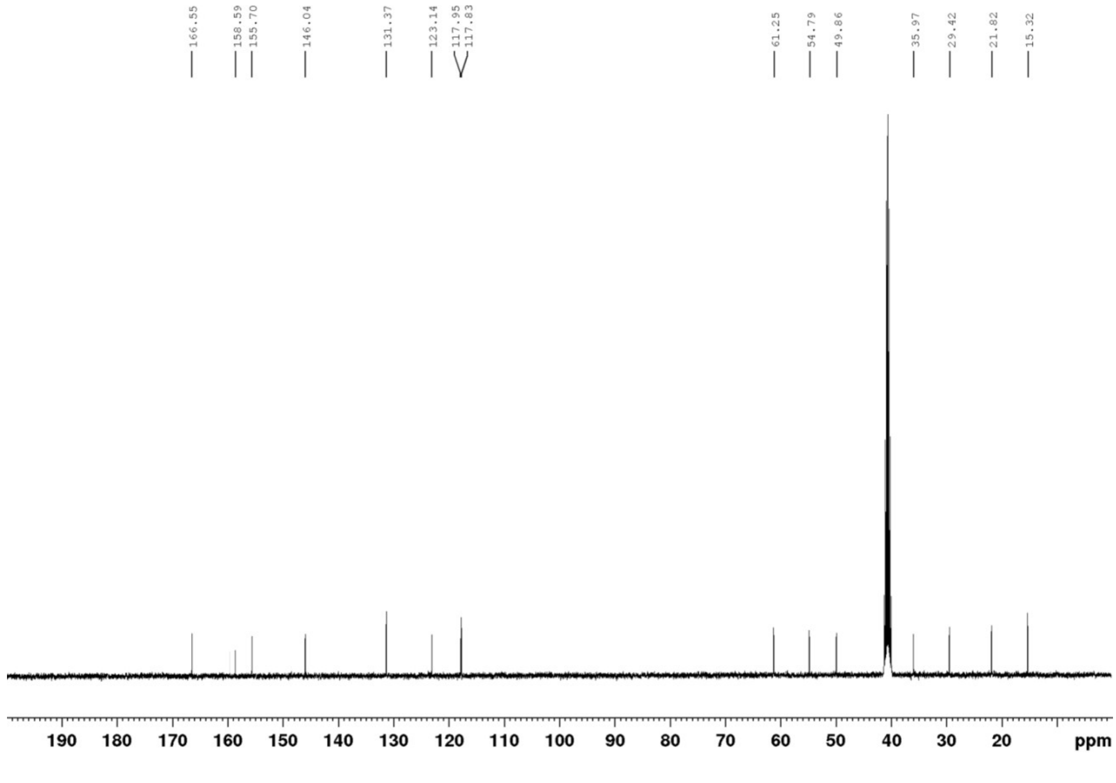
166.55
158.59
155.70
146.04
131.37
123.14
117.95
117.83
61.25
54.79
49.86
35.97
29.42
21.82
15.32
190 180 170 160 150 140 130 120 110 100 90 80 70 60 50 40 30 20 ppm

^{1}H-48

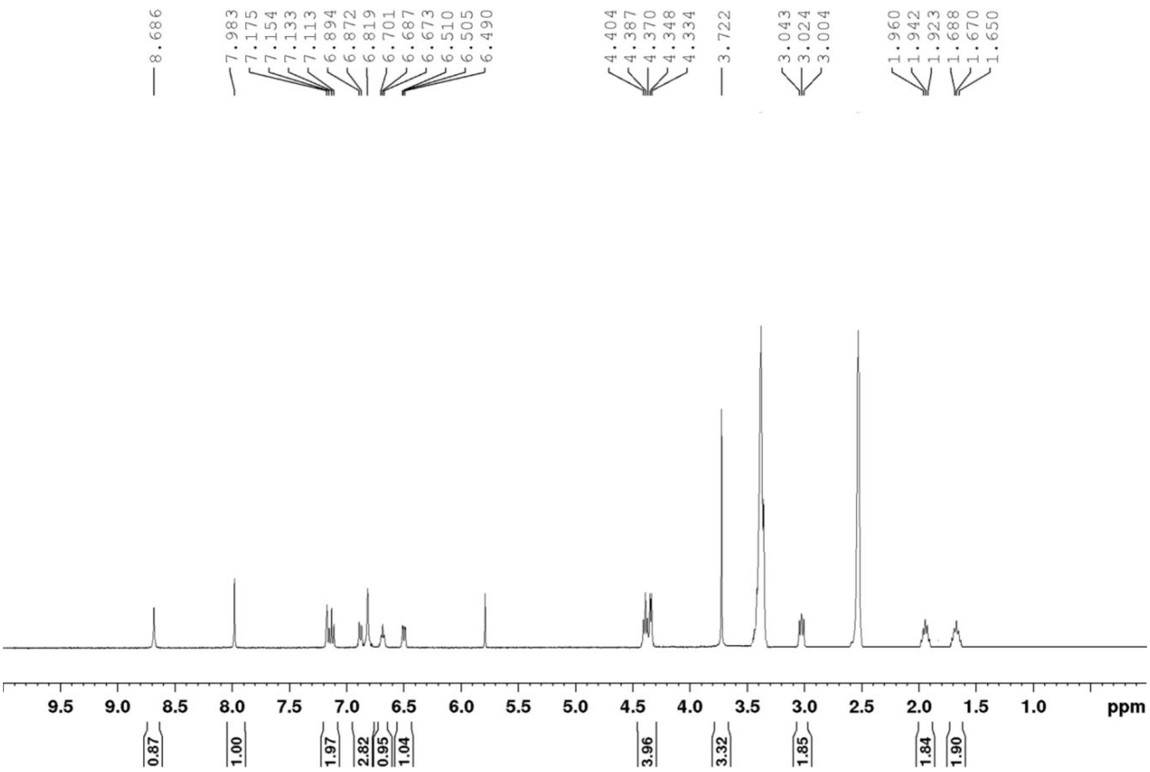
8.686
7.983
7.175
7.154
7.133
7.113
6.894
6.872
6.819
6.701
6.687
6.673
6.510
6.505
6.490
4.404
4.387
4.370
4.348
4.334
3.722
3.043
3.024
3.004
1.960
1.942
1.923
1.688
1.670
1.650
9.5 9.0 8.5 8.0 7.5 7.0 6.5 6.0 5.5 5.0 4.5 4.0 3.5 3.0 2.5 2.0 1.5 1.0 ppm
0.87
1.00
1.97
2.82
0.95
1.04
3.96
3.32
1.85
1.84
1.90

^{13}C-48

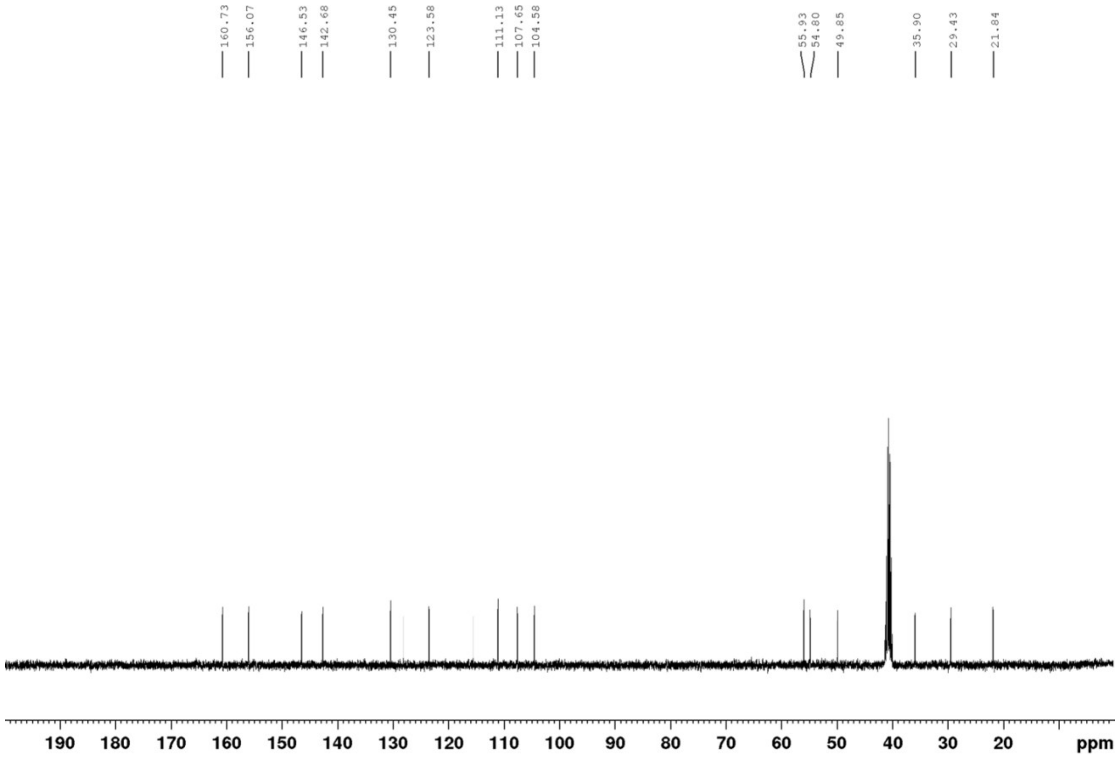
160.73
156.07
146.53
142.68
130.45
123.58
111.13
107.65
104.58
55.93
54.80
49.85
35.90
29.43
21.84
190 180 170 160 150 140 130 120 110 100 90 80 70 60 50 40 30 20 ppm

^{1}H-49

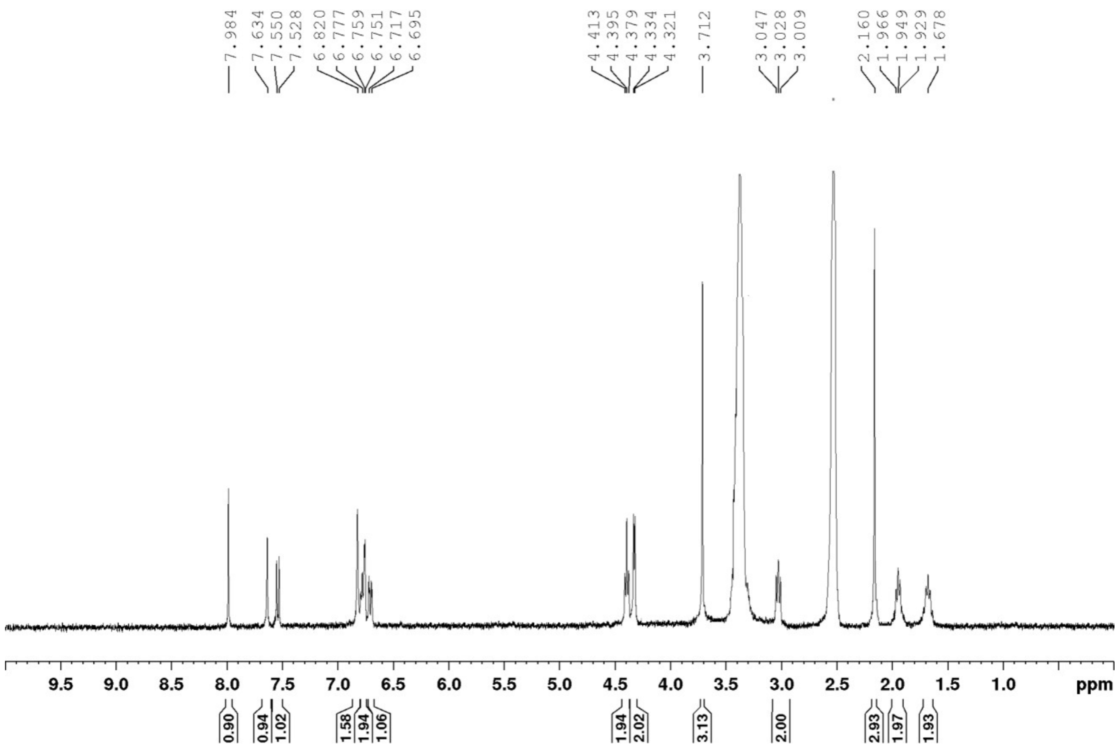
7.984
7.634
7.550
7.528
6.820
6.777
6.759
6.751
6.717
6.695
4.413
4.395
4.379
4.334
4.321
3.712
3.047
3.028
3.009
2.160
1.966
1.949
1.929
1.678
9.5
9.0
8.5
8.0
7.5
7.0
6.5
6.0
5.5
5.0
4.5
4.0
3.5
3.0
2.5
2.0
1.5
1.0
ppm
0.90
0.94
1.02
1.58
1.94
1.06
1.94
2.02
3.13
2.00
2.93
1.97
1.93

^{13}C-49

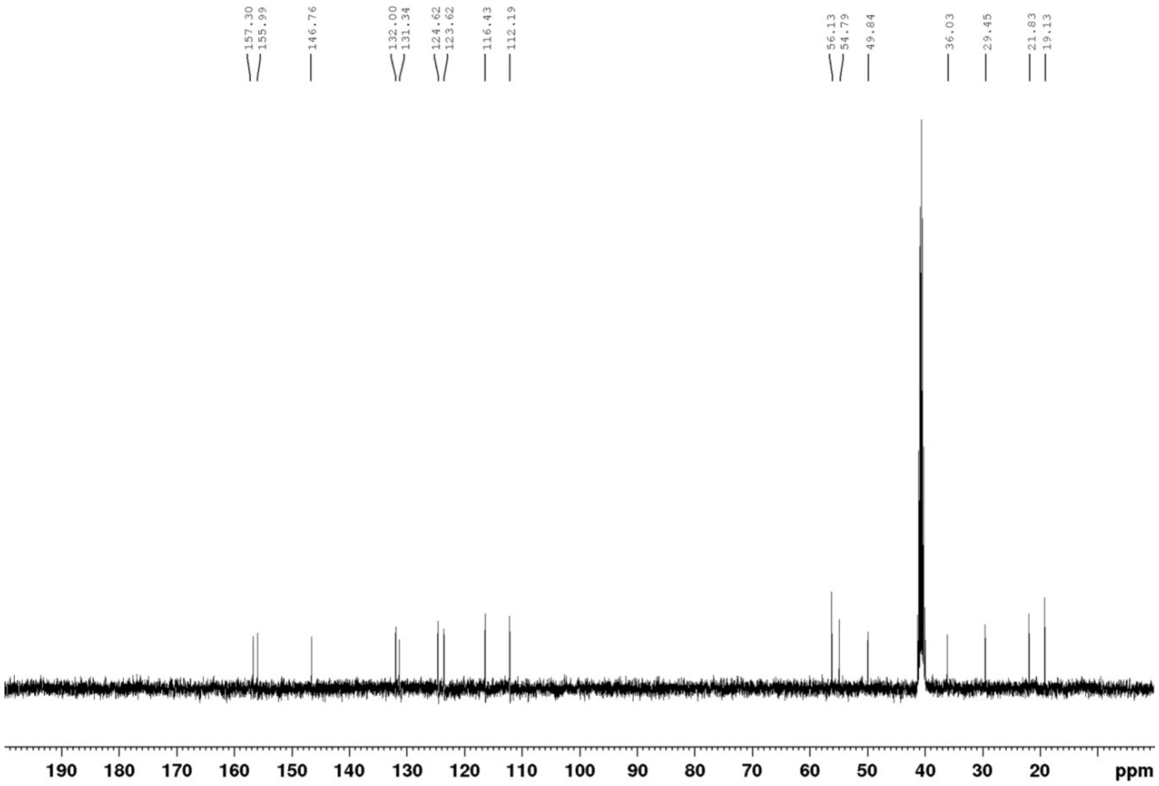
157.30
155.99
146.76
132.00
131.34
124.62
123.62
116.43
112.19
56.13
54.79
49.84
36.03
29.45
21.83
19.13
190
180
170
160
150
140
130
120
110
100
90
80
70
60
50
40
30
20
ppm

^{1}H-50

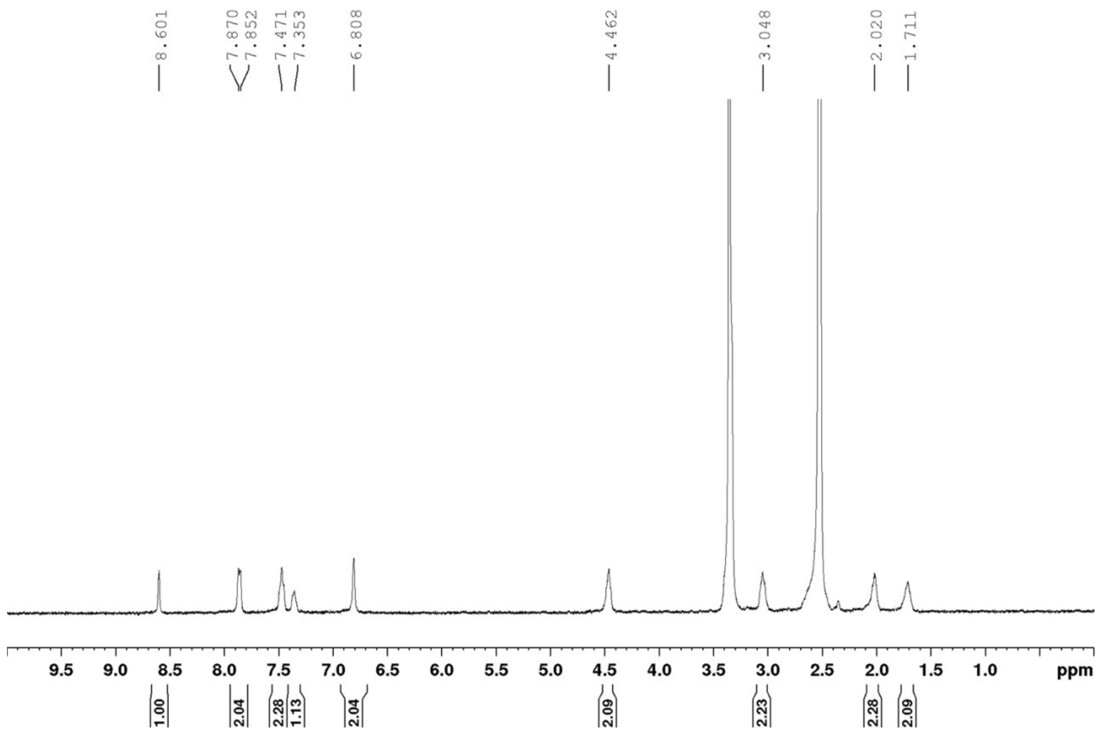
8.601
7.870
7.852
7.471
7.353
6.808
4.462
3.048
2.020
1.711
9.5 9.0 8.5 8.0 7.5 7.0 6.5 6.0 5.5 5.0 4.5 4.0 3.5 3.0 2.5 2.0 1.5 1.0 ppm
1.00
2.04
2.28
1.13
2.04
2.09
2.23
2.28
2.09

^{13}C-50

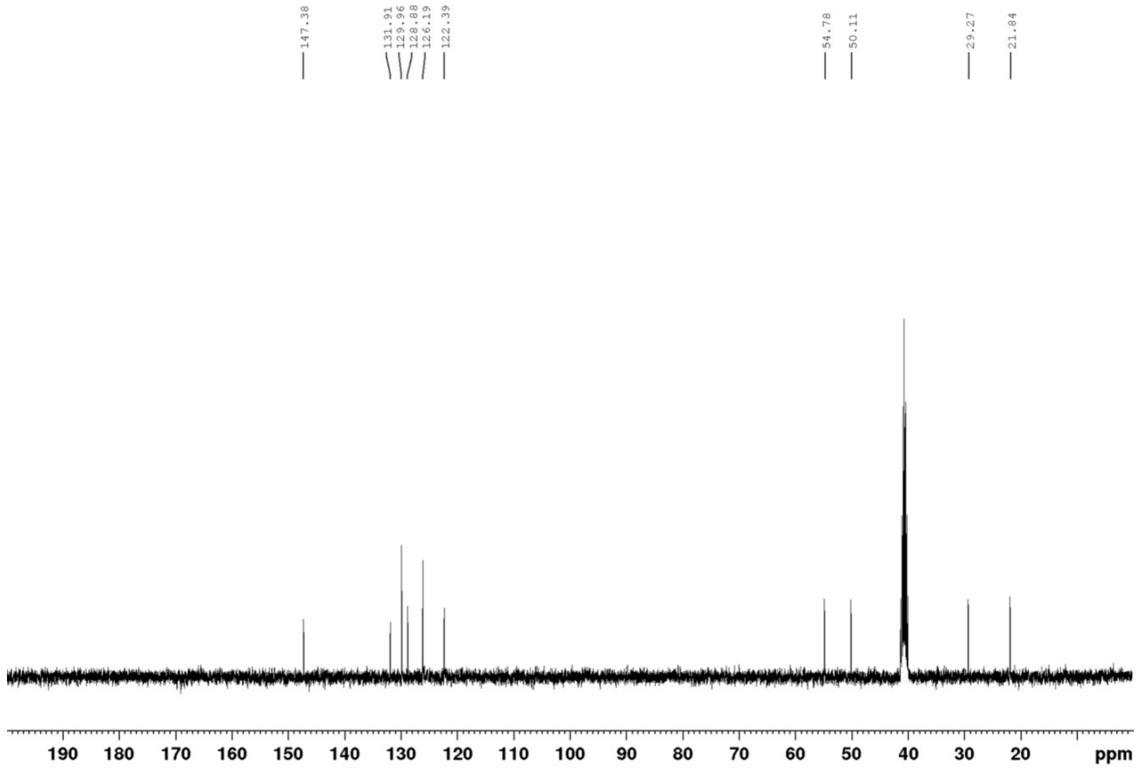
147.38
131.91
129.96
128.88
126.19
122.39
54.78
50.11
29.27
21.84
190 180 170 160 150 140 130 120 110 100 90 80 70 60 50 40 30 20 ppm

^{1}H-51

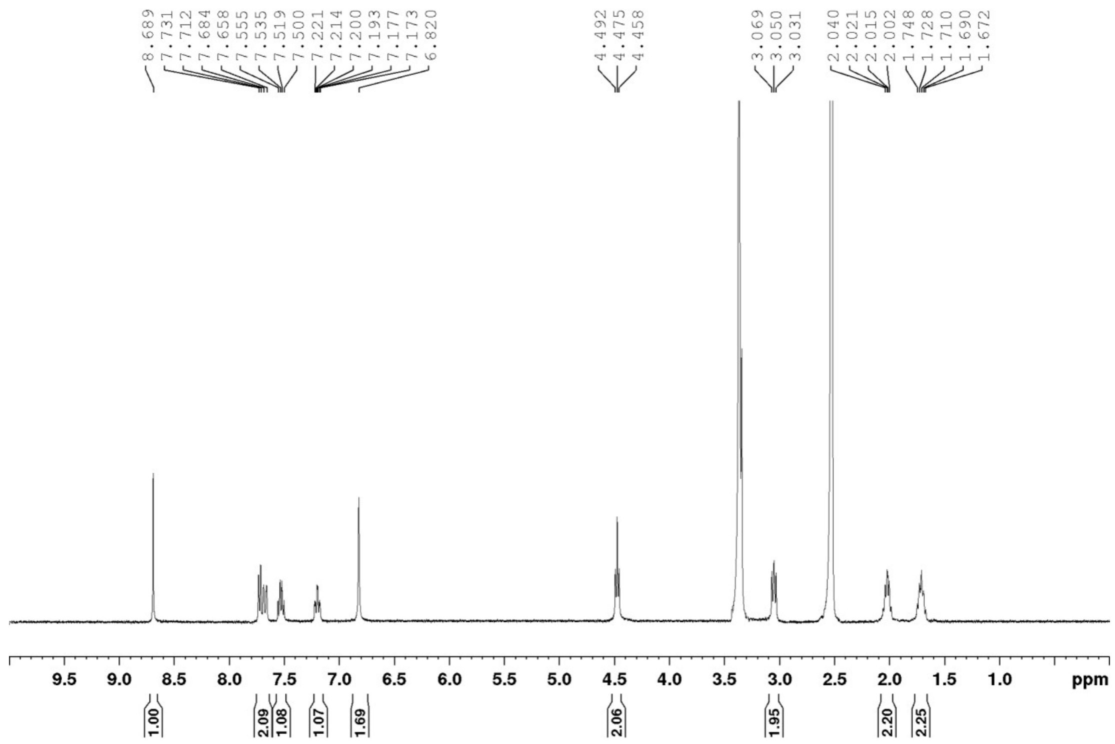

^{13}C-51

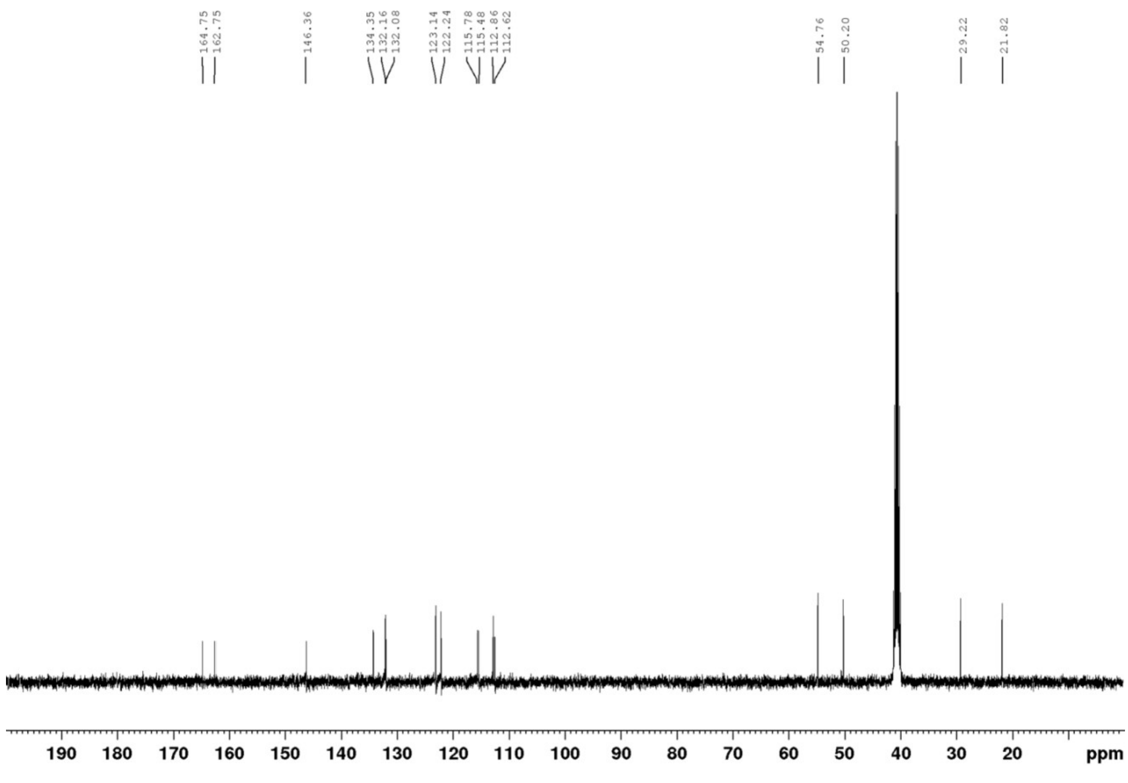

^{1}H-52

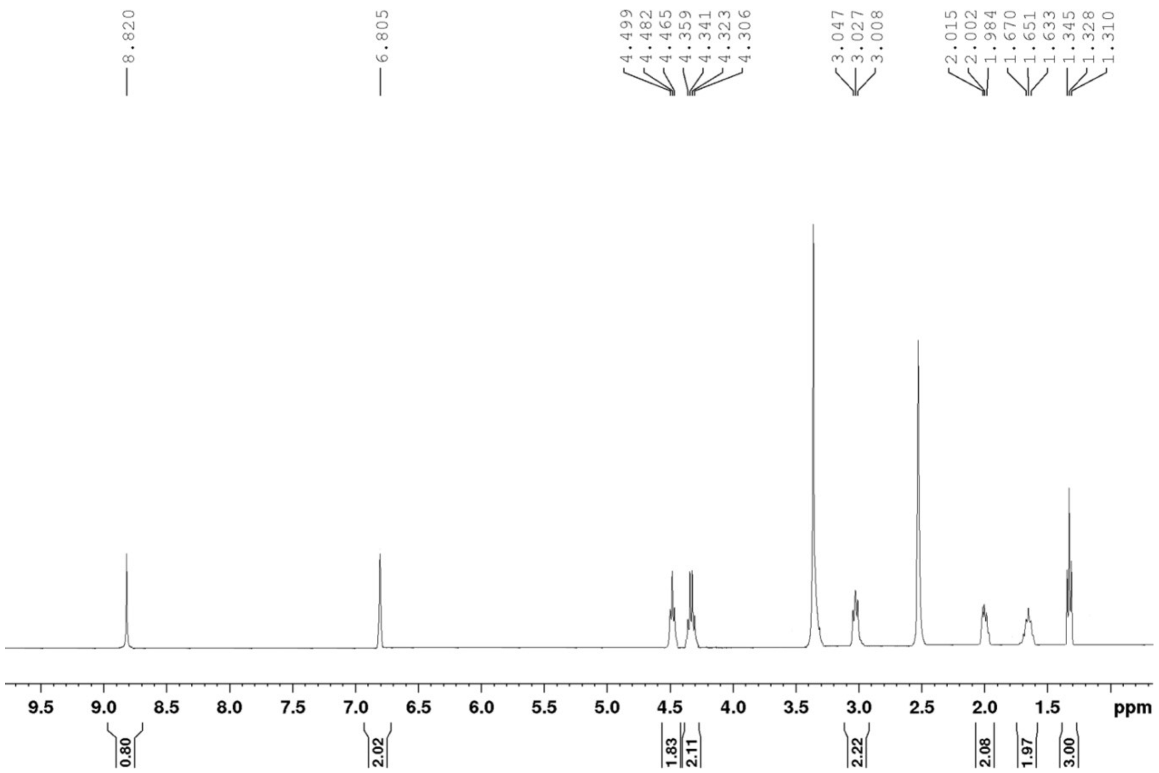
8.820
6.805
4.499
4.482
4.465
4.359
4.341
4.323
4.306
3.047
3.027
3.008
2.015
2.002
1.984
1.670
1.651
1.633
1.345
1.328
1.310
9.5
9.0
8.5
8.0
7.5
7.0
6.5
6.0
5.5
5.0
4.5
4.0
3.5
3.0
2.5
2.0
1.5
ppm
0.80
2.02
1.83
2.11
2.22
2.08
1.97
3.00

^{13}C-52

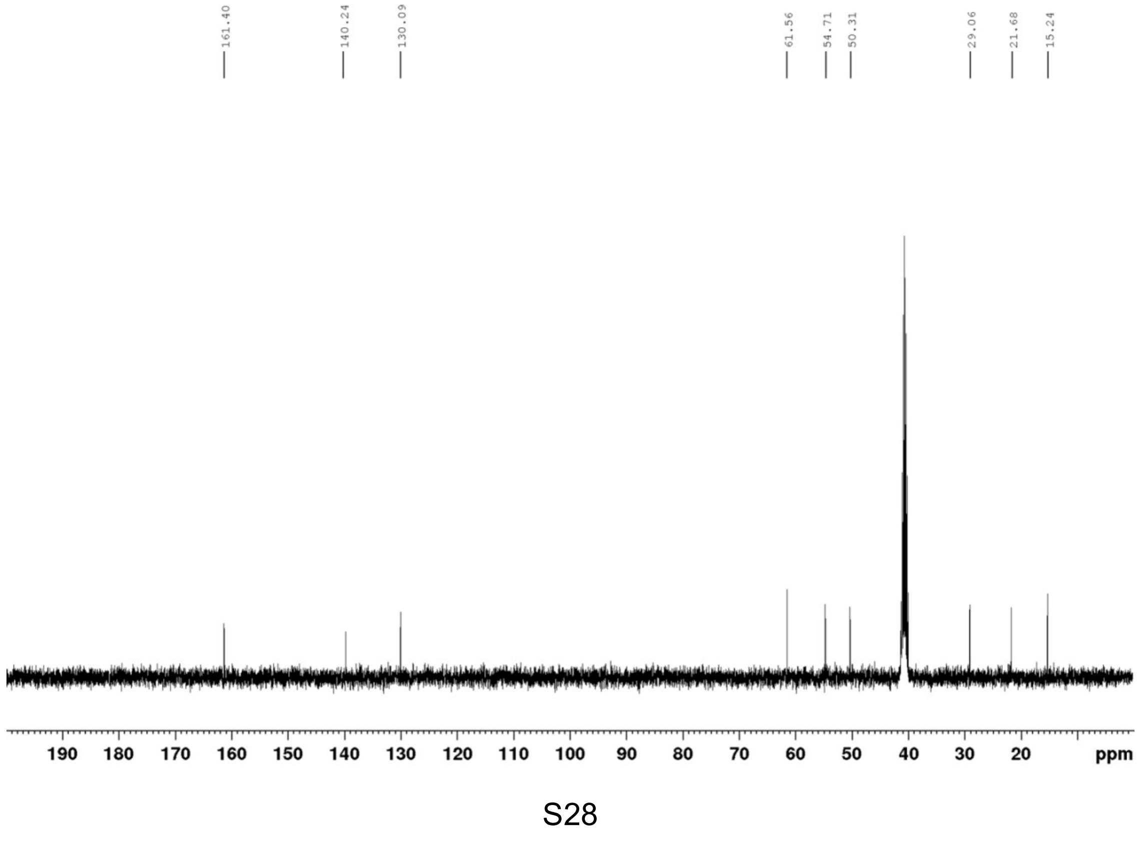
161.40
140.24
130.09
61.56
54.71
50.31
29.06
21.68
15.24
190
180
170
160
150
140
130
120
110
100
90
80
70
60
50
40
30
20
ppm

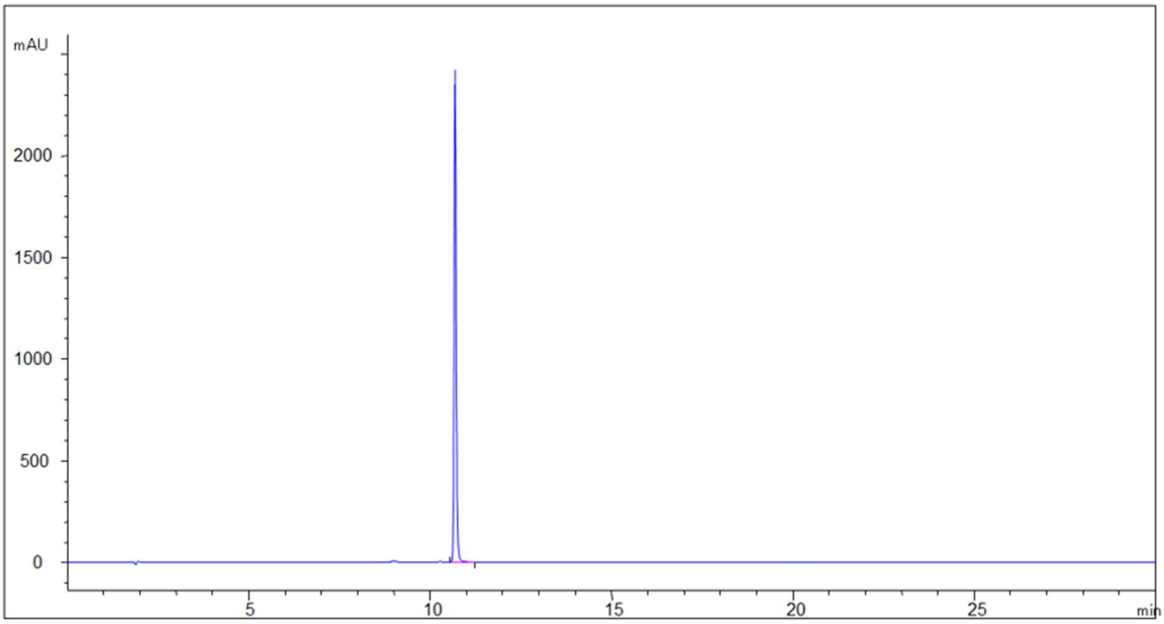

Figure S2. Chromatographic profile of **27** monitored at λ=230 nm.

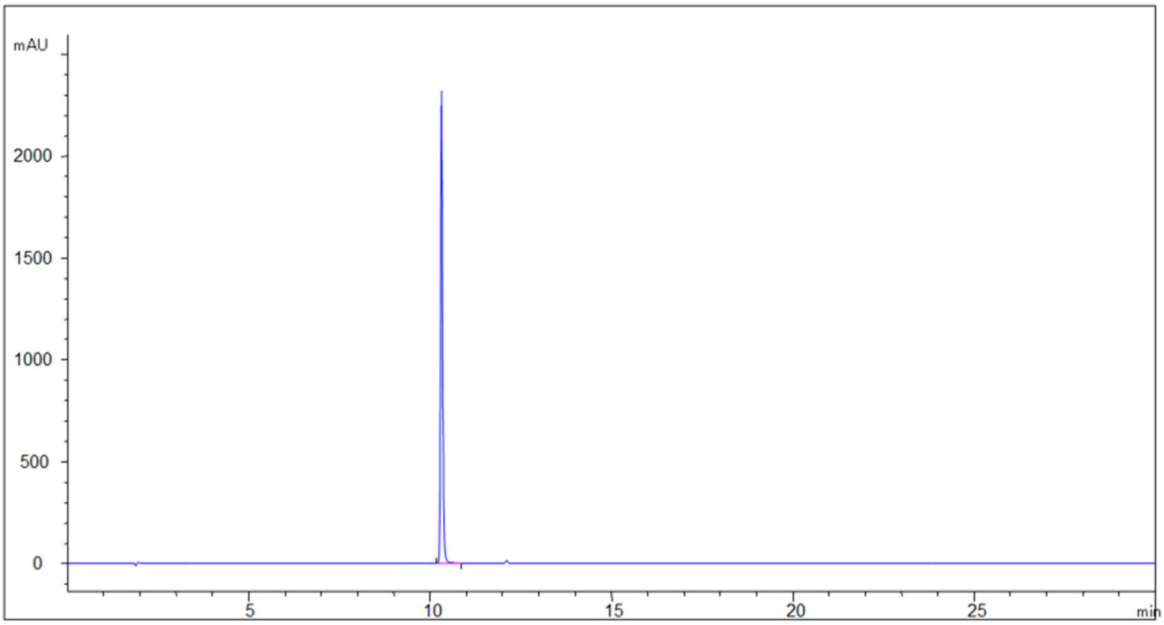

Figure S3. Chromatographic profile of **28** monitored at λ=230 nm.

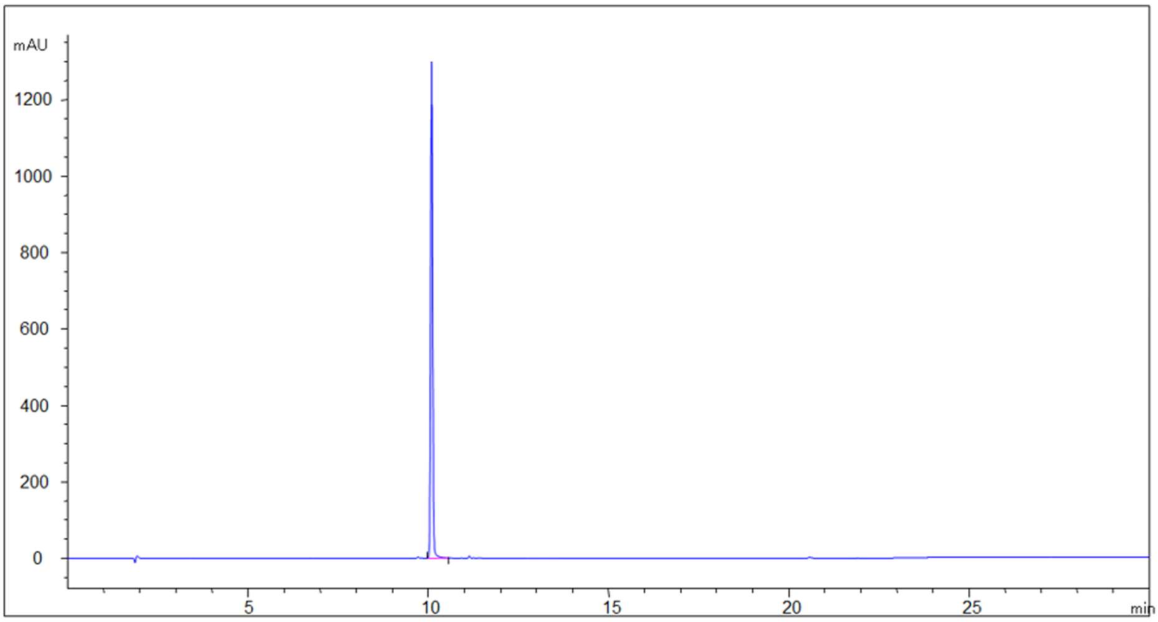

Figure S4. Chromatographic profile of **29** monitored at λ=230 nm.

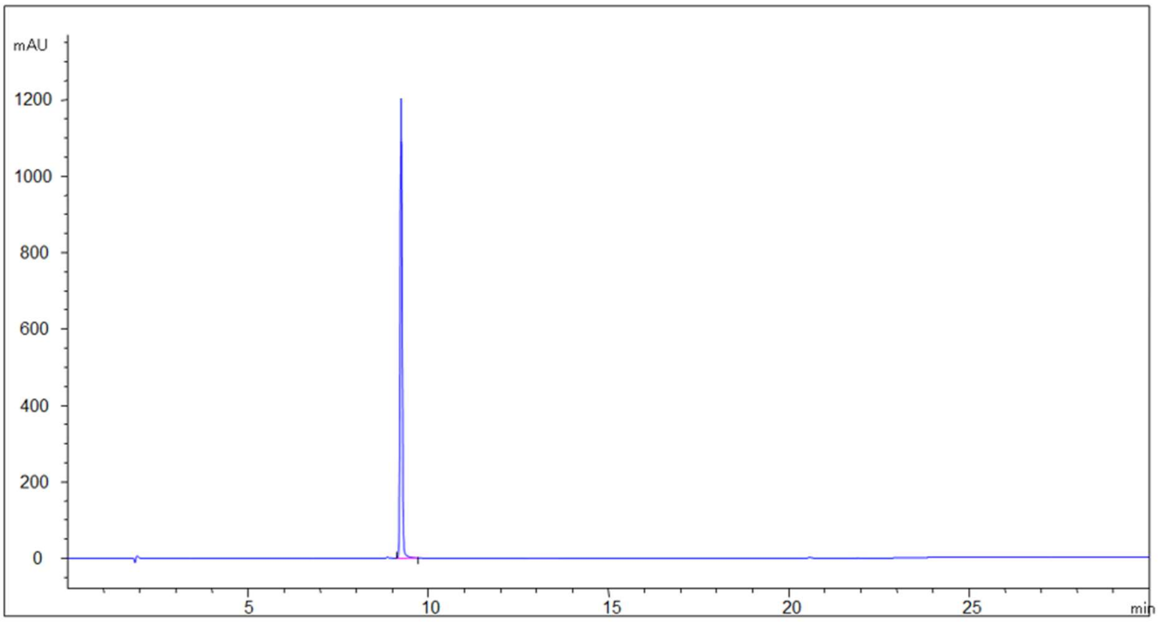

Figure S5. Chromatographic profile of **30** monitored at λ=230 nm.

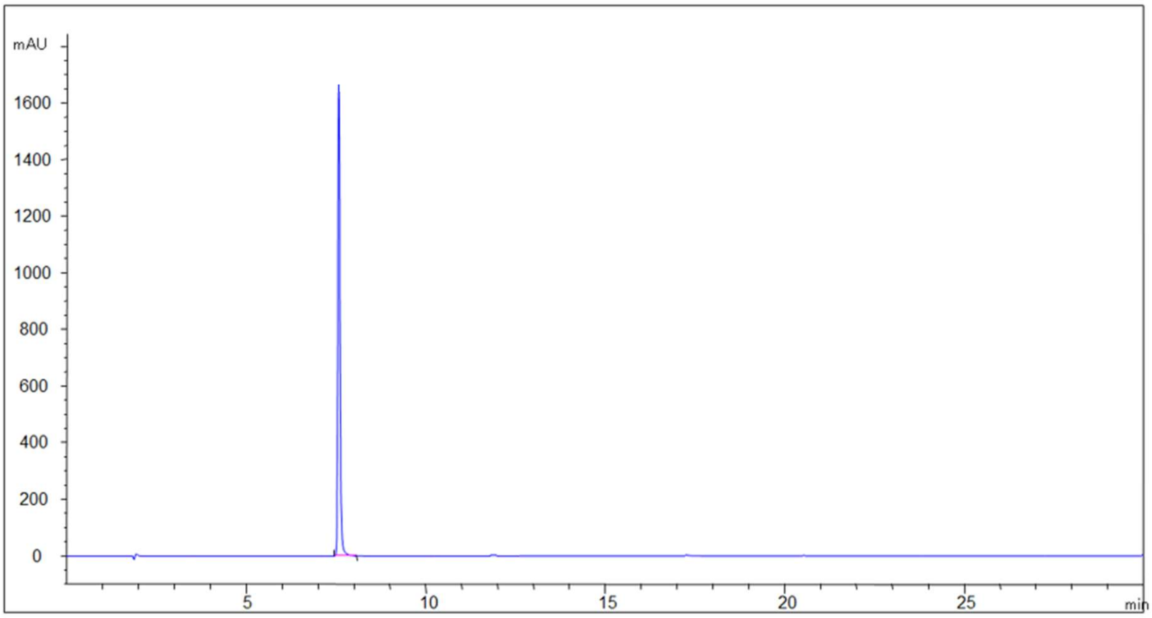

Figure S6. Chromatographic profile of **31** monitored at λ=230 nm.

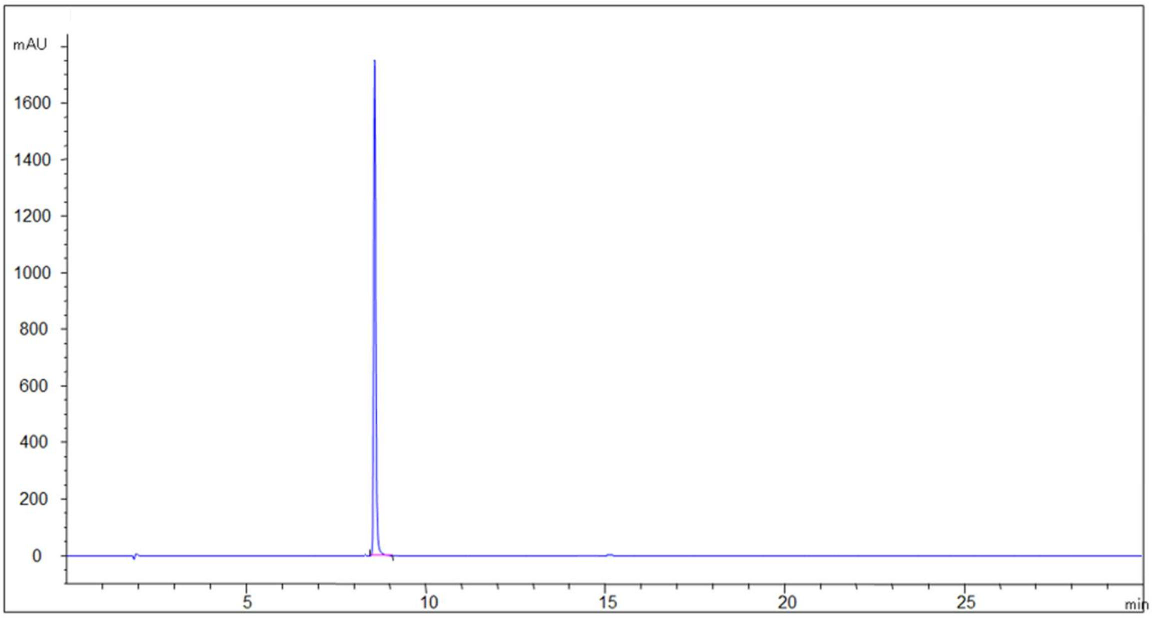

Figure S7. Chromatographic profile of **32** monitored at λ=230 nm.

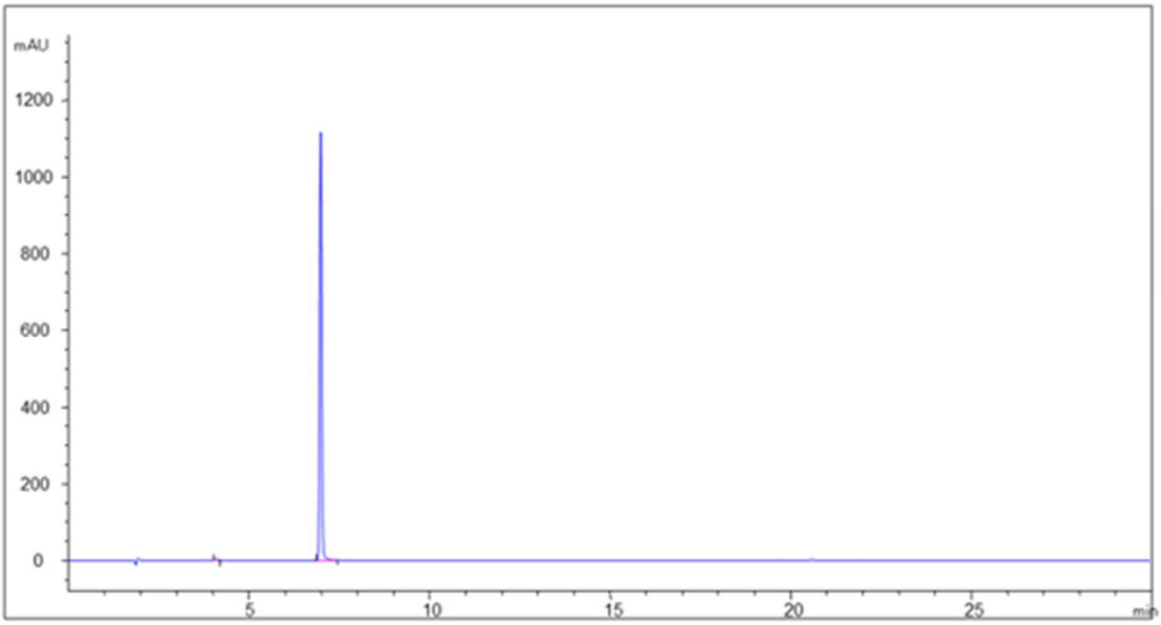

Figure S8. Chromatographic profile of **33** monitored at λ=230 nm.

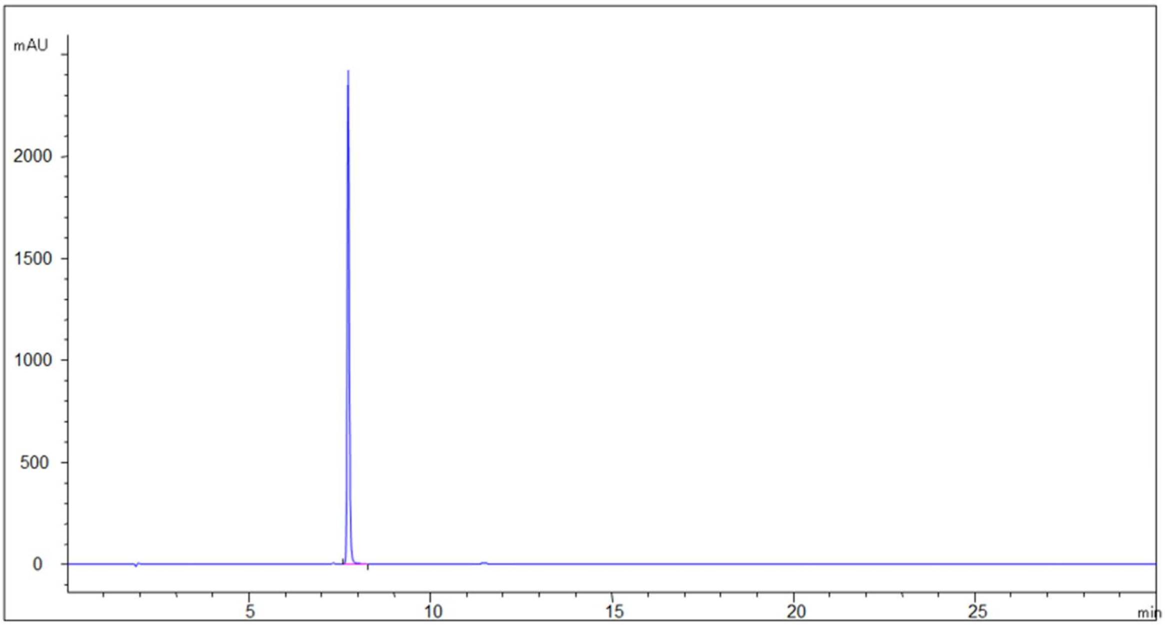

Figure S9. Chromatographic profile of **34** monitored at λ=230 nm.

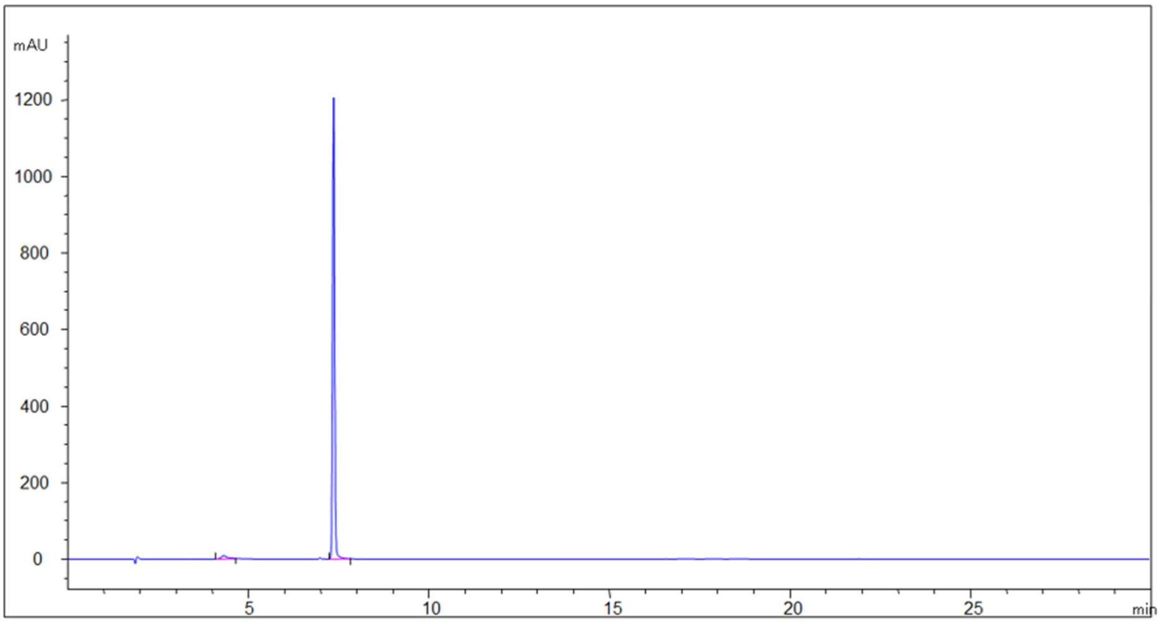

Figure S10. Chromatographic profile of **35** monitored at λ=230 nm.

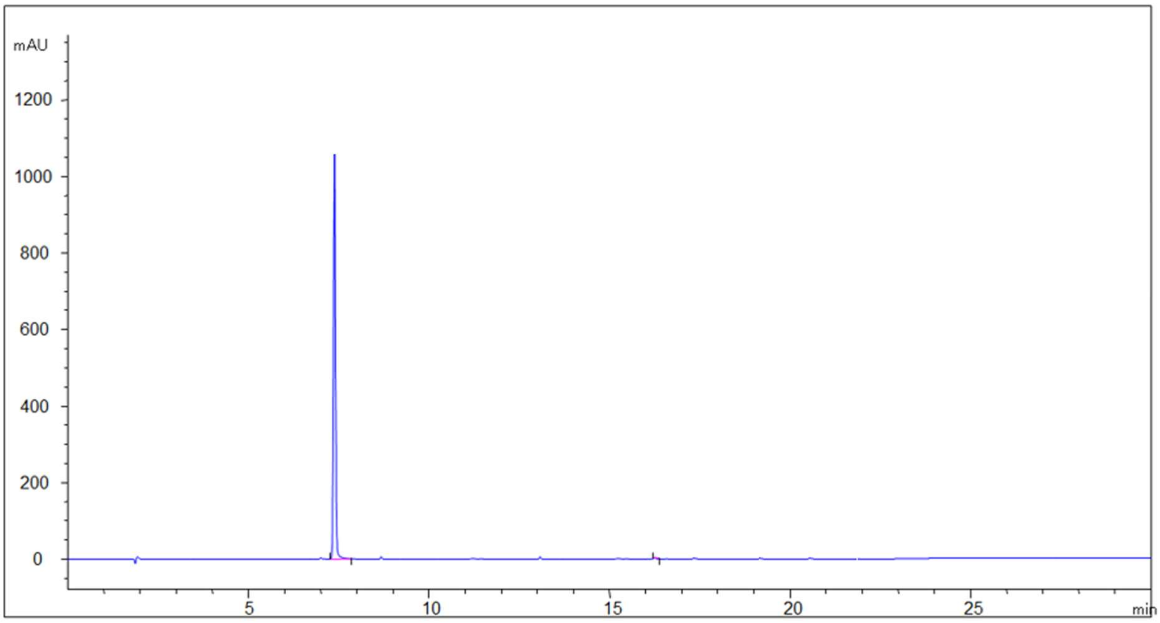

Figure S11. Chromatographic profile of **36** monitored at λ=230 nm.

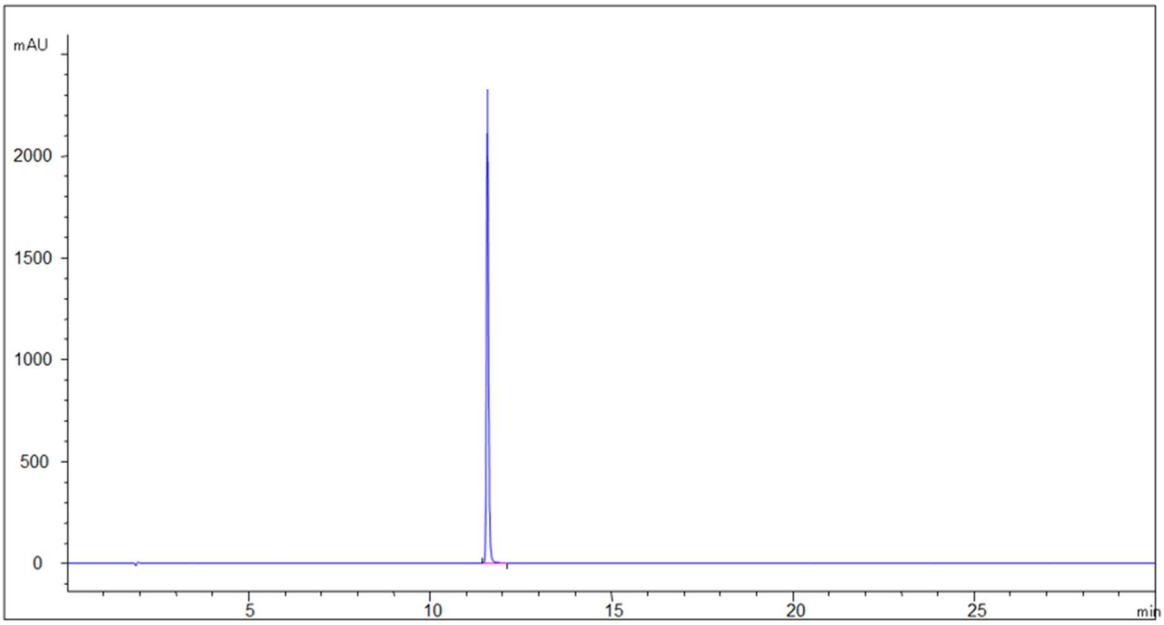

Figure S12. Chromatographic profile of **37** monitored at λ=230 nm.

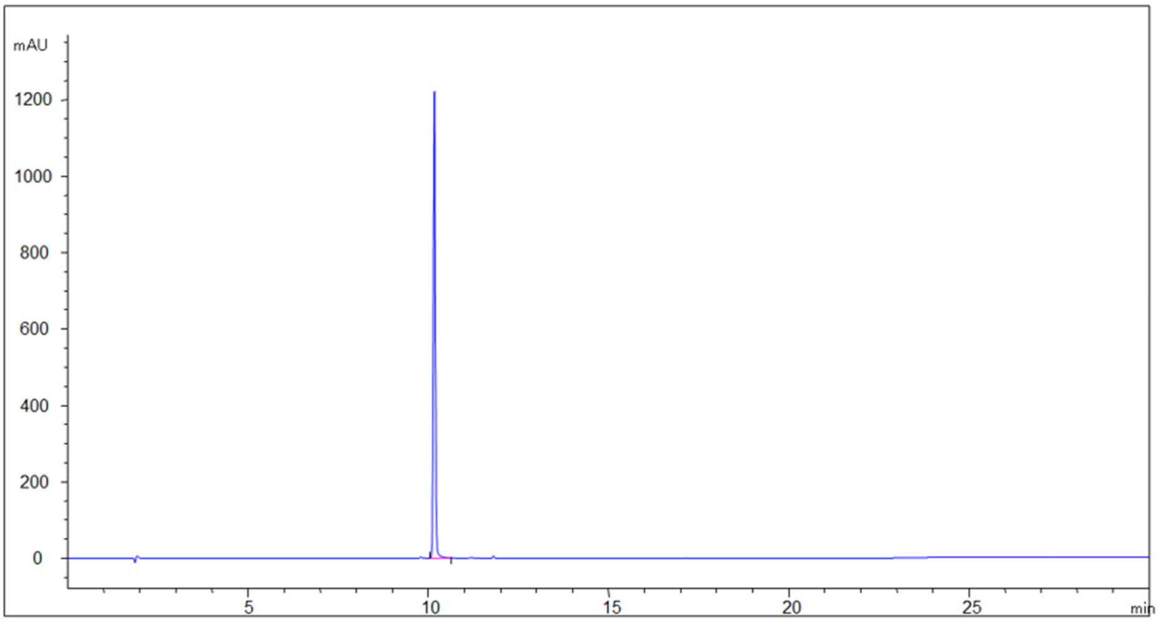

Figure S13. Chromatographic profile of **38** monitored at λ=230 nm.

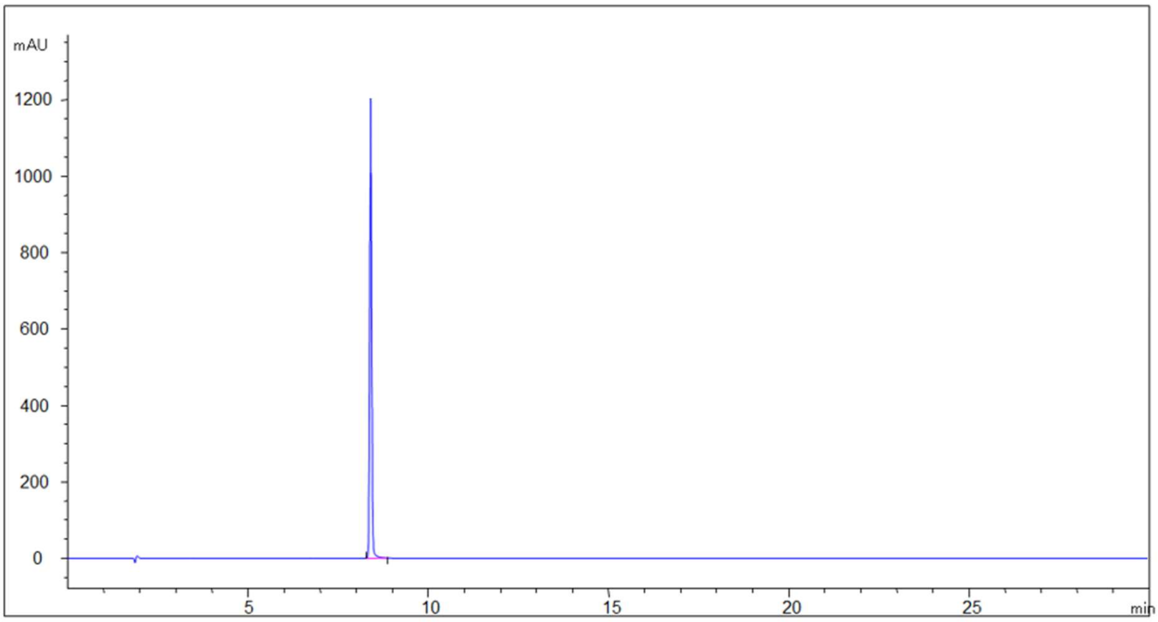

Figure S14. Chromatographic profile of **39** monitored at λ=230 nm.

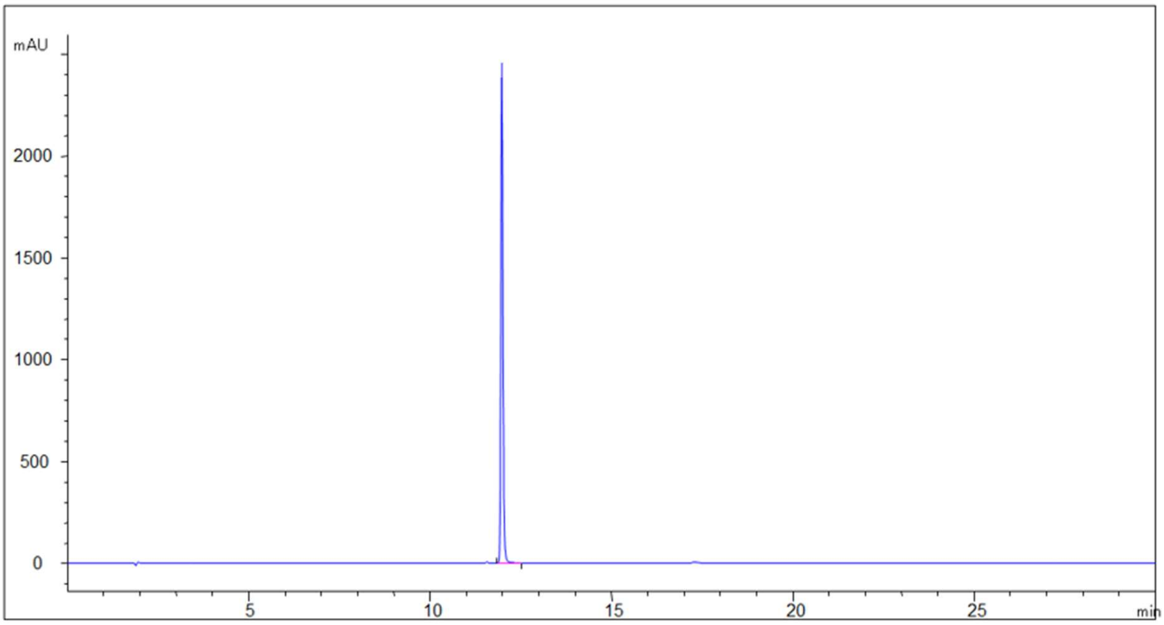

Figure S15. Chromatographic profile of **40** monitored at λ=230 nm.

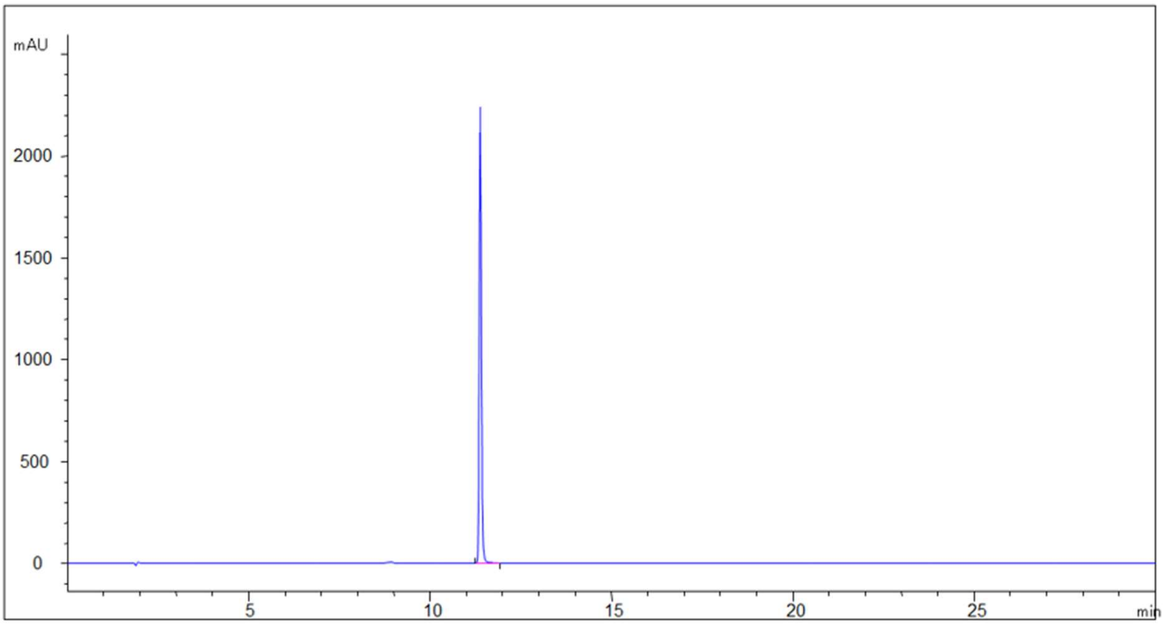

Figure S16. Chromatographic profile of **41** monitored at λ=230 nm.

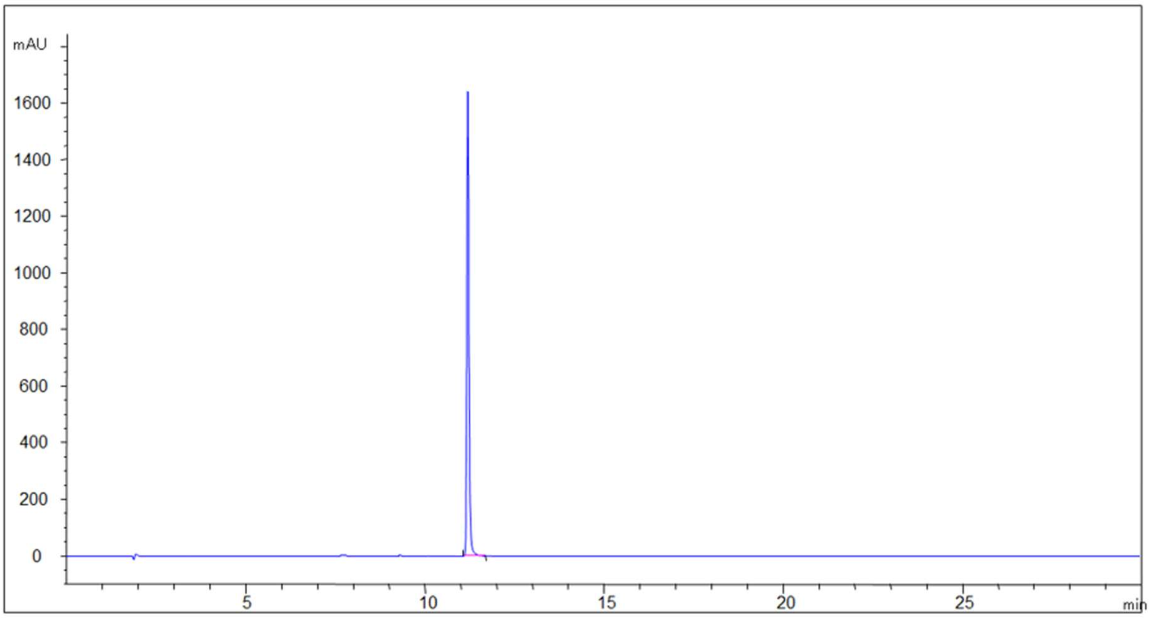

Figure S17. Chromatographic profile of **42** monitored at λ=230 nm.

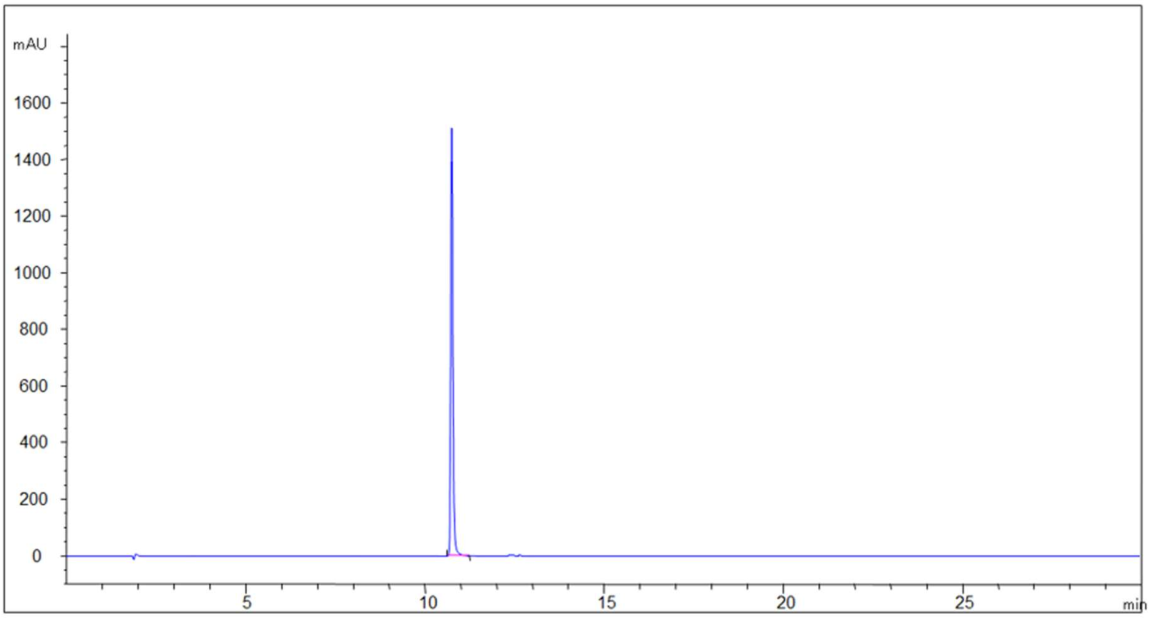

Figure S18. Chromatographic profile of **43** monitored at λ=230 nm.

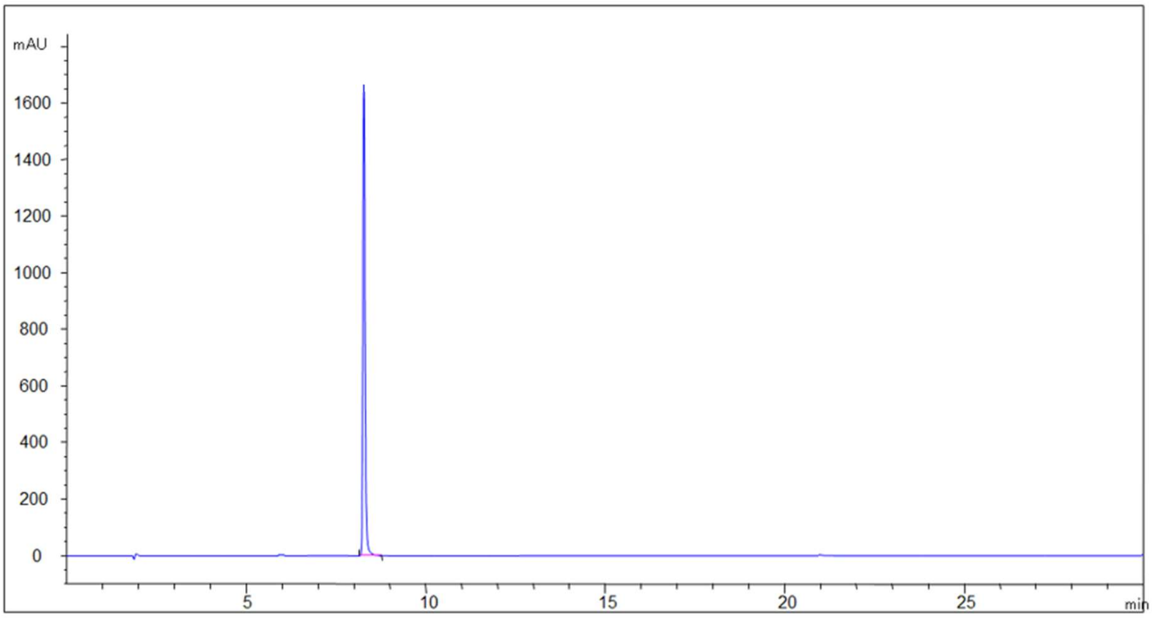

Figure S19. Chromatographic profile of **44** monitored at λ=230 nm.

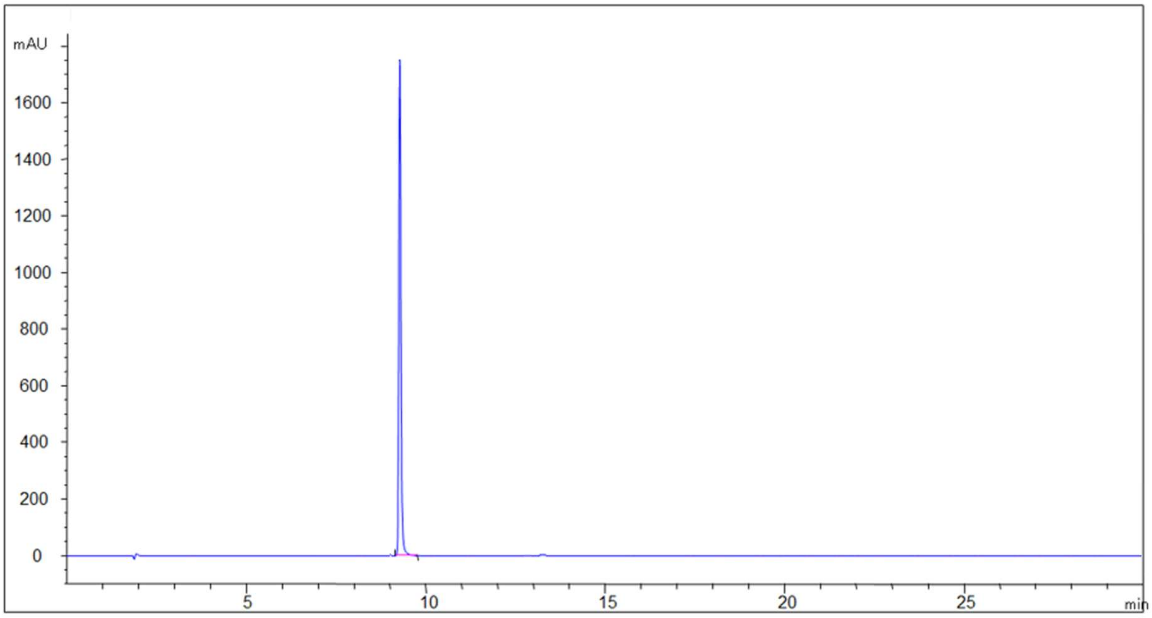

Figure S20. Chromatographic profile of **45** monitored at λ=230 nm.

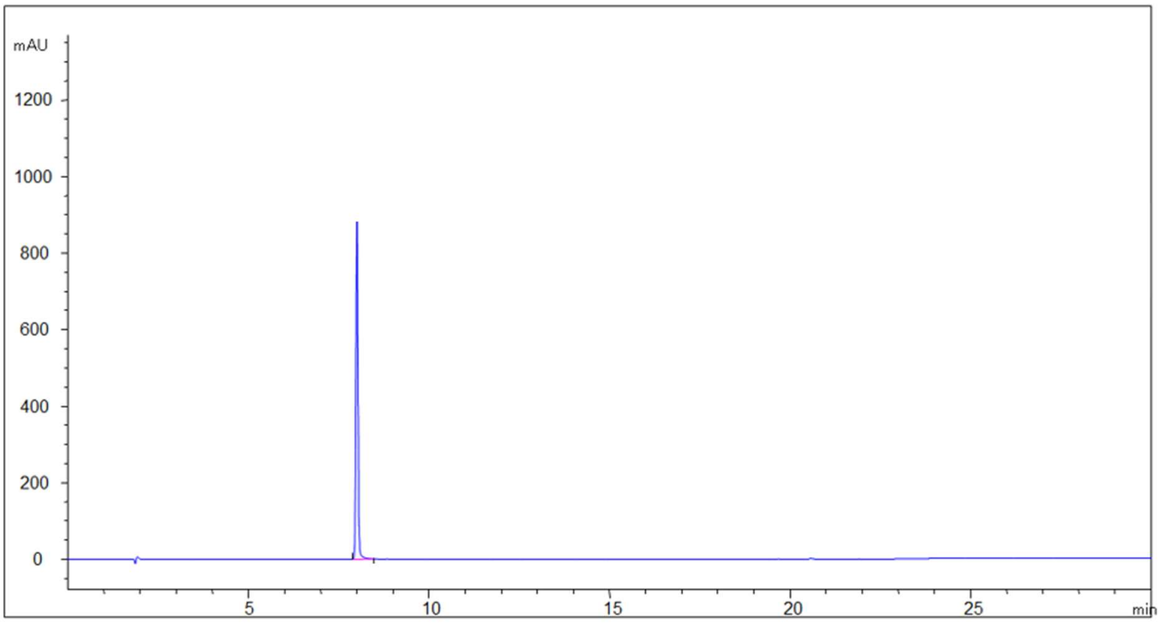

Figure S21. Chromatographic profile of **46** monitored at λ=230 nm.

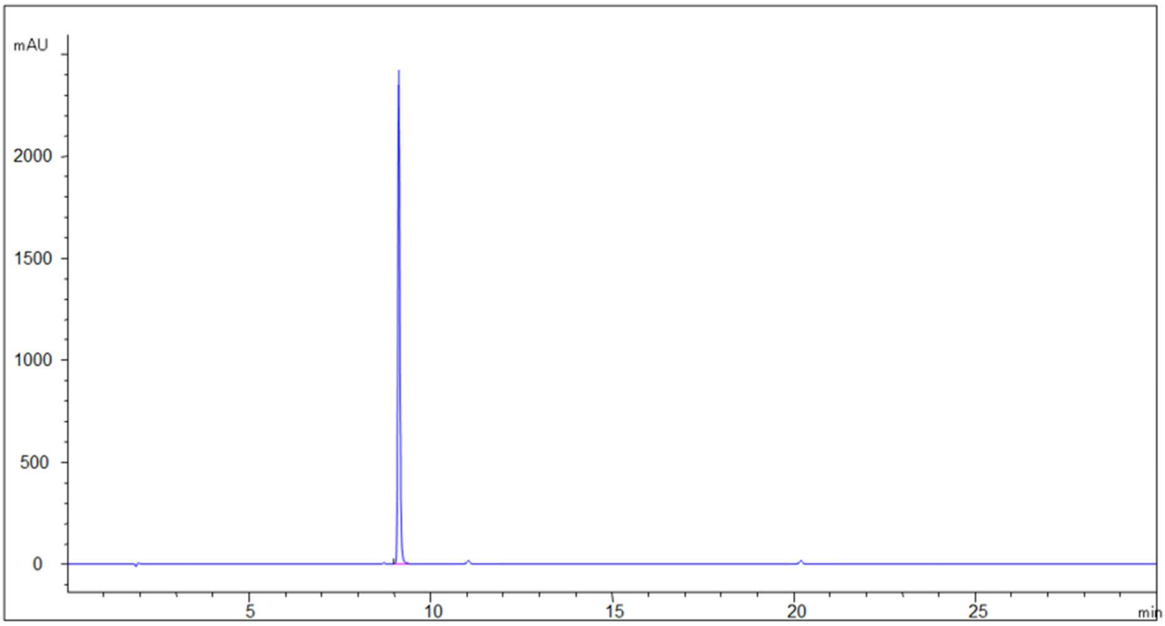

Figure S22. Chromatographic profile of **47** monitored at λ=230 nm.

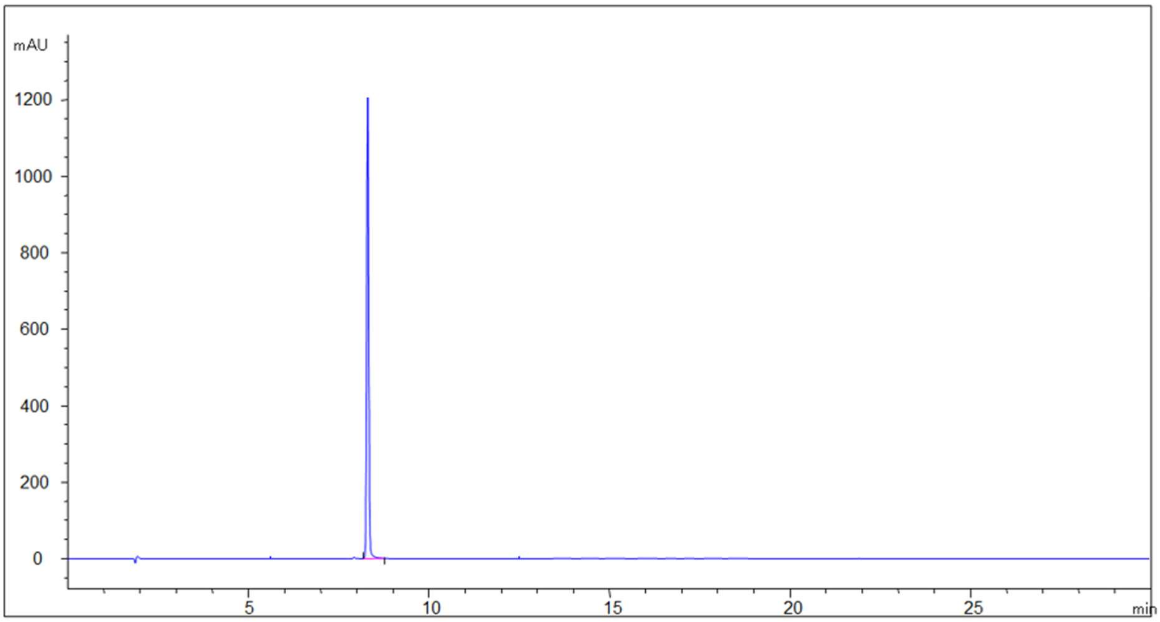

Figure S23. Chromatographic profile of **48** monitored at λ=230 nm.

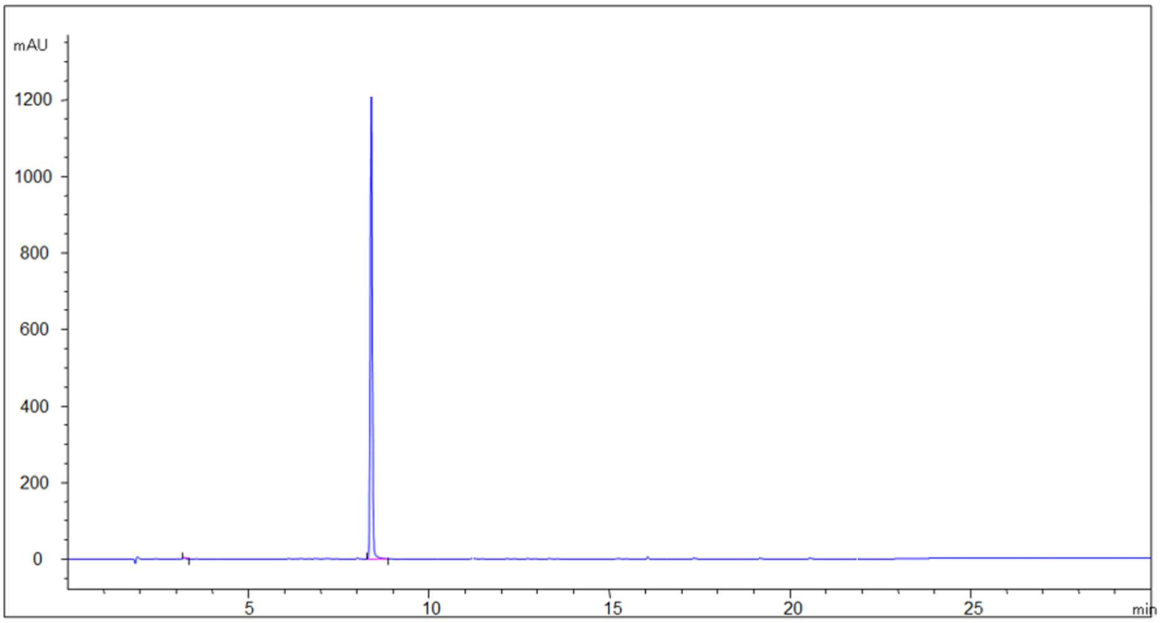

Figure S24. Chromatographic profile of **49** monitored at λ=230 nm.

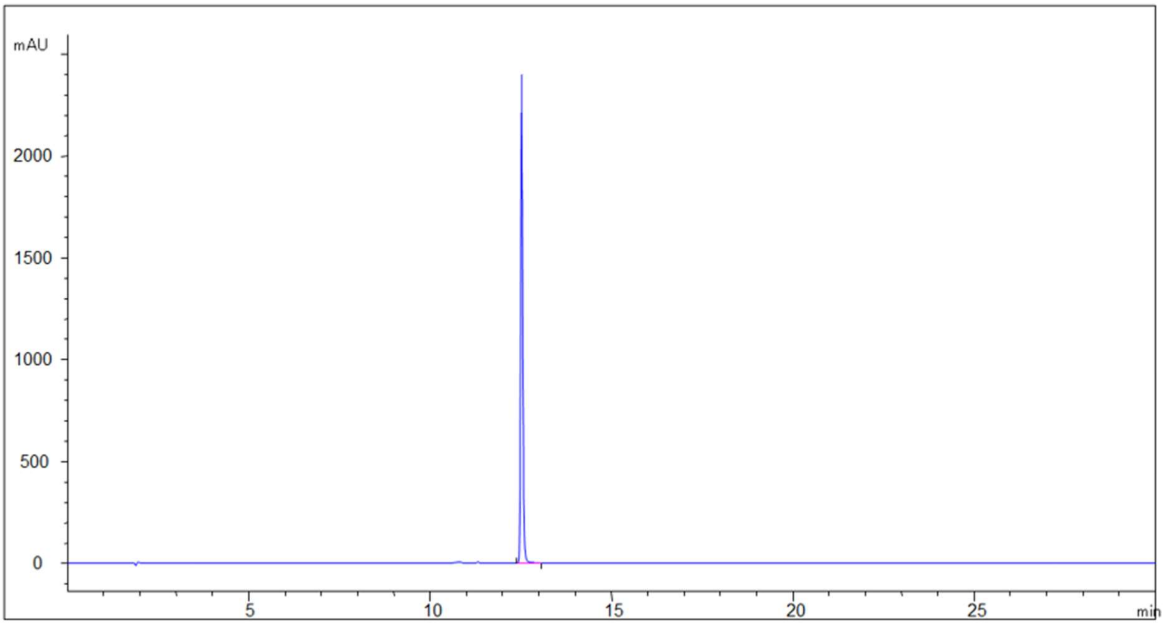

Figure S25. Chromatographic profile of **50** monitored at λ=230 nm.

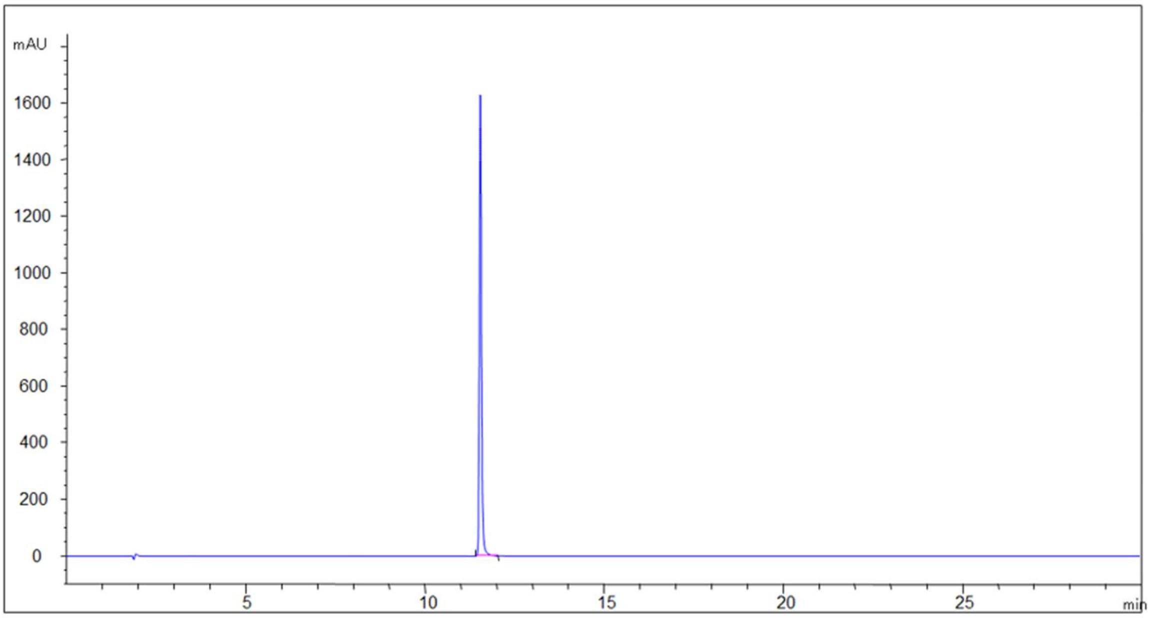

Figure S26. Chromatographic profile of **51** monitored at λ=230 nm.

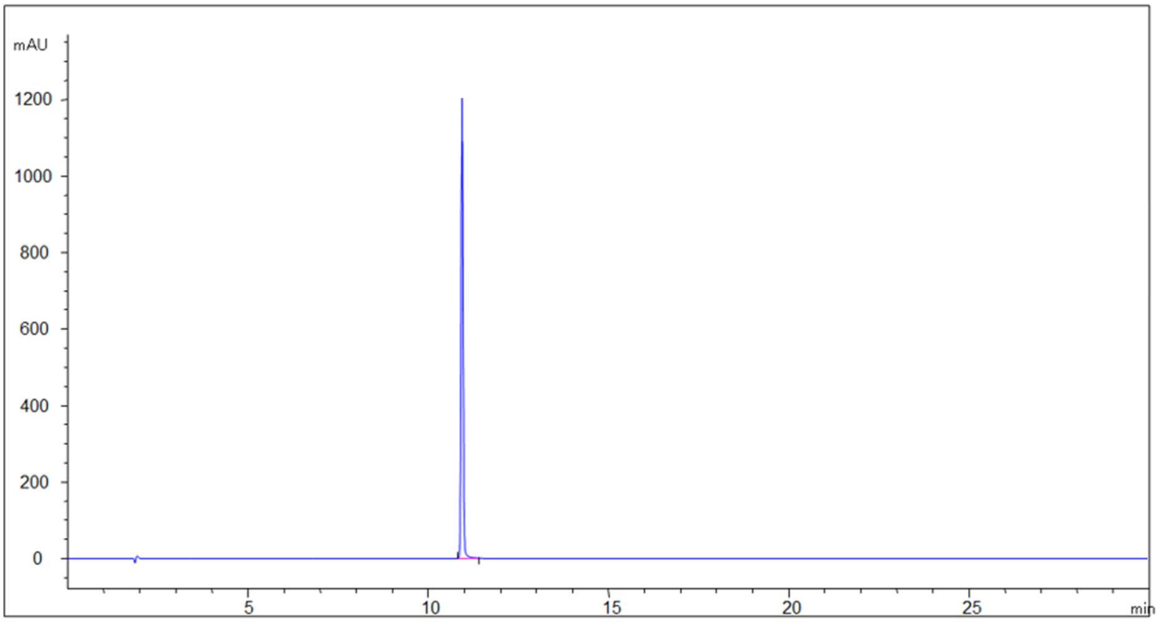

Figure S27. Chromatographic profile of **52** monitored at λ=230 nm.
